# Mutation and selection explain why many eukaryotic centromeric DNA sequences are often A + T rich

**DOI:** 10.1093/nar/gkab1219

**Published:** 2021-12-20

**Authors:** Anne C Barbosa, Zhengyao Xu, Kazhal Karari, Wendi Williams, Silke Hauf, William R A Brown

**Affiliations:** School of Life Sciences, University of Nottingham, Queen's Medical Centre, NG7 2UH, UK; School of Life Sciences, University of Nottingham, Queen's Medical Centre, NG7 2UH, UK; School of Life Sciences, University of Nottingham, Queen's Medical Centre, NG7 2UH, UK; Virginia Tech, Department of Biological Sciences, Fralin Life Sciences Institute, 1015 Life Science Circle, Blacksburg, VA 24061, USA; Virginia Tech, Department of Biological Sciences, Fralin Life Sciences Institute, 1015 Life Science Circle, Blacksburg, VA 24061, USA; School of Life Sciences, University of Nottingham, Queen's Medical Centre, NG7 2UH, UK

## Abstract

We have used chromosome engineering to replace native centromeric DNA with different test sequences at native centromeres in two different strains of the fission yeast *Schizosaccharomyces pombe* and have discovered that A + T rich DNA, whether synthetic or of bacterial origin, will function as a centromere in this species. Using genome size as a surrogate for the inverse of effective population size (N_e_) we also show that the relative A + T content of centromeric DNA scales with N_e_ across 43 animal, fungal and yeast (Opisthokonta) species. This suggests that in most of these species the A + T content of the centromeric DNA is determined by a balance between selection and mutation. Combining the experimental results and the evolutionary analyses allows us to conclude that A + T rich DNA of almost any sequence will function as a centromere in most Opisthokonta species. The fact that many G/C to A/T substitutions are unlikely to be selected against may contribute to the rapid evolution of centromeric DNA. We also show that a neo-centromere sequence is not simply a weak version of native centromeric DNA and suggest that neo-centromeres require factors either for their propagation or establishment in addition to those required by native centromeres.

## INTRODUCTION

Centromeres, despite mediating the evolutionarily conserved function of directing chromosome segregation, show surprising diversity in their DNA sequence organization (1–4). Single-celled eukaryotes illustrate this paradox vividly (4). In some budding yeasts, the fraction of A + T nucleotides in the centromeric DNA is greater than 0.8 and the centromere is confined to a few hundred base pairs (5–7). In contrast, the centromeres of the pathogenic yeast *Candida albicans* show no A + T enrichment, occupy several kilobases of DNA (8) and can form at a wide variety of positions in the genome (9). Metazoan organisms also have diverse centromeric DNA sequences. Although in many metazoan species all of the centromeres in a karyotype are associated with tandemly repetitive DNA (10), there are some species (11,12) that have one or more centromeres associated with single-copy sequences that bear no obvious relationship to the tandemly repeated centromeric DNA. Such centromeres are called neo-centromeres or evolutionarily novel centromeres. Centromeric DNA, therefore, poses three questions. First, why is it so diverse, second, what is the relationship between native centromeres and neo-centromeres or evolutionarily novel centromeres and third, why is it often but not always A + T rich?

Two answers to the question of why centromeric DNA is so diverse have been proposed. The first suggests that centromeric DNA can evolve with limited selective constraint because the kinetochore, the protein complex that binds the centromeric DNA, is inherited epigenetically. A central role of epigenetic processes in kinetochore inheritance was established by experiments that showed that stable structures with the features of kinetochores formed at non-centromeric loci following ectopic localisation of either the centromere-specific histone, CENP-A/CenH3, other constituents of the constitutive centromere associated network (CCAN) or HJURP, the CENP-A/CenH3 chaperone (13–17). Centromere epigenetic inheritance has been characterized in detail in human cells (18–21) but in some of these studies, both DNA sequence and epigenetic inheritance were important for segregation accuracy (19) Thus it is not clear whether the lack of constraint on centromeric DNA sequence implicit in the epigenetic explanation is sufficient to account for the rapid evolution of the centromeric DNA. A second explanation for centromere diversity emerged from the recognition that, at least in metazoans, centromeres are capable of meiotic drive in females (3,22,23). This led to to a model (3) that consisted of two components. First, stronger centromeres are favoured to partition to the egg as a result of drive (22–25) and consequently tend to fix in the population. Second, selection against strong centromeres occurs in male meiosis leading to the emergence of suppressors both within the centromeric DNA itself and in components of the kinetochore that antagonize the maladaptive features of the strong centromeres in the male. However, the centromere drive model does not explain the diversity of centromeres in organisms with symmetric meioses such as single-celled eukaryotes. Thus, the origin of centromere sequence diversity remains an unanswered question of fundamental significance.

The second and third questions set out above have also yet to be answered. Epigenetic mechanisms held in common with native centromeres are assumed to account for the stable inheritance of neo-centromeres however the evidence to justify this assumption is weak. Although the kinetochores at neo-centromeres contain many of the proteins present at native centromeres (26) they have never been fully characterized and the possibility that they contain one or more additional adaptor proteins that enable them to bind neo-centromeric DNA has not been excluded. Lynch and colleagues (27) have suggested that centromeric DNA is often A + T rich because reduced meiotic recombination at the centromere is accompanied by reduced mismatch repair which is often but not always GC biased. However, the correlation between centromeric A + T content, recombination suppression at the centromere and the sequence bias of mismatch repair has not been tested.

The sequence requirements for centromere function have in the past been mainly studied by the transformation of live cells with either plasmids (28,29) (in the case of single-celled eukaryotes) or bacterial artificial chromosomes (30) (in the case of human cells) containing candidate sequences. The centromeric activity of the candidate sequence has then been assayed by recovery of stably transformed cells and analysis of the transforming DNA for autonomous stable inheritance or binding of centromere proteins. Although it is often assumed that recovery of accurately segregating transforming DNA in these experiments is due to *de novo* centromere formation it is also possible that any centromeric activity present on the transformed DNA was acquired by the acquisition in *trans* of an epigenetic mark from pre-existing resident centromeres. The plasmid-based approach also suffers from the fact that even centromeric plasmids show higher levels of missegregation than native chromosomes and have variable copy numbers (31). Consequently, quantitation of centromere protein binding has used ‘semi-quantitative methods’ and the analysis of centromeric plasmid segregation by live-cell imaging has not, to our knowledge, been reported. In light of such profound mechanistic uncertainties and technical limitations inherent in the plasmid-based approach, we have approached the problem by manipulating the DNA at the centromeres of the chromosomes themselves using chromosome engineering approaches. The genetically tractable fission yeast *Schizosaccharomyces pombe* is ideal for applying such an approach because its centromeres are similar to the regional centromeres of many other eukaryotes (32,33) and because it can form neo-centromeres (34). These potential advantages have not, until now, been exploited for two reasons. Firstly until recently, there was no easy way of serially integrating and deleting DNA in *S. pombe* and secondly, the centromeres of the commonly-used laboratory strain of *S. pombe* are flanked by palindromically organized heterochromatin that makes sequence manipulations of centromeric DNA difficult. The first limitation was overcome by the development of a set of functionally optimized unidirectional serine recombinases (35,36). Unidirectional recombinases are easier to use for integration reactions than reversible recombinases such as *Cre* recombinase (see (37) for a summary of strategies for using *Cre* for integration reactions) because one does not have to contend with the reverse, excision reaction. Unidirectional recombinases are also preferred for deletion reactions when one of the products is unstable and one needs to be able to measure and interpret the kinetics of the reaction process because doing so requires fewer assumptions. That the second limitation may no longer apply was suggested by the discovery of a natural isolate of *S. pombe* (CBS 2777) (38) that has two acrocentric centromeres (39) that lack flanking heterochromatin. Here we applied chromosome engineering techniques to one of the acrocentric centromeres of CBS 2777 and then to one of the complex centromeres of the laboratory strain. We showed that three pieces of randomly chosen bacterial DNA that have nucleotide contents equal to or greater than 0.747 A + T can function as centromeres in *S. pombe*. Similarly, a synthetic sequence constructed by embedding A + T rich sequences from *Saccharomyces cerevisiae* centromeres into the *S. pombe* wee1 gene generated a sequence of 0.74 A + T content which also functioned as a centromere.

The fact that centromeric DNA is often A + T rich, when considered together with our observations suggested that the sequence composition of centromeric DNA is determined, in part, by a balance between mutation, that drives the A + T content towards the genome average and selection for A + T richness. We tested this idea by analysing the extent of the A + T enrichment of centromeric DNA as a function of genome size where genome size is used as a surrogate for 1/*N*_e_, the reciprocal of the effective population size (40) for 43 Opisthokonta (yeast, fungal and animal) species. This showed that centromeric DNA from organisms with large effective population sizes, such as single-celled eukaryotes, was, on average, more A + T rich relative to the respective genome than that of organisms with small effective population sizes such as mammals. This result is consistent with a ‘mutation-selection balance’ model for the evolution of the sequence content of centromeric DNA and implies that our discovery, that A + T rich DNA will function as a centromere in *S. pombe*, is true for most Opisthokonta species.

These results when combined suggest that, despite the diversity of centromeric DNA, centromeric activity in many eukaryotes may only require a stretch of DNA that is sufficiently A + T rich. Correspondingly a single member of a large set of A + T rich sequences is likely to have centromere function in a wide variety of eukaryotes.

We also tested whether a neo-centromere sequence could function as a centromere when placed at a native centromere and show that it could not. This result suggests that native centromeres and neo-centromeres are either established or maintained by different mechanisms in *S. pombe*.

Our experiments were concerned with sequence specificity. However, our approach can, in combination with genetic analysis, be extended to analyse the importance of centromere size and thus to test many of the ideas underpinning the centromere drive model. The recombinases that we used work well in many cell types and our technical approach should be broadly applicable, particularly in unicellular eukaryotes other than *S. pombe*, allowing our ideas to be tested and extended.

## MATERIALS AND METHODS

### Engineering a chromosome in *S. pombe* strain CBS 2777 to enable assay of the centromere activity of a candidate sequence

To assay the ability of a sequence to function as a centromere we first needed to engineer a chromosome containing the sequence in question in a defined position with respect to the pre-existing centromere. In most of the experiments, this involved the construction of a chromosome that contained the candidate centromeric sequence placed adjacent to the pre-existing centromere which was in turn flanked by attachment sites for the Bxb1 integrase (Supplementary Figure S1). This was achieved through the following three steps:

- The ura4 gene flanked by attachment B sites for the ϕC31 integrase was integrated between residues 131490 and 131491 adjacent to the breakpoint 4 sequence on the right-hand side of the centromere of chromosome 2 of CBS 2777 ura4^Δ^ leu1^Δ^ kanMX6-Pcnp1-mEGFP-cnp1 (Nott363) to create strain Nott373 (Supplementary Table S7).
- Candidate sequences (see below) were cloned into the BamHI site of the vector pFA6a-natMX6 REV *attP^ϕ^^C31^ attP^ϕ^^C31^attB*^*Bxb1*^ (Supplementary Figure S2) and then integrated into the attB sites of breakpoint 4 construct in strain Nott373 using ϕC31 integrase expressed from a pREP1 construct encoding a codon-optimized form of the integrase gene tagged with a nuclear localization sequence. Integration of the candidate sequence cloned in pFA6a-natMX6 REV *attP^ϕ^^C31^ attP^ϕ^^C31^attB*^*Bxb1*^ was accompanied by loss of the ura4 marker and acquisition of resistance to the antibiotic nourseothricin which was used as the basis of an initial screen for site-specific integration. The empty pFA6a-natMX6 REV *attP^ϕ^^C31^ attP^ϕ^^C31^attB*^*Bxb1*^ vector was integrated as a control. Integration was checked by PCR at both ends of the integrated DNA and the integrity of the integrated sequences was checked by long-range PCR and, in the early experiments, by agarose gel electrophoresis, filter transfer and hybridization. In the initial experiments aimed at establishing the system, site-specific recombination was also confirmed as reciprocal and conservative by recovering and sequencing the ϕC31integrase *attL* and *attR* sites (Anne Barbosa, PhD thesis University of Nottingham, 2017).
- The left-hand side (breakpoint 3) of the centromere of each strain containing a candidate sequence was then targeted between positions 123238 and 123281 with the ura4 gene flanked by attachment P site for the Bxb1 integrase. The centromeres of chromosomes 2 and 4 are homologous and so it was necessary to screen for targeting and then to determine the chromosomal location of the targeted construct. The chromosomal mapping was carried out by long-range PCR using primers that were either complementary to the ϕC31 integrase *attL* site on chromosome 2 or to the un-engineered chromosome 4.

### Assaying the ability of a candidate sequence to support centromere formation using site-specific recombination in *S. pombe* CBS 2777

The assay of the ability of a sequence to function as a centromere consisted of the deletion of the pre-existing centromeric DNA using the Bxb1 integrase (Supplementary Figure S3) and measurement of the efficiency with which cells containing the swapped centromere were recovered. The assay procedure consisted of the following steps

- Transform the engineered strain containing the candidate sequence with either pREP81 or pREP81-Bxb1.
- Plate out onto pombe minimal glucose (PMG) agar (41), supplemented with uracil.
- After 7 days, pick and pool ∼50 random colonies.
- Count the number of recovered cells per colony.
- Estimate viability by plating on YES agar.
- Measure the proportion of recovered cells that are uracil auxotrophic.
- Measure the proportion of uracil auxotrophic cells that have swapped by PCR, confirm the integrity of the candidate sequence by long-range PCR or blotting. Check the integrity of the chromosome by pulsed-field gel electrophoresis (PFG).

Further details of each step are described in the supplementary data sections 1.2 and 1.4. For details of the media and the methodologies of *S. pombe* culture see (41). Details of the sequences assayed are in supplementary data section 2 and listed in Supplementary Tables S1 and S2. Supplementary Figures S4–S6 describe the data used to validate the assay and Supplementary Table S3 provides the raw data used in Figure 2B of the main paper. Supplementary Figure S8 shows the sequence organization of the AT-rich wee1-CDEII concatamer described in Figure 4 of the main paper. Supplementary data section 1.4 and Figure 7 also provide a discussion and data underpinning our understanding of the reasons why cells with short (4.17 kb) centromeres are recovered at reduced efficiencies in this assay.

### Engineering chromosome II of the laboratory strain

The sequence of manipulations used to engineer the centromere of chromosome II of the laboratory strain is explained graphically in Figure 10 of the supplementary data. We started the sequence of chromosome engineering by targeting a *ura4* gene flanked by two *attB* sites for the ϕC31 integrase to residue 1,660,000 on the right-hand side of the centromere of chromosome II of a *his7* ^–^ derivative of the laboratory strain of fission yeast; (PN4576 h + leu1 ura4 his7 alp16Δ::kanR). We used this as a ‘landing pad’ to introduce an array of 240 lac operator sequences derived from plasmid pLAU43 (42) between the *attB* sites using the plasmid pFA6a-natMX6 REV *attP^ϕ^^C31^ attP^ϕ^^C31^*as the vector for the incoming sequence. The integration reaction simultaneously deleted the *ura4* gene. We crossed this strain with WRAB_LS8 (h- *ade6*m210 *ura*4Δ18 *leu*1-32) and derived a strain (h- his7 chr II:1,660,000: lacO∼240 Nat^r^ ura4 leu1 ade6 kanS). We crossed this strain with SI460 (h + leu1 ade6 m216 cen2 << lacO<< *ura4+* << kan^r^*his7+* <<GFP-LacI∼nls) and derived a strain Nott979 with the genotype; h90 *his + ura4- leu1-* chr II:1,660,000: lacO∼240 Nat^r^ G418^s^ This strain was checked by microscopy for segregating GFP fluorescence and was used as the basis of all further manipulations as it contained both the pericentromeric lacO array and the transgene expressing the lacI∼GFP ligand necessary for imaging of the centromere of chromosome II. We then targeted the sequences flanking the gene conferring resistance to nourseothricin with a *ura4* gene flanked by *attP* and *attB* sites for the ϕC31 integrase thus deleting the gene conferring resistance to nourseothricin. We then deleted the *ura4* gene using expression of the ϕC31 integrase. We introduced a *ura4* gene flanked by two *attB* sites for the ϕC31 integrase into the right-hand side of chromosome II at position 1 647 434, 12 566 residues 5′ of the lacO array, to generate strain Nott 986 and used this as a landing pad for 6.1kb of 0.778 A + T DNA cloned in the donor plasmid; pFA6a-natMX6 REV *attP^ϕ^^C31^ attP^ϕ^^C31^ attB^Bxb1^* used previously in the experiments with the CBS2777 strain which simultaneously deleted the *ura4* gene and led to the creation of strain Nott 1000. We then targeted a *ura4* gene flanked on its left-hand side by an *attB* site for the Bxb1 recombinase between residues 1 616 570 and 1 617 105 at the left-hand end of the left-most IMR in the centromere of chromosome II to generate two independent strain; 1030 and 1031. Expression of the Bxb1 integrase in these strains deleted the *ura4* gene, the centromere of chromosome II and the right-hand dGdH array and generated strains 1037 and 1038 respectively which we used in the imaging. The checks accompanying these manipulations are summarized diagrammatically and reported in Supplementary Figures S11 and S12, respectively.

### Labelling chromosome 2 of CBS2777 with tdTomato

To visualize the segregation of chromosome 2 in CBS 2777, we combined the tetO array/tetR-tdTomato labelling system of Watanabe and colleagues (43) with the site-specific recombination system described above. Thus we sub-cloned the 10kb tetO array of (43) into the pFA6a-natMX6 REV *attP^ϕ^^C31^ attP^ϕ^^C31^ attB*^Bxb1^ integration vector (Supplementary Figure S2) and then used ϕC31 integrase site-specific recombination to integrate the tetO array adjacent to the centromere in strain Nott373. To express the tetR-tdTomato fusion, we replaced the NatMX marker in pNAT ZA31-tetR-tdTomato of (43) with HygMX and targeted the Z locus between *zfs1* and SPBP7E8.01 on chromosome 2. The targeted cells were checked for the integrity of the tetO array by Southern blotting. A laboratory strain of *S. pombe* with the tetO array adjacent to the centromere of chromosome 2 at the D107 locus (44) was used as a reference. Both strains expressed *cnp1* N-terminally tagged with mEGFP (45).

### Live-cell imaging

**Figure 1.**
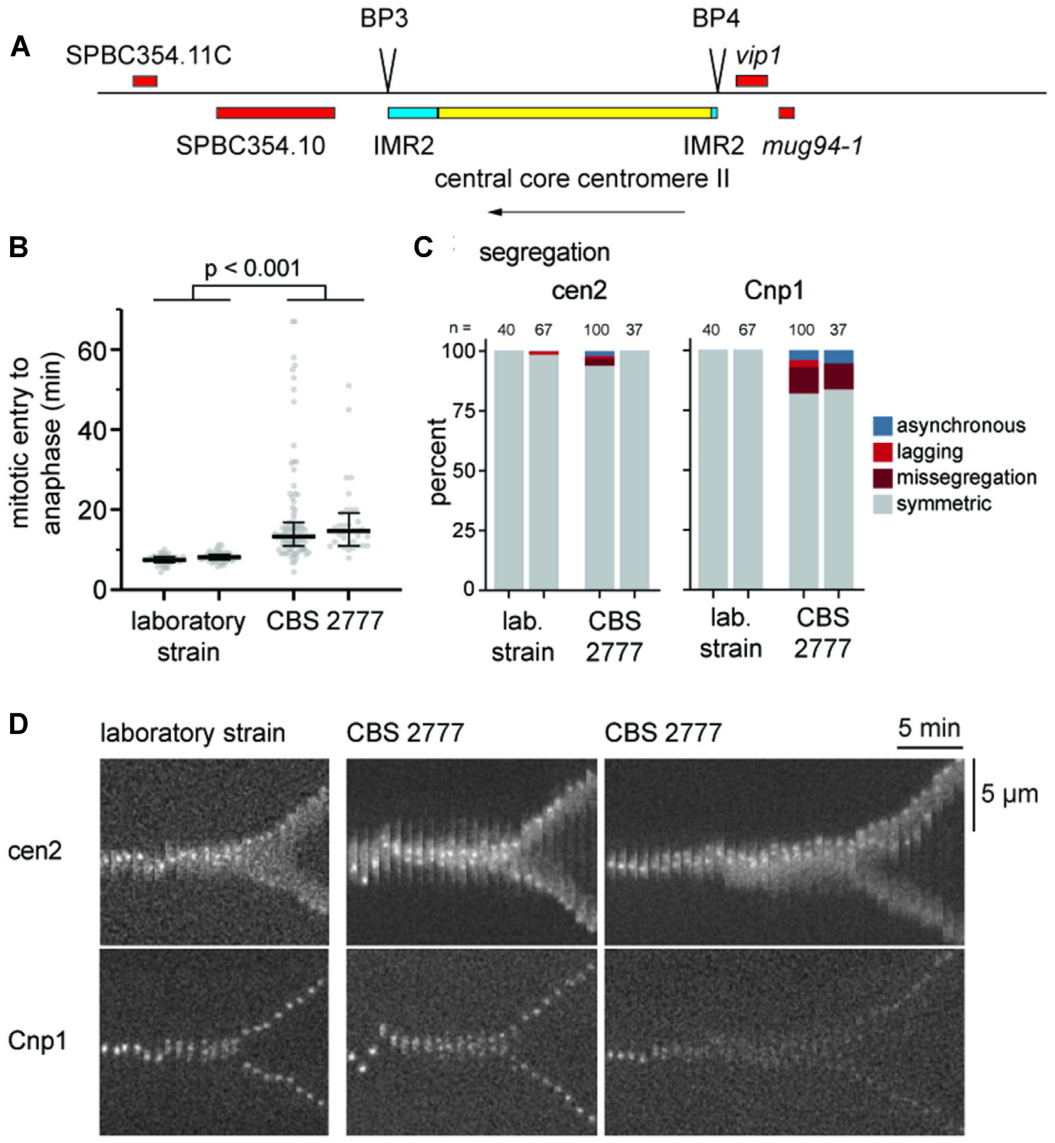
Segregation accuracy of the re-arranged centromere in *S. pombe* CBS 2777. (**A**) Sequence organization of the centromeres of chromosome 2 and 4 of S. pombe CBS 2777. (**B**) Time from mitotic entry to anaphase for two laboratory strains and two CBS 2777 strains in which chromosomes II (laboratory strain) and 2 (CBS 2777 strain) were marked using the tetO array / tetR-tdTomato fusion system, and CENP-A^Cnp1^ was tagged with EGFP. The declustering of CENP-A^Cnp1^ signals was scored as mitotic entry, the splitting of CENP-A^Cnp1^ as anaphase. Single cells (grey) with median and quartiles (black). Medians from left to right are 7.5 min (*n* = 35 cells), 8.2 min (*n* = 57 cells), 13.3 min (*n* = 95 cells), and 14.7 min (*n* = 35 cells). The difference between laboratory strains and CBS 2777 strains is statistically significant by Mann–Whitney test (*P* < 0.001). (**C**) Same experiment as in (B), but analyzed for the accuracy of chromosome segregation of either cen2 on the basis of the tdTomato signal or all centromeres on the basis of the CENP-A^Cnp1^ EGFP signal. For the CBS 2777 strains, 11 and 2 cells, respectively, were excluded from the analysis because they showed either no or, in a single case, more than one cen2-tdTomato signal. The meanings of the categories are as follows: missegregation: unequal segregation to daughter cells (see D); lagging: one centromere still at the centre late in anaphase; asynchronous: separation at different times in early anaphase; 3 out of 100 cells in one CBS 2777 strain missegregated the labelled chromosome 2. (**D**) Kymographs from single cells, illustrating correct segregation in the laboratory strain (left), correct segregation but prolonged mitosis in the CBS 2777 strain (middle) and chromosome missegregation in the CBS 2777 strain (right).

Cells were grown in YEA at 30ºC and mounted in either microfluidic plates (CellAsic, Y04C) with constant YEA media flow at 4 psi, or in lectin-coated 8-well μ Slides (Ibidi). Imaging was performed on a DeltaVision Elite system with the environmental chamber set to 30ºC. Images were recorded with a CoolSnap HQ2 or a PCO edge sCMOS camera using an Olympus 60×/1.42 Plan Apo oil objective, solid-state illumination at 461–489 nm and 529–556 nm, a QUAD DAPI/FITC/TRITC/Cy5 multi-band filter for separating excitation and emission, and 525/48 and 597/45 emission filters. Fluorescence images were recorded with a time lapse between 10 s and 2.5 min. Brightfield images to determine cell cycle time were recorded with a time lapse of 4 min. For the data in Figure 1, chromosome segregation was scored for both the tdTomato-labelled chromosome 2 and all EGFP-Cnp1 labelled chromosomes. In ‘symmetric’ segregation, red/green fluorescent dots separated symmetrically to the two daughter cells without showing lagging. ‘Missegregation’ was scored when either both red fluorescent dots segregated to the same daughter, or when green dot intensities were different in the two daughter cells. Chromatids remaining at the center during anaphase were scored as ‘lagging’, chromatids all moving to the poles but separating at different times during early anaphase was scored as ‘asynchronous’. Kymographs were assembled using a custom Matlab script. When Z-sections were recorded, a maximum projection of the images was used to create kymographs.

### Chromatin immunoprecipitation (ChIP) and bio-informatics

ChIP was carried out as previously described (39) using Abcam AB290 anti-GFP antibody and Dynabeads Protein-A (Invitrogen / Thermo Fisher 10001D) for the precipitation of the GFP tagged Cenp-A. Bio-informatic analysis of the ChIP∼seq data was carried out using the BWA-mem tool to generate bam files and the bio-conductor package in R to generate and plot coverage vectors. For further details see supplementary data section 3.2

### Primers and strains

The sequences of key primers and the genotypes of key strains are listed in Supplementary Tables S6 and S7 respectively.

### Evolutionary analysis of centromeric DNA content

The linear regression used in Figure 7 was carried out using R (Version 1.3.959) in R studio and the results were illustrated using the ggplot2 package (46). The data used are listed in Supplementary Table S5.

## RESULTS

### An *S. pombe* centromere without flanking heterochromatin segregates with high fidelity

The *S. pombe* strain CBS 2777 has a karyotype that is extensively re-arranged with respect to the karyotype of the laboratory strain and includes two centromeres, those of chromosomes 2 and 4, that lack flanking heterochromatin (39) (Figure 1A). We wanted to use sequence manipulation of one of these centromeres to investigate the size and sequence dependence of centromere function in *S. pombe* but before we could do this we needed to establish that the lack of centromere flanking heterochromatin did not significantly affect segregation accuracy at mitosis. Live cell imaging of CBS 2777 with a tdTomato-labelled chromosome 2 and GFP-labelled centromeric histone CENP-A^Cnp1^ showed that, although anaphase was delayed by about 5 min relative to the laboratory strain (Figure 1B), there was only a small increase in chromosome missegregation (Figure 1C and D). Despite the importance of heterochromatin for binding cohesin and preventing merotelic attachment (47,48), lagging chromosomes were not prominent. We concluded that the heterochromatin-deficient centromere on chromosome 2 of CBS 2777 is largely functional and therefore used it for centromere-replacement experiments.

### Native centromere replacement allows assay of centromeric DNA function

**Figure 2.**
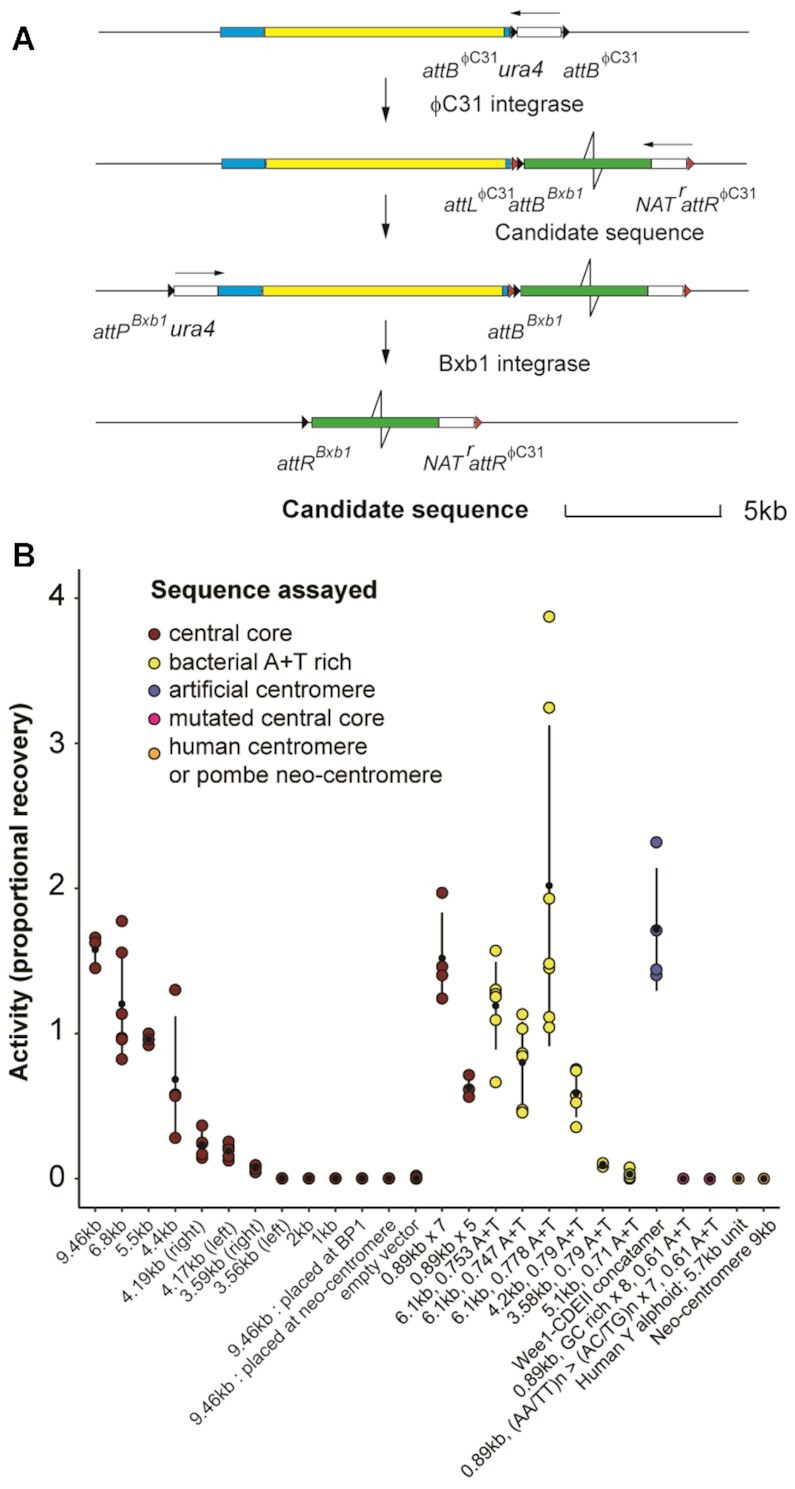
Centromere-replacement in *S. pombe* CBS 2777 as an assay for the centromeric activity of centromeric DNA and candidate sequences. (**A**) Sequence manipulations involved in centromere-replacement on chromosome 2 in CBS 2777. For a summary see the main text and for details see the Supplementary data. (**B**) Recovery of cells that had deleted the native centromeric DNA following transfection with a pRep81-Bxb1 integrase expression plasmid as a proportion of cells following transformation with an empty pRep81 expression vector for strains in which the different centromere sequences indicated in B were placed adjacent to the native centromere. The data are summaries of the mean and standard deviations of the results detailed in Supplementary Table S3. The 0.89 kb sequence was concatamerized into pentamers or heptamers of 4.4 and 6.2 kb. The assay and the observation that the maximum proportional recovery is greater than 1 is discussed in the main text and in more detail in the supplementary data. For details of the native centromere sequences assayed see Supplementary Table S1 and for details of the experimental sequences see Supplementary Table S2.

We established a two-step approach to centromere-replacement (Figure 2A and materials and methods in outline, and supplementary data in detail). The ϕC31 integrase was used to place a candidate sequence or an empty vector adjacent to the native centromere of chromosome 2 of CBS 2777, and Bxb1 integrase was subsequently used to delete the native centromere. When centromere central core DNA was used as a candidate sequence, cells that had deleted the native centromere were recovered according to the length of the candidate DNA (Figure 2B, Supplementary Table S3). Full-length (9.46 kb) central core DNA led to recovery with at least 100% efficiency whereas central core sequences less than 3.58 kb showed ∼0.1% recovery, the same as an empty integration vector (see supplementary data for calculation of efficiency). This low level of recovery was consistent with the rates of neocentromere formation that have been observed by others (34) and, as we show below, such deleted cells do, indeed, contain neo-centromeres. For the shortest length of centromere sequence (∼4.2 kb) that showed easily detectable centromere activity, we compared the activities of sequences derived from either the left or right sides of the native centromere and found no statistically significant difference (Figure 2B).

It is striking that the efficiency of recovery of cells after centromere replacement with active sequences can exceed 100% (Figure 2B). We attribute this unexpectedly high figure to the fact that cells with centromeres that are longer than the native centromere before the deletion of the native centromere grow more slowly than the deleted cells with smaller centromeres. Despite this mechanistic uncertainty, the three orders of magnitude dynamic range in the efficiency of recovery of cells that had successfully deleted the native centromere meant that the recovery of such cells could be used empirically to measure the abilities of different candidate sequences to support centromere function.

The ability of the central core DNA to substitute for the native centromere was conditional upon it being placed adjacent to the native centromere. A full-length (9.46kb) stretch of central core DNA showed no activity when placed either in the middle of the long arm of chromosome 2 (at breakpoint 1, the site of one of the translocations that gave rise to the CBS 2777 karyotype (39)) or at the neo-centromeric region of chromosome 2 (Figure 2B, Supplementary Table S3). This observation is open to two interpretations; either the candidate sequence needs to acquire the epigenetic mark present on the native centromere if it is to be able to function but it cannot do so if it is several megabase pairs distant from the native centromere, or a sequence can function when placed at a distal location but any dicentric chromosomes that were seeded were structurally unstable and were not recovered. We address this mechanistic uncertainty in later experiments.

To test whether longer regions are required purely because of their length or because they contain non-redundant elements, we tested concatamers of five or seven copies of an 889 bp sub-section of the central core. This particular subsection was chosen because it includes a prominent site of CENP-A^Cnp1^ binding (49). These concatamers were as functional as intact central core DNA of similar length (Figure 2B, Supplementary Table S3), suggesting that the functionality of longer pieces is not due to complementary unrelated activities, but to the repetition of features that are present in shorter segments. These observations are all consistent with the results of plasmid-based experiments (50) and suggest that the two assays are measuring similar aspects of centromere function.

Overall, we concluded that we could use the centromere replacement assay to investigate the specificity of centromeric DNA function. We discuss these experiments below but the results are shown in Figure 2B to allow comparison with those of the native central core DNA sequences.

### The ability of a sequence to function as a centromere is contingent upon CENP-A^Cnp1^ binding

**Figure 3.**
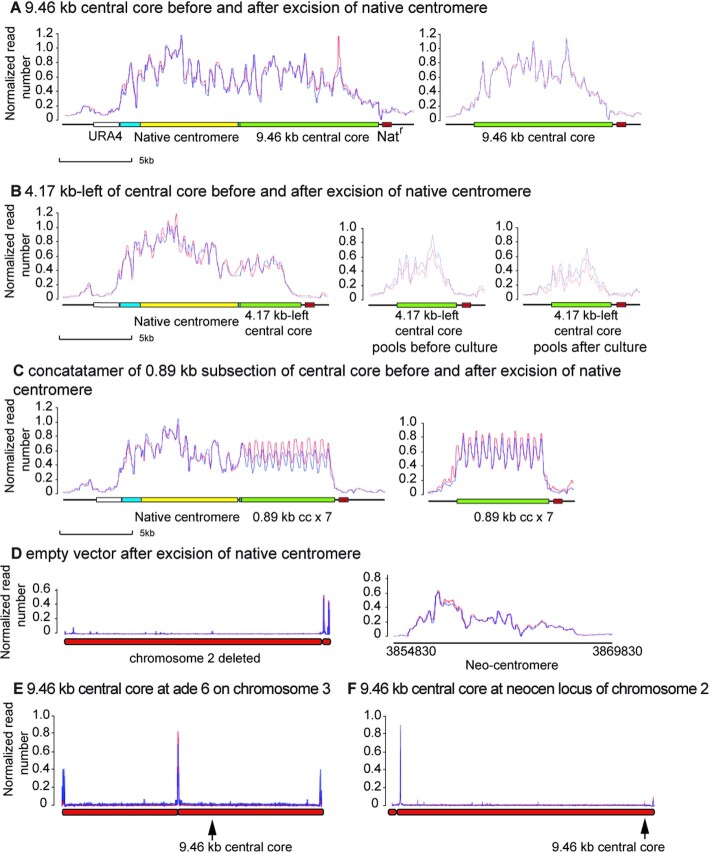
CENP-A^Cnp1^ binding to centromere and neo-centromere sequences before and after deletion of the native centromere of chromosome 2 of *S. pombe* CBS 2777. (**A**) Binding of CENP-A^Cnp1^ to an intact 9.46 kb centromere central core placed adjacent to the native centromere of CBS 2777 chromosome 2 before (left) and after (right) deletion of native centromere. A single clone prior to deletion was analysed in two independent ChIP experiments and two independent clones from a single deletion experiment were analysed. The positions of the marker genes used throughout the work; ura4 and the nourseothricin resistance gene are indicated in this panel and displayed in the same colour throughout. (**B**) Binding of CENP-A^Cnp1^ to 4.17 kb of centromere central core sequence placed adjacent to the native centromere of CBS 2777 chromosome 2 before (left panel) and after (two right panels) deletion of the native centromere. A single clone prior to deletion was analysed in two independent ChIP experiments, and two pools of twenty independent clones from a single deletion experiment were analysed before and after long term culture and the results are displayed in the right two panels. (**C**) Binding of CENP-A^Cnp1^ to a concatamer of seven copies of an 889 bp section of centromere central core sequence placed adjacent to the native centromere of CBS 2777 chromosome 2; before (left panel) and after (right panel) deletion of the native centromere. A single clone with the structure prior to deletion was analysed in two independent ChIP experiments and two pools of twenty independent clones from a single deletion experiment were analysed in the experiment shown in the right panel. (**D**) Binding of CENP-A^Cnp1^ to chromosome 2 following the integration of an empty vector adjacent to the native centromere and deletion of the native centromere. A single deletion clone was analysed in two independent ChIP experiments The left panel shows binding across the entire chromosome; the right panel shows binding to the right sub-telomeric region. (**E**) Binding of CENP-A^Cnp1^ to chromosome 3 of CBS 2777 following placement of an intact 9.5kb centromere at the ade6 locus on the right arm. (**F**) Binding of CENP-A^Cnp1^ to chromosome 2 of CBS 2777 following placement of an intact 9.5 kb centromere at the neo-centromere locus 70kb from the right telomere.

The experimental centromere sequences used in the experiments described above were derived from the laboratory strain of *S. pombe* and therefore differed from the CBS 2777 strain centromere 2 sequences by indels and SNPs (38,51). They had also been marked by the introduction of 4bp deletions of random sequences approximately every 1kb by mutagenic PCR. Overall, the experimental sequences were 94.9% identical to the centromeric DNA of chromosome 2 of CBS 2777 and 98.9% identical to the centromeric central core of chromosome II of the laboratory strain. These differences allowed discrimination between the experimental sequences and the native CBS 2777 centromeric DNA and enabled ChIP-seq to measure the binding of centromere proteins. When 9.46 kb of central core was placed adjacent to the centromere then the experimental sequences bound CENP-A^Cnp1^ before replacement, and this pattern was retained after the deletion of the native centromere (Figure 3A, for binding of CENP-A^Cnp1^ to the unmodified centromere see supplementary data, Figure 9A). The ChIP-seq data were normalized to the average of the number of reads from the centromeres of chromosomes 1 and 3, and the normalized read depth showed an overall increase in the total amount of CENP-A^Cnp1^ bound in the presence of functional test sequences (Figure 3A and B) rather than a redistribution of a fixed amount of the CENP-A^Cnp1^ bound before the integration of the test sequence. A similar pattern of CENP-A^Cnp1^ binding before and after deletion of the native centromere was also seen with a 6.8kb sub-section of the experimental centromere (Supplementary Figure 9B). The two shortest of the central core sequences to show clone recovery above background in the centromere-replacement assay were 4.17 kb and 3.59 kb. We analysed CENP-A^Cnp1^ binding (Figure 3B and Supplementary Figure S9C) to one clone containing the 4.17kb sequence before, and a pool of twenty clones after deletion of the native centromere and showed CENP-A^Cnp1^ binding to the 4.17kb sequence both before and after. We concluded that centromeric DNA of this length was functional. In contrast, the replacement clones containing the shorter 3.59 kb centromere sequence grew slowly and we were initially unable to derive ChIP-seq data. After several months of storage with two rounds of re-streaking to ensure viability, the clones had recovered growth and showed CENP-A^Cnp1^ binding exclusively to sub-telomeric DNA. This sequence, as we discuss below, was also bound by CENP-A^Cnp1^ after deletion of the native centromere 2 from clones that contained an empty vector placed adjacent to the native centromere 2. These results demonstrate that centromeric sequences of 3.59kb are unable to support the stable presence of a native centromere.

The concatamers of five or seven copies of an 889 bp sub-section of the central core bound CENP-A^Cnp1^ both before and after the deletion of the native centromere (Figure 3C for the seven copy array and Supplementary Figure S9D for the five copy array) consistent with their functionality in the replacement assay. In both cases, the regular pattern of binding probably reflects the random assignment of short Illumina reads to the units of the array rather than any underlying uniformity of binding.

In clones that were recovered after an empty vector sequence had been placed adjacent to the native centromere before deletion, all of the CENP-A^Cnp1^ was located on distal sub-telomeric DNA (Figure 3D) and mainly on a 13 kb stretch of DNA ∼70 kb from the telomere. In total, we analysed six centromere-deleted clones derived from the empty vector or from experimental sequences less than 3.58 kb that showed no detectable activity (2.0 kb and 1 kb candidates) for CENP-A^Cnp1^ binding, and all showed binding to the same distal sub-telomeric stretch of DNA. Similar results were obtained by Ishii and colleagues (34,52) in experiments that used the laboratory strain of *S. pombe*. Ishii and colleagues characterized the properties of these CENP-A^Cnp1^ sequences in detail and identified them as the equivalents of the neo-centromeres seen in metazoan organisms and we refer to them as such in the discussion below.

To test whether the centromeres with the 4.17 kb stretch of central core DNA were stable, we used an approach analogous to that used in mutation accumulation experiments (53,54). We cultured 20 clones on solid agar with ten rounds of re-streaking picking a random colony at each round. Picking a random colony at each round of streaking minimises the effect of selection upon the measurement of the rate of appearance of clones containing neo-centromeres. There was no major difference between CENP-A^Cnp1^ binding before and after culture (Figure 3B) and, in particular, no detectable neo-centromere formation (Supplementary Figure S9C). Given a colony size of 5 × 10^6^ at each round of streaking, one can calculate an upper bound to *P* the probability, per cell division, of centromere movement from the 4.17 kb sequence to the neo-centromere of less than 0.1% per cell division (with 99% confidence). For a more detailed explanation of the calculation and the use of the mutation accumulation approach see section 1.5 of the supplementary data.

To analyze whether CENP-A^Cnp1^ binding to the test sequences was contingent upon being adjacent to the native centromere, we analysed CENP-A^Cnp1^ binding to a 9.46 kb stretch of centromeric DNA that we had placed at the ade6 locus on chromosome 3. None was observed (Figure 3E). In light of the data showing neo-centromere formation in the subtelomeric region we then asked whether centromeric DNA could bind to CENP-A^Cnp1^ when placed at the neo-centromere locus on a chromosome with a centromere at the native locus. ChIP-seq for CENP-A^Cnp1^ showed that such an ectopically placed sequence also did not bind detectable CENP-A^Cnp1^ (Figure 3F). These observations are consistent with the lack of activity of such modified chromosomes in the centromere replacement assay (Figure 2B).

In summary, these analyses (Figure 3) demonstrated that CENP-A^Cnp1^ binding to a candidate sequence is contingent upon a juxta-centromeric location. Moreover the ability of a sequence to function in the replacement assay (Figure 2B, Supplementary Table S3) correlated with its ability to bind CENP-A^Cnp1^ and required that the sequence be greater than about 4.2 kb in length.

### Using centromere replacement in CBS 2777 to analyse sequence requirements for centromeric DNA function

**Figure 4.**
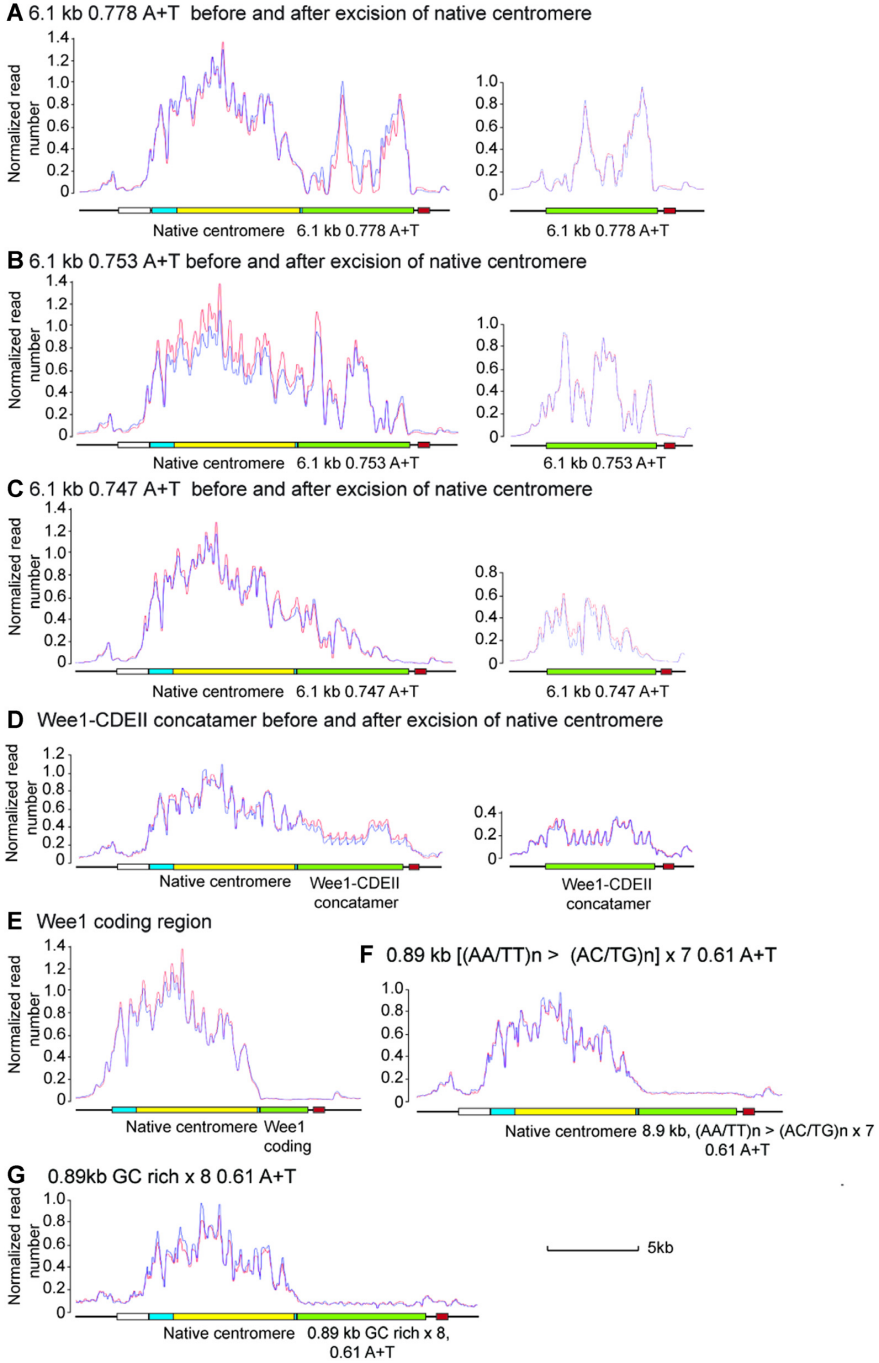
CENP-A^Cnp1^ binding to candidate sequences before and after centromere-replacement in *S. pombe* CBS 2777. (**A**) Binding of CENP-A^Cnp1^ to a 0.778 A + T sequence derived from *C. sporogenes* adjacent to the native centromere of CBS 2777 chromosome 2 before and after replacement. On the left-hand side, two independent clones before the replacement were analysed. On the right-hand side two pools of twelve independent clones from a single replacement experiment were analysed (also in B and C). The red and blue lines in these and subsequent panels represent the results of duplicate ChIP∼seq experiments. (**B**) Binding of CENP-A^Cnp1^ to a 0.753 A + T sequence derived from *C. sporogenes* adjacent to the native centromere of CBS 2777 chromosome 2 before and after replacement. (**C**) Binding of CENP-A^Cnp1^ to a 0.747 A + T sequence derived from *C. sporogenes* adjacent to the native centromere of CBS 2777 chromosome 2 before and after replacement. (**D**) Binding of CENP-A^Cnp1^ to a concatamer of short stretches of DNA derived from the *S.pombe* wee1 gene and segments derived from the CDEII elements of various *S. cerevisiae* centromeres before and after replacement. See Supplementary Figure S8 for details of the sequence. The regular nature of the profile reflects the fact that the mapping of the ChIP reads to the sequence assigns the reads randomly to the target concatamer because all of the units are identical. (**E**) Binding of CENP-A^Cnp1^ to the *S. pombe* wee1 gene placed adjacent to the native centromere of CBS 2777 chromosome 2. (**F**) Binding of CENP-A^Cnp1^ to a seven-unit concatamer (6.23 kb) of a mutant version of the 0.89kb sequence analysed in Figure 3C that had been placed adjacent to the native centromere of CBS 2777 chromosome 2. The mutations in the units of this concatamer are such that all A or T homopolymer arrays longer than 2bp are disrupted by G or C mutations (Supplementary data 2.2 for details). (**G**) Binding of CENP-A^Cnp1^ to an eight-unit concatamer (6.23 kb) of a mutant version of the 0.89kb sequence analysed in Figure 3C that had been placed adjacent to the native centromere of CBS 2777 chromosome 2. The mutations in the units of this concatamer are such that A or T homopolymer arrays longer than 2 bp are retained but the A + T content is reduced by mutations outside the runs (Supplementary data 2.2 for details).

The centromere replacement assay potentially enables the identification of those features of centromeric DNA sequences that are are important for functionality. In many species, centromere sequences are A + T rich compared to the rest of the genome and so the obvious question was whether any A + T rich DNA would function as a centromere in *S. pombe*. Genomes from bacteria of the genus *Clostridia* have some of the highest A + T content of any bacteria so we tested the activities of three independent 6.1 kb stretches of A + T rich DNA from *C. sporogenes* (0.778, 0.753, 0.747 A + T) for their activities in the centromere replacement assay. All showed activity that was indistinguishable from that of native *S. pombe* centromere central core sequences (Figure 2B, Supplementary Table S3). Similarly, two shorter stretches of *C. sporogenes* DNA that were 0.79 A + T and were 4.2 and 3.58 kb in length showed activities that were indistinguishable from those of similar lengths of native *S. pombe* centromeric DNA. The *C. sporogenes* A + T rich sequences bound CENP-A^Cnp1^ at high levels (Figure 4A–C) consistent with their activities in the replacement assay. These measurements suggest that A + T rich DNA of any sequence is sufficient for centromere function as judged by the replacement assays (Figure 2B). To establish the threshold of A + T content necessary for centromere function we also tested a 5.1kb stretch of bacterial *C. acetobutylicum* DNA, which contained 0.71 A + T (0.67–0.73 per kb). The sequence showed weak but detectable activity (Figure 2B, Supplementary Table S3) and bound CENP-A^Cnp1^ both before and after the replacement (Supplementary data, Figure 9F). We wanted to know whether the centromeres formed on *C. acetobutylicum* DNA were stable and so we streaked out two of the replacement clones to single colonies and then analysed these in turn by ChIP∼seq. In these clones, all of the reads mapping to chromosome 2 mapped to the neo-centromere region (not shown), and we therefore concluded that the centromeres formed on 0.71 A + T *C. acetobutylicum* DNA were unstable and that by streaking we had selected for faster-growing cells containing neo-centromeres. Thus the 5.1kb stretch of *C. acetobutylicum* DNA showed lower levels of activity than the 6.1 kb stretches of *C. sporogenes* DNA that were greater than 0.747 A + T which suggested that the threshold for centromere activity lies between 0.71 and 0.747 A + T. Consistently, a 5.7 kb unit repeat of human Y chromosome alphoid DNA that was 0.63 A + T showed less than 1% of the activity of an equivalent length of *S. pombe* centromere DNA and did not bind CENP-A^Cnp1^ (Figure 2B, Supplementary Figure S9G).

We wanted to provide additional evidence that an arbitrarily chosen A + T rich sequence could function as a centromere in *S. pombe* and so we concatemerized an array of *S. cerevisiae* centromeric CDEII segments (each ∼20 bp in length and with ∼0.94 A + T content) interspersed with 20 bp segments of the *S. pombe* wee1 gene (0.55 A + T content) to generate a 5.9 kb sequence that was 0.74 A + T overall. This concatamerized sequence also functioned as well as native *S. pombe* centromeric DNA by the criteria of activity in the replacement assay (Figure 2B) and CENP-A^Cnp1^ binding (Figure 4D). Wee1 itself showed no binding of CENP-A^Cnp1^ (Figure 4E) thus establishing that short segments of A + T rich DNA are important for centromere function.

Having shown that a non-centromeric DNA sequence that was greater than 0.74 A + T could function as a centromere in our assay the next question was whether the relatively high A + T content characteristic of *S. pombe* centromeric DNA was necessary for centromere function. To address this question we mutated the 889 bp sequence that we had shown to be active as a tandem array of five or seven copies (Figure 2B). We either disrupted the A/T runs in the sequence with alternate Cs or Gs or introduced Cs or Gs outside the A/T runs (supplementary data section 2.2). Both approaches reduced the A + T content from approximately 0.7 to 0.61 and destroyed the centromere activity of the sequence as well as its ability to bind CENP-A^Cnp1^ (Figure 4F and G). Hence, A/T runs are insufficient and an overall high A + T content is required.

In *S.cerevisiae* the centromeric nucleosomes form around A + T rich DNA (55) and so it seemed possible that an explanation for the ability of random pieces of A + T rich DNA to form a centromere in *S. pombe* was that the A + T rich DNA was acting to nucleate the formation of centromeric nucleosomes. To test this idea, we conducted a linear regression between the A + T content of 180bp segments of the A + T rich sequences, the laboratory strain central core, the 0.89kb subsection of the central core and the respective levels of CENP-A^Cnp1^ binding (Supplementary Table S4). No association could be detected in the relationship between these two variables in three of the sequences and, in the case of the 0.778 and 0.753 A + T *C. sporogenes* sequences and the 0.89 kb subsection of the central core, there was a significantly negative relationship. For the 0.89 kb subsection of the central core, the associated *R*^2^ value was 0.66 but was <0.21 in the case of the two other sequences. Taken together these results argue against the idea that a tendency for centromeric nucleosomes to form around A + T rich DNA is responsible for the ability of A + T rich DNA to function as a centromere.

### The neo-centromere and the native centromere are epigenetically distinct

**Figure 5.**
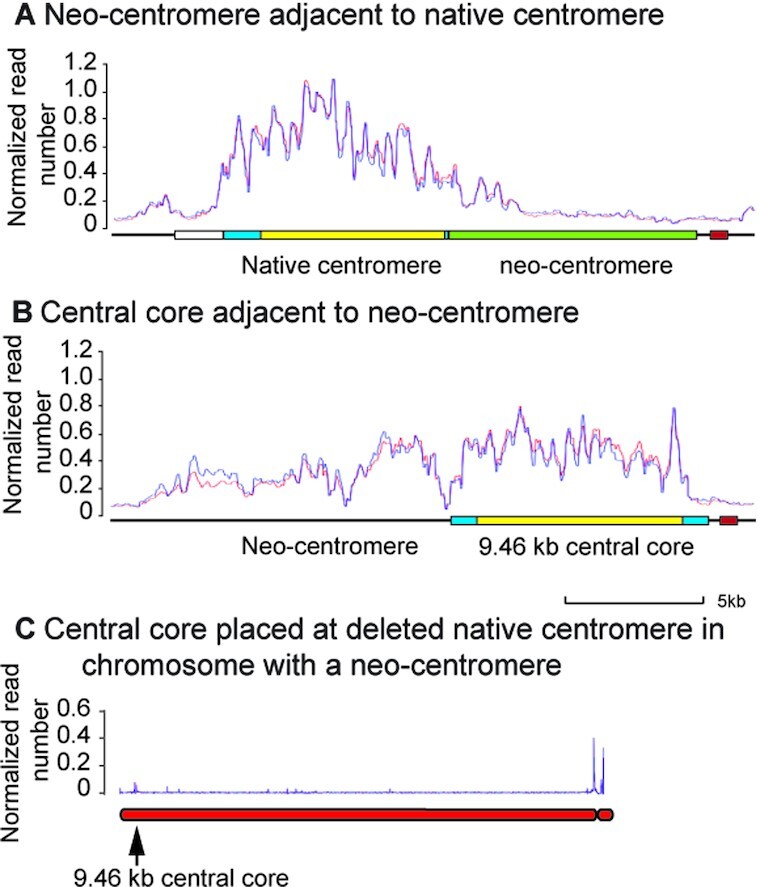
CENP-A^Cnp1^ binding to candidate sequences before and after centromere-replacement in *S. pombe* CBS 2777. (**A**) Binding of CENP-A^Cnp1^ to a 9kb stretch of neo-centromeric DNA that had been placed adjacent to the native centromere of CBS 2777 chromosome 2. (**B**) Binding of CENP-A^Cnp1^ to the neo-centromeric region of chromosome 2 of CBS 2777 after deletion of the native centromere and integration of a 9.46 kb stretch of central core DNA at the neo-centromere. (**C**) Binding of CENP-A^Cnp1^ to a version of chromosome 2 that had formed a neo-centromere after deletion of the native centromere and which had then been modified by re-integration of a 9.46 kb stretch of central core DNA at the locus of the native centromere thus reconstructing the original, unmodified DNA structure of the chromosome.

If neo-centromeres and native centromeres were sequences that were activated by the same epigenetic mark then one would expect a neo-centromere sequence to bind CENP-A^Cnp1^ with the same pattern when placed adjacent to the native centromere as it does when it functions as a neo-centromere. We tested this prediction by placing the neo-centromere sequence from the tip of the long arm of chromosome 2 adjacent to the native centromere. However, upon doing so, CENP-A^Cnp1^ binding extended only about 1kb into the neo-centromeric DNA (Figure 5A) rather than throughout the sequence as seen at the neo-centromere itself. Consistently when analysed in the centromere-replacement assay, it showed less than 1% of the activity of an equivalent length of *S. pombe* centromeric DNA (Figure 2B, Supplementary Table S3). These results, as we discuss in more detail below, suggest that that neo-centromeres and native centromeres are either established or maintained by distinct epigenetic processes (52). To investigate the nature of the epigenetic marks at the native and neo-centromere in more detail we carried out a reciprocal analysis; we placed 9.46 kb of central core DNA next to the neo-centromere. This introduced sequence showed strong binding of CENP-A^Cnp1^ (Figure 5B) and increased the binding of CENP-A^Cnp1^ to the neo-centromeric DNA. We wanted to confirm that the CENP-A^Cnp1^ binding to the native centromeric DNA was contingent upon the adjacent neo-centromere and to do this we placed 9.46 kb of central core DNA at the locus of the deleted native centromere; no binding was observed (Figure 5C). The observation of the stability of the epigenetic mark at the neo-centromere in the presence of native centromeric DNA is consistent with observations made on human neo-centromeres which demonstrated that neo-centromeres are stable in the presence of centromeric alphoid DNA in *cis* (56). Together, these results indicate that neo-centromeres require an additional activity compared to native centromeric or A + T rich DNA sequences to make them competent for centromere formation.

### A + T rich bacterial DNA functions as a centromere in the laboratory strain of *S. pombe*

The previous experiments demonstrated that we could engineer centromeres with specific structures in the *S. pombe* strain CBS 2777. This had enabled us to make two discoveries; first that A + T rich bacterial DNA or A + T rich synthetic DNA was capable of functioning as a centromere and second that neo-centromeric DNA was different from native centromeric DNA in so far as it showed no activity in the centromere replacement assay and failed to bind CENP-A^Cnp1^ when placed adjacent to the native centromere. The CBS 2777 karyotype is extensively re-arranged and we wanted to confirm our discoveries in the well-characterized laboratory strain.

**Figure 6.**
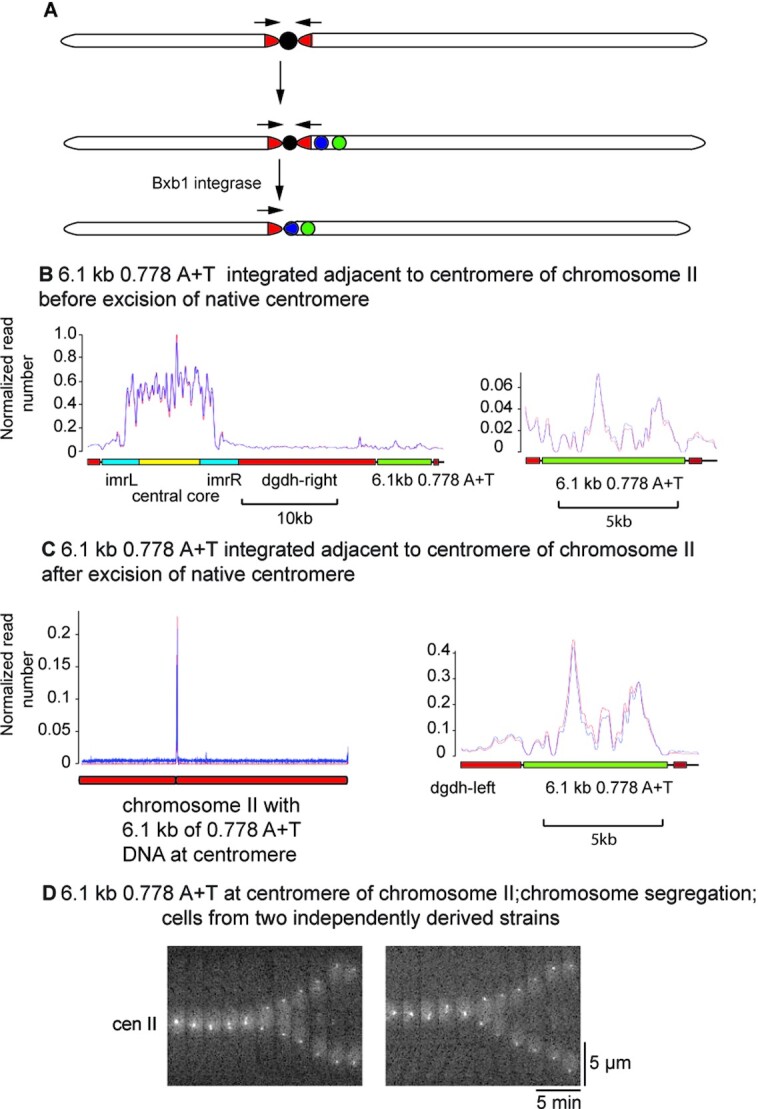
Centromere replacement in chromosome II of the laboratory strain of S. pombe and CENP-A^Cnp1^ binding to candidate sequences before and after centromere-replacement. (**A**) Outline of centromere replacement in the laboratory strain of *S. pombe*. For details see the main text and figure 10, 11 and 12 of supplementary data. (**B**) Binding of CENP-A^Cnp1^ to the centromere and flanking sequences on a chromosome II in which 6.1 kb of a 0.778 A + T sequence derived from *C. sporogenes* was placed adjacent to the native centromere of the laboratory strain chromosome II before deletion of the native central core and right-hand array of dgdh repeats. The left-hand panel shows the central core, right-hand dgdh array and the inserted sequence, the right-hand panel shows a map of CENP-A^Cnp1^ binding to the 6.1 kb 0.778 A + T *C. sporogenes* sequence, re-scaled to indicate the amount bound. (**C**) Binding of CENP-A^Cnp1^ to chromosome II (left) and to the 6.1kb 0.778 A + T *C. sporogenes* sequence after deletion of the native central core and right-hand array of dgdh repeats from the chromosome, as described in A. (**D**) Kymographs showing accurate segregation of chromosome II in two independent laboratory strains with 6.1 kb 0.778 A + T *C. sporogenes* DNA functioning as a centromere. Chromosome II is marked by the lacO array / lacI-GFP system. Although interphase cells with two copies of chromosome II were occasionally observed, all 118 and 115 cells in the two strains which underwent mitosis segregated chromosome II correctly as shown.

We chose chromosome II as the substrate for engineering centromere replacement in the laboratory strain. First, we engineered a lacO array into the centromere proximal region enabling visualization by binding of a lac repressor (lacI) GFP fusion protein (see Supplementary Figures S10 and S11). We then placed the 6.1kb stretch of *C. sporogenes* DNA together with an *attB* site for the Bxb1 integrase ∼100 bp outside the right-hand edge of the palindromic centromeric heterochromatin array using site-specific integration with ϕC31 integrase. We then targeted a Bxb1 integrase *attP* site into the IMR sequence on the left hand of the central core and finally used the Bxb1 integrase to delete the central core of the native centromere and the right-hand heterochromatin array (Figure 6A). This generated a chromosome lacking the native centromere central core of chromosome II but with a candidate centromere containing the 6.1kb stretch of 0.778 A + T *C. sporogenes* DNA flanked on its left-hand side by the native heterochromatin array (Figure 6A and Supplementary Figure S10). We measured the binding of CENP-A^Cnp1^ to the 6.1kb stretch of 0.778 A + T *C. sporogenes* DNA both before and after deletion of the native centromere central core (Figure 6B, C). This showed binding of CENP-A^Cnp1^ to the bacterial DNA at both steps in the experiment and an increase in the amount of binding upon deletion of the native centromere. Thus CENP-A^Cnp1^ binding to functional sequences did not require the candidate DNA to be directly adjacent to the native centromeric DNA. However, given that CENP-A^Cnp1^ did not bind to the 9.46 kb central core sequence when it was placed at the ade6 locus on chromosome III (see Figure 3E) the binding of CENP-A^Cnp1^ to the 6.1kb stretch of 0.778 A + T *C. sporogenes* DNA seen before deletion of the native centromere is probably a consequence of the fact that the native centromere and the test sequence are much closer to one another in the experiment shown in Figure 6B than in the earlier controls. We measured the segregation accuracy of the GFP labelled chromosome II after deletion of the native centromere central core using live-cell imaging (Figure 6D). This showed no detectable segregation errors in 233 mitoses allowing us to put an upper bound on the rate of segregation errors of 0.0196 (with 99% confidence) thus establishing that A + T rich bacterial DNA supports accurate chromosome segregation.

These manipulations established that we could assay the ability of a sequence to function as a centromere in the laboratory strain by placing the sequence next to the centromere and determining whether it bound CENP-A^Cnp1^. We therefore also placed the neo-centromeric DNA next to the centromere of the laboratory strain chromosome II and measured CENP-A^Cnp1^ binding. None was observed (Supplementary Figure S9H) consistent with the lack of centromere activity of this sequence in CBS 2777.

### Sequences of 43 Opisthokont centromeres support a ‘mutation-selection balance’ model for the evolution of centromeric DNA in these species

**Figure 7.**
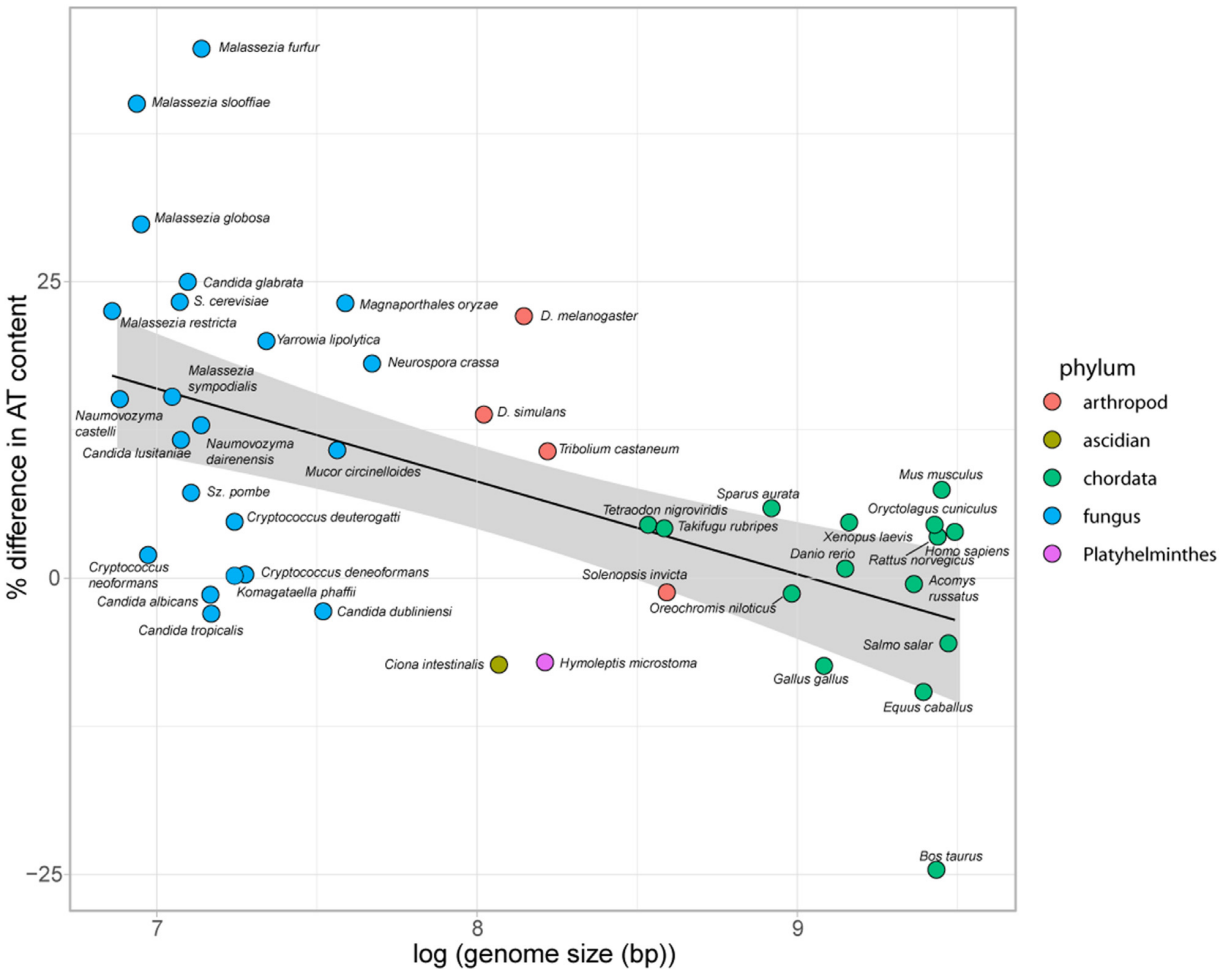
The difference between the A + T content of the centromeric DNA and that of the genome as a function of genome size for 43 fungi, yeast and animals (Opisthokonts). The figure is based upon data in table 5 of the supplementary data. We carried out a linear regression of the relationship between genome size as the independent variable and the AT content of the centromeric DNA with respect to the genome average as the dependent variable using R. The regression coefficient was calculated as −7.831 with a *P*-value of 9.14 × 10^–5^. The adjusted *R*^2^ value or the proportion of the variance of the dependent variable (relative centromeric AT content) accounted for by the independent variable (genome size) was 0.2979. The *F*-statistic was 18.82 on 1 and 41 degrees of freedom.

The observation that both randomly chosen bacterial and engineered A + T rich DNA sequences are capable of functioning as a centromere in *S. pombe* would seem to contradict the large body of work that demonstrates sequence and species specificity for the function of native centromeric DNA in both *S. pombe* (50) and metazoan organisms (17, 30). However, we suggest that the two types of results may be reconciled by a ‘mutation-selection balance’ model for the evolution of centromeric DNA. In this model, selection favours centromeres that are A + T rich relative to the genome but a variety of mutational processes; including those arising from the unstable chemistry of the DNA molecule itself, invasion by transposable elements and centromere turnover as a result of neo-centromere formation tend to drive the centromeric DNA to a base sequence content that resembles that of the genome as a whole. Many such mutations will be lethal but a proportion may be partially compensated for by mutations in kinetochore proteins that contact the DNA giving rise to species-specific centromeric DNA that has an A + T content that is less than optimal. A conceptually similar model is widely accepted (57) as an explanation of codon-usage bias (58–60). Key to the development of the ‘mutation-selection-balance’ model for codon-usage bias was the observation that codon usage bias was strongest in organisms with large effective population sizes such as bacteria and *Drosophila* (59). The principles of why this is so are well understood in terms of population genetic theory (61). If a ‘mutation-selection balance’ model applies to the evolution of centromeric DNA across the evolutionary spectrum then one would predict that the extent of the difference between the A + T content of the centromere and the genome should be an increasing function of the effective population size of the species in question. The effective population size has yet to be determined for many organisms and so we investigated the relationship using genome size as a surrogate because genome size is inversely related to effective population size (40). Accordingly, we carried out a linear regression for 43 animal, yeast and fungal species using the logarithm of the genome size as the independent variable and the difference between the A + T content of the centromere and the genome as the dependent variable. The results (Figure 7) are consistent with the mutation-selection balance model, suggesting that A + T rich DNA will function as a centromere in most if not all Opisthokont species. *R*^2^, or the proportion of the variance of the dependent variable accounted for by the independent variable, is only 0.2979, though; reflecting the fact that many species have a centromeric DNA sequence content that differs from that predicted by the model. We discuss the reasons for this below. Moreover, although the analysis suggests that the ‘mutation-selection balance’ model holds for most of the 43 species considered, it is not necessarily the case that it holds for all and some exceptional species may have distinct sequence specificities for centromere recognition. We wanted to know whether these conclusions could be extended beyond the Opisthokonta and so we carried out similar analyses for six species (*Oryza sativa, Zea mays, Glycine max, Daucus carota, Chlamydomonas reinhardtii, Phaeodactylum tricornutum*) from the Chloroplastida (green algae and land plants) but could not exclude the null hypothesis of no significant relationship between genome size and the relative centromeric AT content. The inability to reach any conclusion may reflect either the paucity of data available for Chloroplastida or a difference in the mechanisms of centromere specification between Chloroplastida and Opisthokonta.

## DISCUSSION

Here we report the discovery that A + T rich DNA; whether of bacterial origin or engineered, is capable of functioning as a centromere in the model organism *Schizosaccharomyces pombe*. We propose that this is also true for many other eukaryotes of the Opisthokonta lineage to which yeast, fungi and animals belong (62). Two facts support this claim. First, a large body of work, particularly that of Nurse and colleagues (63), has shown that what is true of *S. pombe* is also often true of vertebrates including humans. Secondly and more directly, we have shown that the extent of the A + T bias of centromeric DNA relative to the genome average scales with the inverse of the genome size for yeast, fungi and animals. This is consistent with the idea that the sequence content of centromeric DNA is maintained by a balance between mutation and selection, with selection acting to increase A + T content of the centromeric DNA and mutation, in its broadest sense, acting to drive the sequence towards the genome average. The observation that expression of the centromere-specific H3 from *Saccharomyces cerevisiae*; Cse4p, can functionally complement the loss of CENP-A in human cells (64) is also consistent with the idea that, at least in Opisthokonta, there are deeply conserved features of centromeric DNA. Thus, we need to discuss the following questions; what is the feature of A + T rich DNA that enables it to function as a centromere, how can our claims be tested, are there explanations for the relationship between genome size and centromeric A + T content other than a ‘mutation-selection balance’ model and to what extent do our conclusions extend beyond the Opisthokonta?

Firstly, although we do not know why A + T rich or native centromeric DNA is necessary for centromere formation we have shown that these sequences are not sufficient in isolation but need to be placed close to a pre-existing centromere to acquire the epigenetic mark that enables CENP-A^Cnp1^ binding and centromere function. Thus, A + T rich DNA can be regarded as permissive for the propagation of the epigenetic mark. How A + T rich DNA allows propagation of the mark is not clear. We tested the idea that the centromere-specific nucleosome forms most readily around A + T rich DNA by carrying out a regression analysis of the relationship between A + T content and the amount of bound CENP-A^Cnp1^ for six different sequences. The results (Supplementary Table S4) were inconclusive. Alternatively, the requirement for A + T rich DNA may reflect the optimal sequence for the remodelling of the chromatin necessary for assembly of the kinetochore. The fact that a species such as *Mucor circinelloides* (65) that lacks CENP-A has A + T rich centromeres is consistent with the idea that a preference for A + T rich DNA is not determined by CENP-A containing nucleosomes. A or T homopolymer tracts are known to antagonize nucleosome formation (66) and it may be that, as suggested by Shukla and colleagues (67), destabilisation of nucleosomes promotes the formation of centromeric chromatin. In budding yeast A + T rich DNA has been shown to promote chromatin remodelling by the RSC complex (68) and it seems possible that similar activities are at work at the centromere in *S .pombe*. Testing these ideas requires determination of the mechanistic basis of the ability of A + T rich DNA to function as a centromere. Genetic analysis will be central to this task. Analysis of the functionality of candidate centromeres that have A + T contents in the critical region between 0.71 and 0.747 A + T may enable the identification of variants in key genes that confer different base sequence preferences. Such variants would cast light on the mechanistic basis of the sequence requirements for centromere function. The demonstration that neo-centromeric DNA is not inherently centromeric when placed at the native centromere might also allow a powerful approach to resolving the mechanistic basis of the requirement for A + T rich DNA. Thus one experimental test for the role of chromatin remodelling in conferring centromere function would be to increase the activity of a potential centromeric chromatin remodeller on the neo-centromeric DNA when it has been placed adjacent to a native centromere.

Regardless of mechanistic understanding, the claim that A + T rich DNA will function as a centromere in most, if not all, eukaryotes needs to be tested. The approach that we have used combines the activities of two well-characterized serine recombinases; the integrases of the *Streptomyces* phage ϕC31 and the mycobacteriophage Bxb1. These are two of the most efficient of the large serine integrases (35,36) and so our strategy is likely to be broadly applicable. There may however be other simpler ways of testing the claim. Centromeric DNA transfected into human fibroblasts forms autonomously segregating structures that have been termed artificial chromosomes (17,30). It would now be of interest to analyse the structures formed following transfection of *Plasmodium falciparum* genomic DNA (80% A + T) and other A + T rich sequences into human fibroblasts.

We argued that the observation that the relative A + T content of the centromeric DNA scales with effective population size supports the idea that A + T rich DNA will function as a centromere in most Opisthokonta species. This argument is indirect and one needs to consider other explanations for the relationship. In particular, it has been suggested that the low GC content of centromeric DNA reflects the low rates of recombination in centromeres and a GC bias of mismatch repair (27). Although mismatch repair is GC biased in some organisms, it is not generally so (69), and thus does not explain the relationship that we report. Although the many outliers from the regression line in Figure 7 may call into question the merit of the approach, we suggest, rather, that these highlight species that deserve further study and thus emphasize its power. Four examples illustrate this point. Firstly, the sequences from the *Ciona intestinalis* genome, which represent an outlier towards low A + T content, were included because they were the major tandem repeats of the respective chromosomes (Y. Satou, personal communication) but they have not been shown to bind CENP-A. Given the fact that they are much less A + T rich than one would expect, a study of the identity of the centromeres in *Ciona intestinalis* would be worthwhile. Second, the low A + T content of the sub-telomeric 179bp repeats of *Hymenolepis microstoma* (70) that are almost certainly centromeric, may reflect the high levels of GC biased mismatch repair that arise from a sub-telomeric, recombinationally active location. Third, the presence of relatively G + C rich centromeres in the yeast CTG clade including *Candida* sp. suggests that these species may have a distinct and potentially informative mechanism of centromere recognition and thus would also be worth experimental attention. Fourth, some of the variance between the predicted and the observed value of centromeric A + T content may also be accounted for by the complexity of the mutational processes to which the centromere is subject. For example, *Drosophila simulans* has a similar effective population size to *Drosophila melanogaster* (71) but has centromeric DNA that is relatively G + C rich. This could be due to more active transposon activity in *D. simulans* than in *D. melanogaster* or reflect variation in centromere sequence usage between different population isolates of both species and, as yet, incomplete sampling of the possible centromere sequences. Again, this explanation can be tested.

If relatively A + T rich DNA does indeed function as centromeric DNA in most eukaryotes then this would suggest a solution to the ‘centromere paradox’ that would be applicable beyond organisms with asymmetric meioses. The rapid evolution of centromeric DNA would be possible because variation that increased overall A + T content would not be constrained by selection and thus the sequence would be free to drift from one selective peak to another at a rate determined only by the rates of mutation from G or C residues to A or T residues. This idea could be tested by comparing the spectra of variants of centromeric and non-centromeric DNA in large population samples.

The failure of a neo-centromere sequence to show any activity in the centromere replacement assay or to bind CENP-A^Cnp1^ when placed adjacent to the native centromere (Figure 5A) suggests that neo-centromeres are not simply weaker versions of native centromeres and that the neo-centromere and the native centromere are qualitatively different. We explored the nature of this difference by carrying out the reciprocal experiment in which we placed native centromeric DNA adjacent to the neo-centromere (Figure 5B). When this was done CENP-A binding was observed not only on the neo-centromere but also on the native centromeric DNA. Moreover binding of CENP-A to the neo-centromere increased in the region of the neo-centromere closest to the newly introduced native centromere (compare Figures 3D and 5B). This result can be explained if we hypothesise that the formation or inheritance of centromeric chromatin on the neo-centromeric DNA is contingent upon the presence of one or more factors not present at native centromeres and that once these factors have enabled the neo-centromeric DNA to acquire centromeric identity and a fundamental centromeric epigenetic mark then centromeric chromatin can spread onto the permissive DNA of the native centromere. One can reconcile this idea of additional factors with the idea that de-stabilization of H3 nucleosomes is the defining feature of native centromeric DNA. Hence additional factors that promote neo-centromere formation may do so by destabilizing H3 nucleosomes on DNA that is neither A + T rich nor centromeric and such factors may be found at loci where neo-centromeres form. The existence of such factors would account for the inability of neo-centromeric DNA to bind CENP-A^Cnp1^ when placed at the native centromere but do not explain how neo-centromeres acquire the centromeric epigenetic mark itself.

The question as to the relationship between neo-centromeres and native centromeres bears upon the final question: to what extent do our observations and claims hold beyond the Opisthokonta. We have started to address this question by looking at centromeres in the Chloroplastida (also called Viridiplantae) but found no evidence that would allow us to reject the null hypothesis; the A + T content of centromeric DNA in the Chloroplastida did not significantly differ from that of the genome as a whole. That the small sample size of only six may account for the lack of significance emphasizes the need for more work on the centromeres of the Chloroplastida.

Our discussion of the relationship between native centromeres and neo-centromeres, and the lack of knowledge about centromeres in the Chloroplastida both reflect a fundamental uncertainty about centromeres and their degree of diversity at the individual, population and deeper evolutionary levels. Identifying and understanding the molecular basis of this diversity remains a critical task.

In summary, our chromosome engineering approach has led to an explanation of why centromeric DNA is often A + T rich and to new mechanistic ideas about the relationship between native and neo-centromeres. These discoveries were made possible by the ChIP seq and live-cell imaging that, in turn, have been enabled by direct manipulation of the native centromeric DNA.

## NOTE ADDED IN PROOFS

During the preparation of the proofs of our manuscript, we became aware of the work of Diner and colleagues (72) reporting that AT rich DNA of foreign origin is capable of centromere function in the diatom, *Phaeodactylum tricornutum*. This observation is similar to our discovery that AT rich DNA of bacterial origin is capable of functioning as a centromere in *S. pombe* and indicates that AT rich DNA will, regardless of origin, function as a centromere not only in the Opisthokonta but more widely in eukaryotes.

## DATA AVAILABILITY

Fastq, fasta and coverage vector files have been deposited at GEO accession GSE181806. For details of the correspondence between these files and the results in the main and supplementary text see Supplementary Table S8.

## Supplementary Material

gkab1219_Supplemental_Files

## Notes

Present address: Anne C. Barbosa, Laboratory of Human Genetics, Department of Genetics and Evolutionary Biology Institute of Biosciences of the University of São Paulo - USP Rua do Matão, no. 277, Cep: 05508-090, Cidade Universitária, São Paulo, SP, Brazil.

Present address: Kazhal Karari, Department of Medical Laboratory Sciences, College of Sciences, University of Raparin, Ranya, Kurdistan Region of Iraq.
