# Supplementary material for: Mutation and selection explain why many eukaryotic centromeric DNA sequences are often A + T rich
Source: Nucleic Acids Res. 2021 Dec 20;50(1):579–96. doi: 10.1093/nar/gkab1219 (PMC8754631; doi:10.1093/nar/gkab1219)
Supplement: gkab1219_Supplemental_Files [file gkab1219_supplemental_files.zip › Barbosa_et_al_Supplementary_data_reduced.pdf]

Barbosa et al., Supplementary data

Table of Contents

Supplementary data table 3. Assaying centromeric activity of centromeric and candidate DNA in the centromere replacement assay 19 3

1 Chromosome engineering of chromosome 2 of *S. pombe* CBS 2777 4

1.1 Grapical summaries of the engineering of chromosome 2 of *S. pombe* strain CBS 2777 to enable assay of the centromere activity of a candidate sequence 4

1.2 Assaying the ability of a candidate sequence to support centromere formation using site-specific recombination. 5

1.3 Constructing controls for the centromere-replacement assay in which a central core of *S. pombe* centromeric DNA is placed either at the centromere distal telomere or at breakpoint 1 on chromosome 2 of CBS 2777 8

1.4 Transformation of cells for assay of centromere activity 8

1.5 Estimating the stability of the kinetochore on a chromosome with 4.17kb of centromeric DNA. 10

2 Sequences assayed 11

2.1 Central core derived sequences 11

2.2 Candidate sequences 14

3 Chromatin immunoprecipitation (ChIP) 26

3.1 ChIP 26

3.2 Computational analysis of ChIP-seq data 26

3.3 Results of ChIP-seq analysis discussed but not illustrated in main text 27

4 Centromere replacement in the laboratory strain of *S. pombe* 29

4.1 Chromosome engineering 29

5 Linear regression of AT content of 180bp sub-sections of centromere sequences and CENP-A^{Cnp1} binding for six sequences 33

6 Evolutionary analysis of the AT content of centromeric DNA 34

References 38

List of Figures

Supplementary data figure 1. Sequence of steps used to engineer a chromosome containing a candidate sequence adjacent to the native centromere 4

Supplementary data figure 2. pFA6a-natMX6 REV ϕC31 attP attP Bxb1 attB vector used to integrate sequences into chromosomes using site-specific recombination with ϕC31integrase 4

Supplementary data figure 3. Centromere swapping using Bxb1 integrase 5

Supplementary data figure 4. Differences in colony size between cells containing an intact central core as a candidate sequence and cells containing an empty vector upon transformation with the pREP81-Bxb1 expression construct. 6

Supplementary data figure 5. Checking the sequence organization of the centromeric region of the empty vector and 9.5kb central core (Nott 373 cen8) containing clones before and after swapping. 7

Supplementary data figure 6. First steps in the construction of control strains for the centromere replacement assay. ... 8

Supplementary data figure 7. Timing of mitosis and the cell cycle in S. pombe CBS 2777 strains containing chromosome 2 with a 4.17kb or 9.46kb centromere after swapping. 9

Supplementary data figure 8. Organization of the concatamers of wee1 sequences and CDEII elements established by Minion sequencing. 15

Supplementary data figure 9. CENP-A^{Cnp1} binding to centromere and neo-centromere sequences before and after deletion of the native centromere of chromosome 2 of S. pombe CBS 2777. 28

Supplementary data figure 10. Details of centromere replacement in the laboratory strain of *S. pombe* 29

Supplementary data figure 11. Details of the strains and locations of primers used in centromere replacement in the laboratory strain of *S. pombe* 31

Supplementary data figure 12. Characterization of the laboratory strain centromere replacement. 32

List of Tables

Supplementary data table 1. Sequences used in centromere manipulation and assay illustrated in Figures 2 and 3 of the main text.. 13

Supplementary data table 2. Base sequence composition of sequences assayed and analysed in Figures 2 to 4 of the main text.. 18

Supplementary data table 3. Assaying centromeric activity of centromeric and candidate DNA in the centromere replacement assay 19

Supplementary data table 4. Regression of CENP-A^{Cnp1} binding on AT content across 180 bp tracts for six sequences described in the main text. 33

Supplementary data table 5. Data used in Figure 7 of the main text... 34

Supplementary data table 6. Primers.. 36

Supplementary data, table 7. Genotypes of key strains used in construction or analyses……………………………...37

1 Chromosome engineering of chromosome 2 of *S. pombe* CBS 2777

1.1 Grapical summaries of the engineering of chromosome 2 of *S. pombe* strain CBS 2777 to enable assay of the centromere activity of a candidate sequence

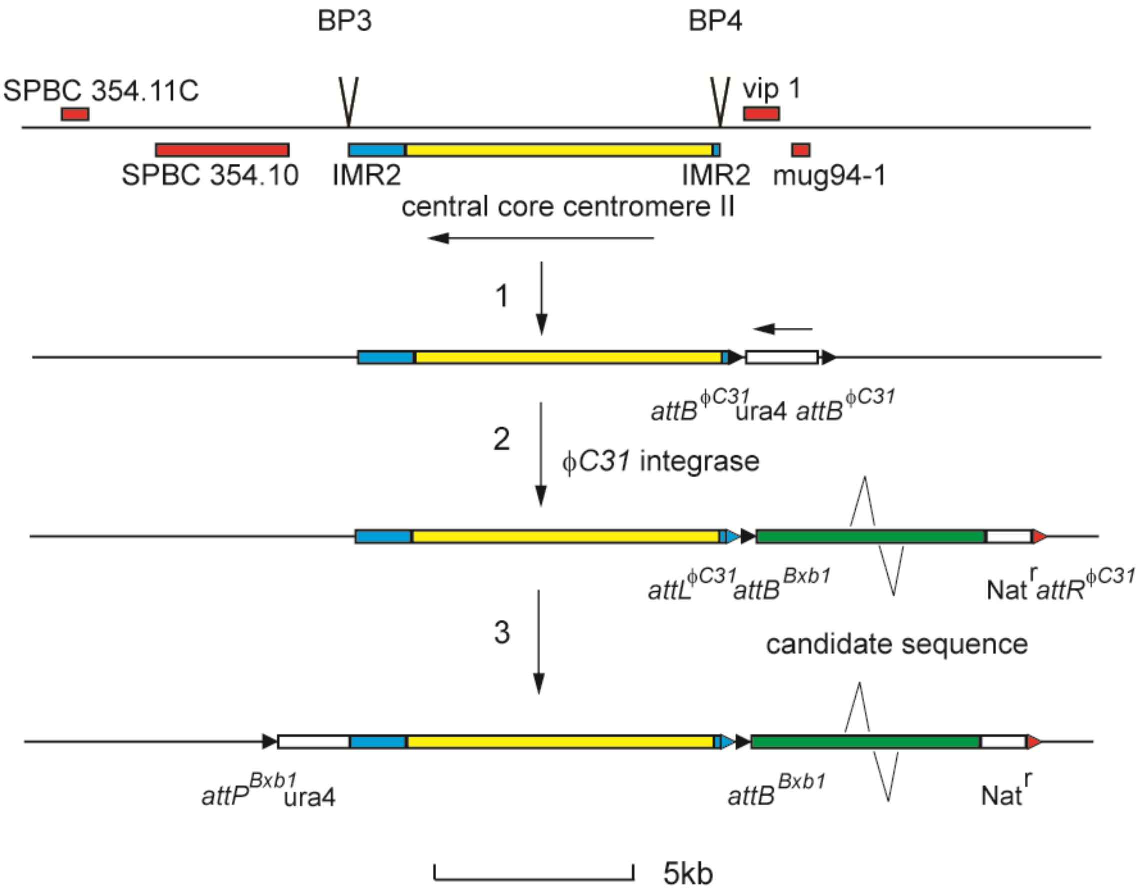

Supplementary data figure 1. Sequence of steps used to engineer a chromosome containing a candidate sequence adjacent to the native centromere

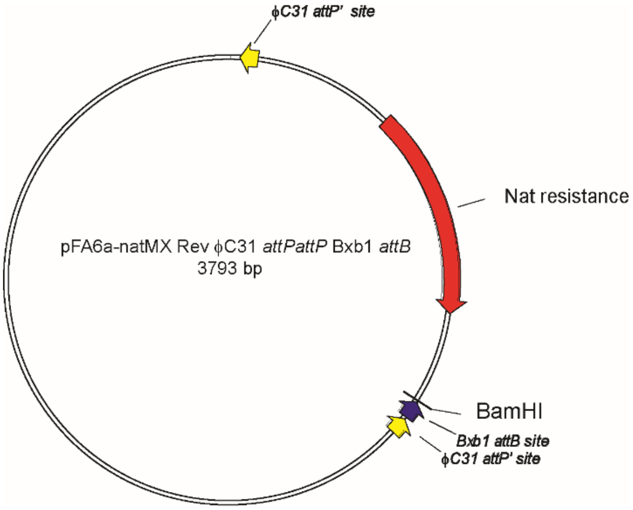

Supplementary data figure 2. pFA6a-natMX6 REV ϕC31 attP attP Bxb1 attB vector used to integrate sequences into chromosomes using site-specific recombination with ϕC31integrase

1.2 Assaying the ability of a candidate sequence to support centromere formation using site-specific recombination.

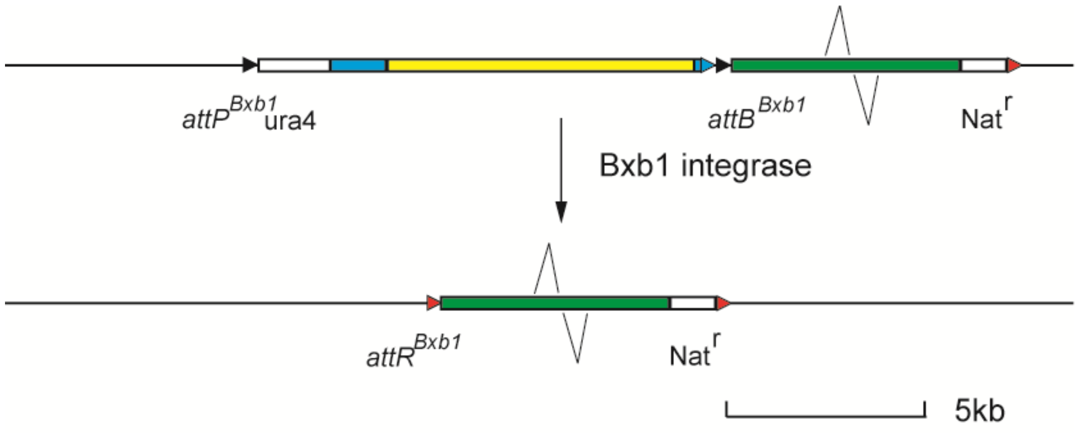

Supplementary data figure 3. Centromere swapping using Bxb1 integrase

As explained in the main paper the assay procedure consisted of the following steps:

1. Transform the engineered strain containing the candidate sequence with either pREP81 or pREP81-Bxb1
2. Plate out onto PMG, supplemented with uracil
3. After 7 days, pick and pool approximately 50 random colonies
4. Count the number of recovered cells per colony
5. Estimate viability by plating on YES agar
6. Measure the proportion of recovered cells that are uracil auxotrophic
7. Measure the proportion of uracil auxotrophic cells that have swapped by PCR, confirm the integrity of the candidate sequence by long-range PCR or blotting. Check the integrity of the chromosome by pulsed-field gel electrophoresis (PFG).

In more detail:

The first step was to delete the native centromere using Bxb1 integrase. This involved transformation either with a Bxb1 integrase expression construct (pREP81-Bxb1) or, as a control, an empty vector (pREP81). The details of the transformation procedure are given below. After transformation, the cells were allowed to recover while selecting for the presence of the expression plasmid and maintaining uracil in the medium to allow cells that have lost the native centromere and flanking ura4 marker to proliferate.

After seven days at 32°C the transformation plates were removed from the incubator. The yield of colonies was variable. Typically between 25 and 300 colonies were recovered in a single transformation but no systematic differences in the numbers of transformed colonies were observed following transformation of cells containing engineered chromosome 2 with an entire central core as a candidate sequence and cells containing engineered chromosome 2 with an empty vector as a candidate sequence. However, the average size of the colonies recovered upon transformation of the cells containing an empty vector were smaller than upon transformation of the cells containing an entire central core sequence (Supplementary data figure 4 and table 3).

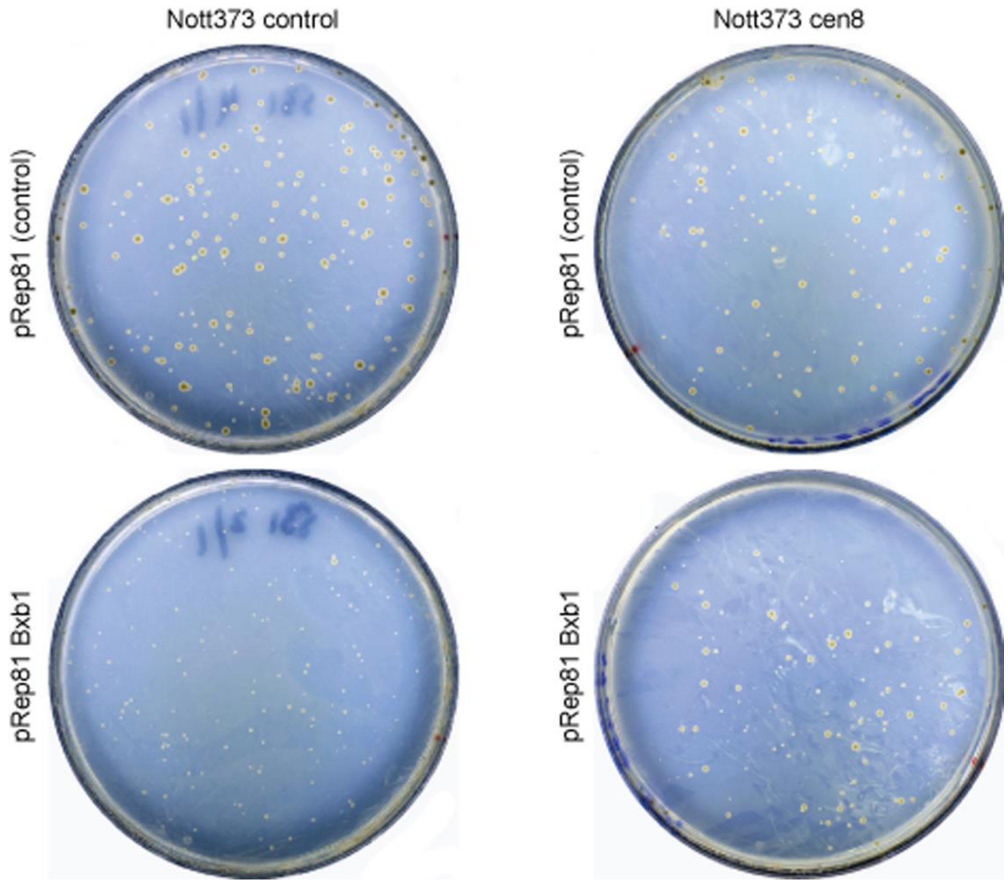

Supplementary data figure 4. Differences in colony size between cells containing an intact central core as a candidate sequence and cells containing an empty vector upon transformation with the pREP81-Bxb1 expression construct. Nott 373 control contained the empty pFA6a-natMX6 REV *attP*$^{\phi C31}$ *attP*$^{\phi C31}$ *attB*^{Bxb1} vector while Nott373 cen8 contained an intact central core as a candidate sequence. Cells of either of these two strains were transformed with either the empty pREP81 expression plasmid or the pREP81-Bxb1 expression vector and recovered on PMG supplemented with uracil. Colonies recovered upon transformation of Nott373 control with the pREP81-Bxb1 expression vector (lower left plate) are on average smaller.

The difference between the sizes of the colonies recovered following transformation of a strain containing the empty pFA6a-natMX6 REV *attP*$^{\phi C31}$ *attP* $^{\phi C31}$ attB^{Bxb1} (Supplementary data figure 4) with the pREP81 and the pREP81-Bxb1 plasmids could be explained by the fact that the integrase was excising the native centromere of chromosome 2 at a rate of less than once per cell cycle and that the cells lacking the centromere were either dying or forming a neo-centromere at a low frequency. In the cells containing an intact central core as a candidate sequence, the loss of the native centromere was compensated by the candidate sequence. In order to test this idea and to assay the functionality of a sequence we picked and pooled approximately 50 colonies from the cells transformed with either the empty pREP81 plasmid or the pREP81-Bxb1 expression plasmid, counted the number of cells in the pool to yield an estimate of the average colony sizes. We then estimated the viability of these cells by plating out a known number (-1000 in duplicate or triplicate) on to YES agar and counting the number of recovered colonies. Of these, a fraction dependent upon the nature of the candidate sequence should have successfully swapped and become ura-. We estimated this fraction by patching onto YES and PMG + Leu agar and recording the proportion that failed to grow in the absence of uracil. Finally, we measured the proportion of these cells that had accurately swapped by PCR (Supplementary data table 3). In at least ten swapped ura- clones we confirmed the integrity of the candidate sequence and the accuracy of the swapping by long-range PCR or restriction enzyme digestion, gel electrophoresis, filter transfer and hybridization (Supplementary data figure 5). Similarly we checked at least ten clones of each swapped type for their chromosomal integrity by pulsed-field gel electrophoresis.

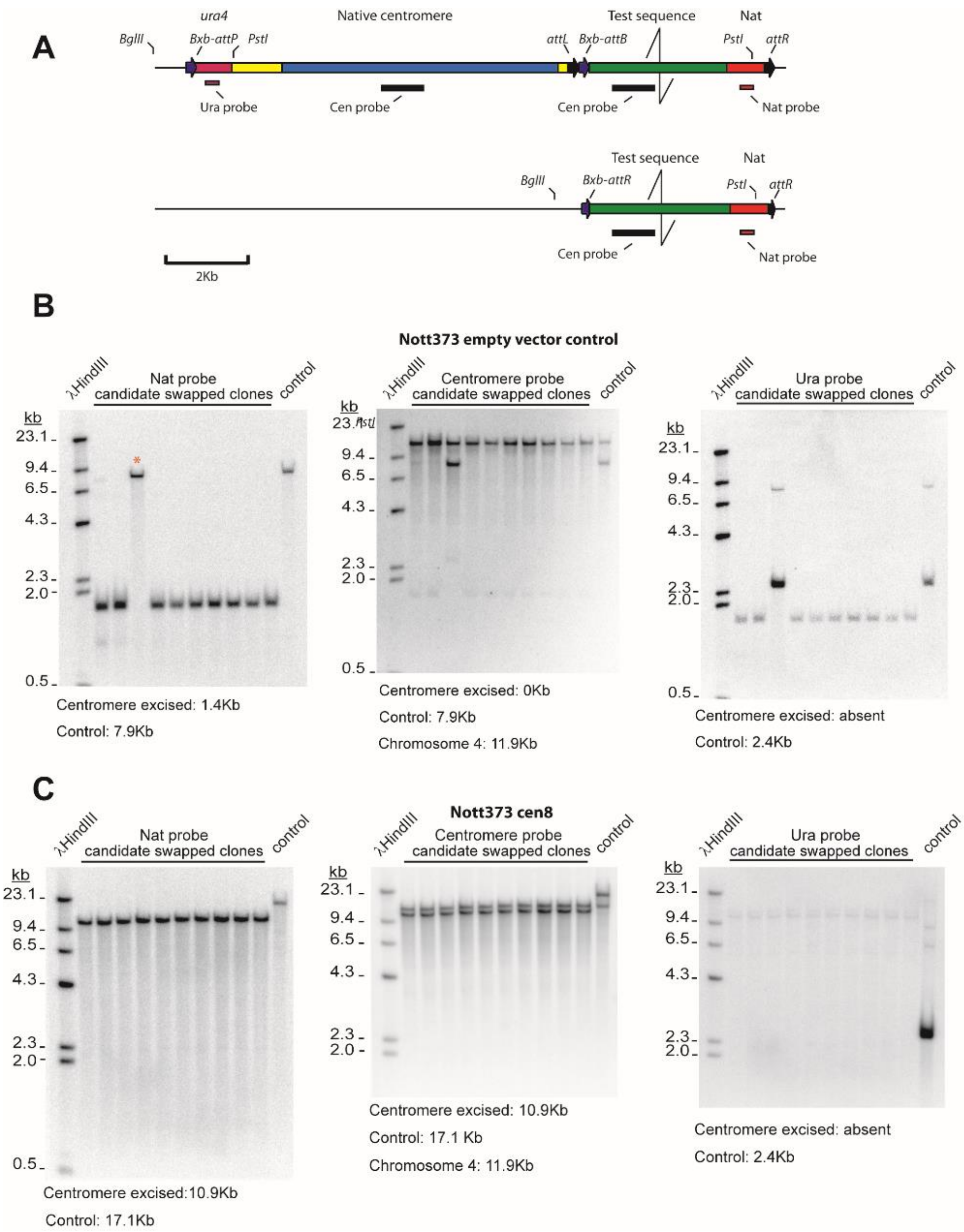

Supplementary data figure 5. Checking the sequence organization of the centromeric region of the empty vector and 9.5kb central core (Nott 373 cen8) containing clones before and after swapping.
A, Sequence organization of the DNA with relevant restriction enzyme sites.
B and C, Filter hybridization analysis of the clones before (control) and after swapping. Sizes of predicted restriction fragments are indicated. The third empty vector candidate swap clone marked with an asterisk is an example of a re-arranged clone where the swap had failed.

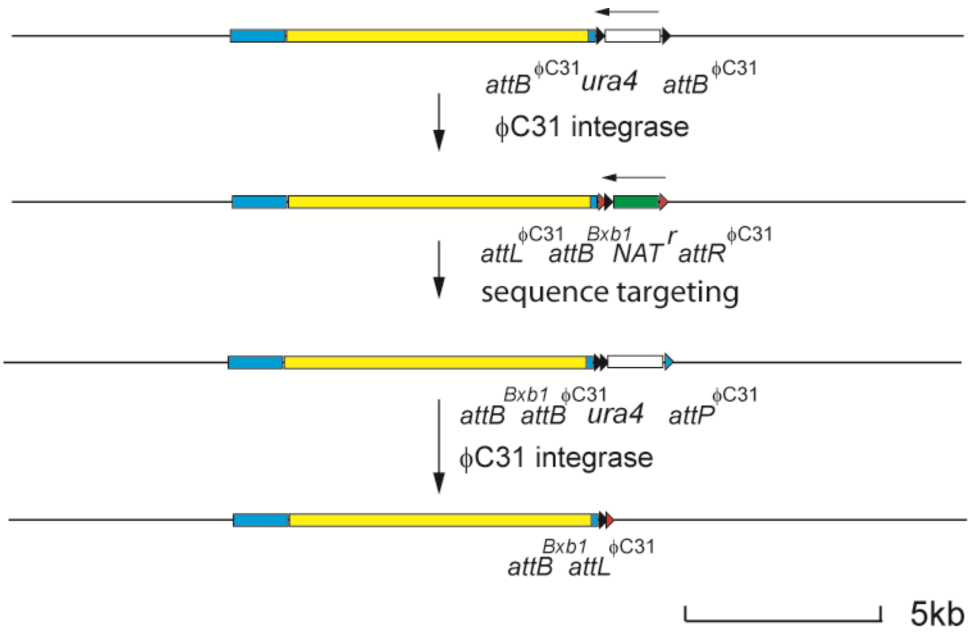

Supplementary data figure 6. First steps in the construction of control strains for the centromere replacement assay.

1.3 Constructing controls for the centromere-replacement assay in which a central core of *S. pombe* centromeric DNA is placed either at the centromere distal telomere or at breakpoint 1 on chromosome 2 of CBS 2777

To construct the control strains the first step was to place an *attB* site for the Bxb1 integrase on the right-hand side of chromosome 2 of CBS 2777 without any accompanying marker. The starting strain for this manipulation (shown in Figure 6) was a strain containing the empty vector pFA6a-natMX6 REV *attP*$^{\phi C31}$ *attP* $^{\phi C31}$ attB^{Bxb1} at breakpoint 4 on the right-hand side of centromere 2. The noursothricin resistance gene and ϕC31integrase recombination sites were removed and the Bxb1 recombinase *attB* site moved by sequence targeting with a ura4 marker gene that was flanked with ϕC31 *attB* and *attP* sites. The ura4 marker was then removed by expressing the ϕC31 integrase to leave a strain that contained a Bxb 1 *attB* site on the right-hand side of the centromere at breakpoint 4. Chromosome 2 in this strain was then targeted with the *attB*$^{\phi C31}$ ura4 *attB*$^{\phi C31}$ either at breakpoint1 or at the neo-centromere locus and this sequence was then used to introduce an intact central core sequence (without a Bxb1 *attB* site) into each respective locus. Subsequent assays were carried out as described above.

1.4 Transformation of cells for assay of centromere activity

The standard transformation procedure was designed to be used for assaying the ability of a candidate sequence to support centromere formation. Cells engineered as above were grown up to an OD of approximately 1 at 600nm in 150 ml of a transformation medium consisting of 5mM potassium hydrogen phthalate, 5mM Na_2HPO_4, 8.5mM glutamic acid, 7.3mM glucose, 2.4mM KH_2PO_4, 1.35mM $MgSO_4$.7H2O, 1.14mM NaCl, 4.5mM $CaCl_2$.2H_2O, 25mM $(NH4)_2SO_4$, 2.44mM CH3CO2K supplemented with 1ml of vitamin stock and 0.1ml of mineral stock (http://www-bcf.usc.edu/-forsburg/media.html). Leucine and uracil were added to 0.25g/l. Cells were washed with sterile distilled water and once with 0.1M CH_3CO_2Li, 10mM Tris HCl, 1mM EDTA, pH 8.0 (TE). The cells were then resuspended in 1ml of 0.1M CH_3CO_2Li, TE, aliquoted into each of four 1.5ml plastic tubes and pelleted in a benchtop centrifuge at 1500g for four minutes. The supernatant was removed. The cell pellet at this stage was typically between 100μl and 150μl in volume. 1.62mg of DNA in 180μl of 0.1M CH3CO_2Li, TE was added to each tube to yield a suspension that was between 280μl and 330μl in volume. The pREP81-Bxb1 integrase expression plasmid was added to two of the tubes and the empty vector to the other two tubes. The tubes were vortexed to resuspend the cells and 2.36 volumes of 40% PEG4000 in 0.1M CH_3CO_2Li, TE was added to each. The resulting suspensions were again mixed by vortexing and then incubated at 32°C for one hour while being slowly inverted using a rotor. 0.39 volumes (with respect to the mixture of cells and DNA) of dimethyl sulphoxide was then added, the mixtures were vortexed and the tubes were placed in a water bath at 40°C for six minutes. The tubes were then filled with YES medium (yeast extract with supplements) (http://www-bcf.usc.edu/-forsburg/media.html) and the cells concentrated by benchtop centrifugation.

The cells were resuspended in YES and the contents of the two tubes containing the cells transformed with one or other of two plasmids were dispersed into 50ml of YES and allowed to recover for two hours at 32°C with shaking. The cells were then pelleted, rinsed in 50ml of distilled water and plated onto three 9cm plates of pombe glutamate medium supplemented with uracil at 0.25g/l. The plates were incubated at 32°C for six or seven days until the colonies became visible and were large enough to be conveniently picked.

It was of interest to determine why cells containing a chromosome with a short centromere were recovered with reduced efficiencies compared to those with a full-length centromere. To address this question we examined the cells with replacement centromeres that were 4.17kb in length. These gave smaller colonies upon streaking than cells with replacement centromeres that were 9.46 kb in length. Measuring the rate of proliferation of one such strain indicated a mean doubling time of 5.5 hours for a clone with a 4.17 kb centromere, compared to 3 hours for a clone with a 9.46 kb centromere. Imaging of live cells with a 4.17kb centromere showed no obvious delay at mitosis compared to those with a 9.46kb centromere (Supplementary data figure 7A) suggesting that the obvious possibility of a delayed metaphase-anaphase transition as an explanation of the extended doubling time could be excluded. Live-cell imaging also did not find an extended cell cycle time (Supplementary data figure 7C). These results led to the conclusion that the small colony size arose either as a result of the accumulation of dead cells or of cells that were unable to divide. To discriminate between these explanations, we streaked the cells containing 4.17kb and full-length centromeres on Phloxin B containing agar plates. Phloxin B selectively stains dead cells but no difference in the extent of red staining of the two colony types was obvious and so we conclude that the reason that cells with small centromeres grow more slowly than cells with large centromeres is that ~40% of their mitoses yield a product that is incapable of further division. Further work will be required to understand why this is so.

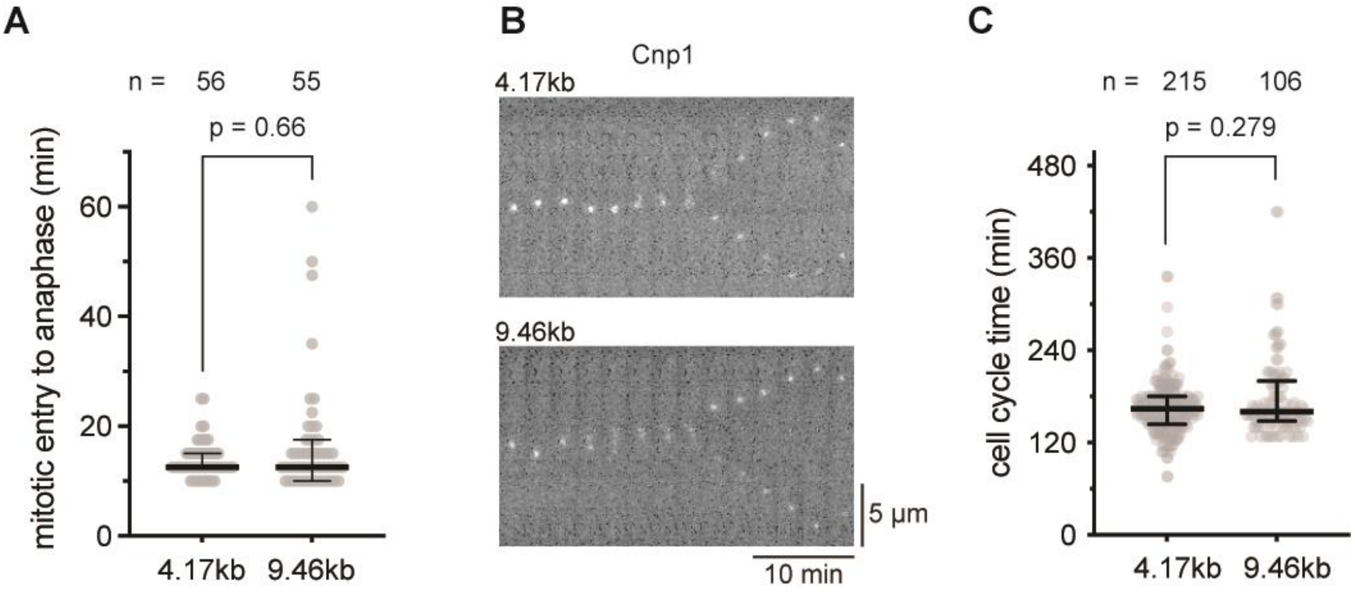

Supplementary data figure 7. Timing of mitosis and the cell cycle in S. pombe CBS 2777 strains containing chromosome 2 with a 4.17kb or 9.46kb centromere after swapping.
A, Time from mitotic entry to anaphase. The declustering of CENP-A^{Cnp1} signals was scored as mitotic entry, the splitting of CENP-A^{Cnp1} signals as anaphase. Single cells (grey) with median and quartiles (black); p value calculated using Mann Whitney test.
B, Representative kymographs of eGFP-Cnp1 as cells undergo division.
C, Cell cycle time was determined by measuring time from one septation event to the next. Single cells (grey) with median and quartiles (black); p value calculated using Mann Whitney test.

1.5 Estimating the stability of the kinetochore on a chromosome with 4.17kb of centromeric DNA.

To test whether the centromeres with the 4.17kb stretch of central core DNA were stable, we used an approach analogous to that used in mutation accumulation experiments (1,2). We cultured 20 sub-clones on solid agar with ten rounds of re-streaking picking a random colony at each round. Picking a random colony at each round of streaking minimises the effect of selection upon the measurement of the rate of appearance of clones containing neo-centromeres by minimizing the effective population size. Given a colony size of 5 x10^6 at each round of streaking, we calculated an upper bound to p the probability, per cell division, of centromere movement from the 4.17kb sequence to the neo-centromere. Specifically, we solve the following expression, $0.99 = 1 - q^n$, where $q = 1 - p$; in order to estimate the upper bound to p with 99% confidence. For each round of the experiment on a single plate an individual cell undergoes 22 divisions and so given 20 plates and 10 rounds of re-streaking we are sampling approximately 4400 divisions or opportunities for movement (that is n = 4400). This estimate indicates that the 4.17kb centromere is stable with a rate of conversion from the centromere to the neo-centromere of less than 0.1% per cell division (with 99% confidence).

2 Sequences assayed

2.1 Central core derived sequences

The sequences assayed in our experiments fell into two classes. One class, of defined lengths, was derived from the central core of chromosome II of the laboratory strain of *S. pombe 972 h⁻*. In order to help discriminate these candidate sequences from their CBS2777 homologues the candidate sequences were mutated by the introduction of 4bp deletions every kilobase in the 9kb sequence to generate a mutant central core sequence;

```
>mutant centromere
caagcttgtaagcataatatgttcagaactctgtcctttctaatggaaaataatacttatttaagcaatt
ttatcagctacgaacgaccactactacgatgtatgcatgtgaataatatttacatttttttattcctatgt
ctactgtttaaactaagtattgtaaatacttataaaattttattatgatataatgagcttgttctttatt
ttgcaaagcaatatggcttgcatataatacataggctacaattcaatgtacattcaagtattgaaaagct
tttcctgtctttccaatttaaaaacactcaacttcggcgacgtgatataagtataggtataataaataaa
gcgtttttnataaccagnttccgcaaanggtaggaaggtttaatcaattcttaaatggttgaaaagtta
taagaaatagtgatccaattaatcatgccatggaataattttataaaccggtaatcgttgcaaagtgct
taccgntttacttttagggcgaaacaataatacaattaggtagtaccagatcgtttatgaaactgctttt
agataggtactttaaaaccacatgaggtttcagtgaacaacgttttgttattttttaagtaatgaactta
aactttctttgtttactggttcttatcttactatcataagtattagtaatgtaattttcggtcaaaaaag
gtgcataaaacgacaaaaatgtgttttaaaatttccatttaatttactcatctcatcaattttgtaaagc
taacgaggtatttatttttttgcttgtttttatttttaactagtttacgttaaaatttcacttatttagtt
acgatgaatacttgggttaatgtaaaaatggaatgattcgaaacaaaattagttttatcacattcctgtt
ttcgtactttgccttttactatataaataaaaaaaaaaaaaaaaaaaaaggaaattgtaacttgaaattt
atgtgattaatttaagcattagcgccttcaataatttatgagtaagatggtaaacaggggtttgattcga
ttataaagggtatggctatcataactttttgttaatccaggcactttttttattgtattctgatttaagagc
tttgatatgatgcatttgaacattttcggtagatttacaaatacaaaacattcttttaattttgatttgg
ttctaattgaccaatatacttcttctcatgaaacattagcgttcatattaaattttatcaaaaatcatca
gttcgaaatcattctacctgtatcacttcaaacaaatgccaaagtttttaacttaacaaaacaaaacgaa
atttttttttgttcttacgtttttcaattactgggggacaaattacgtactaactgttagtttctatg
gtgatttcgttttaattagttactttttttatttattacattcaaaaacacagcttttgctgaacaagact
tgcgtatgaacgcgctaatacaaatataaaaggggttaatttcttcttaattatttacacagaagcgag
cactgtttacatctaacgataatcagaaaattgtgttggcaaaccctagaatgactttaatttttagta
atatgttcttttattgtttagcaaacgtatacttacaaaaggtaatttttactagatttttttaaaataga
aatgatcttttttgcttaaaagtttaatggtaatattgagatggcccatgactataagatgctattaagta
acgtttatctgaaaatatcattatcattgttcgtatcgcaatttgagatttactaaacctttttttaaaa
tgaaaacgcgaactccgataatcattagtacgaaaatcaaaaaaaaaagttgaagggtacgtttaattctt
ggttttaaaattcgacttttagcatcaaagcaaaaacctgaagatttgtatcttattcatttttctttaa
gcggtttagctggttaaagaaaaatgttttccaataatatttattcacatgtactgctttgtgatgcgta
agccgttactcgttaatatgaagaagcattggttagtcctggttgatttctatgtttcctttttggcttc
ggccaattctactttttttaagtattccgtgtgttatctttatggactattattttaaaatctgcatatta
ggtttaagtatgaattagttagggatacgattttctaaatttacatactaataacggaatagatcaaaca
agctccttcaattttttagaaaaaaaaaaaataacgcattaattgtaaatgttttaggaaattattggtcaa
atctatgaatttgctggttaaatagtatatcaggggggttttatgattttggcgaagtgtcttatccttca
tgttaaatgtattaattttcttttttcacaaatatcctgtcgattaaaaaattaaaagtaacacatatgt
agtggaatttatctaaaacctcagaatttttaccgagttgtaaacttgacgaatgacatgaaatggtaaa
agtgagcaagctcataaaataaatccacgcttgtcgcaaaacataaagcaaatttgaaaaaaagaagggt
aaatatattaagaatttatttaaaagcaaatcatttaacttacaattgcctaatgtgataactatatcaa
aaatgcccctagcaaagtaggcaacttggttttattgattaacaaaattatgtataagagattaaattat
cctgtttagaaacttactatttctattacacttgctagatagctaagtattaatttatacaaagccataa
tgttgctaacgaaaccttattattgcacattttgacttgaaaggagtctgtaaaccagctataatttaa
ataagactatagcaaatattttagtttataagaaaaatgaatctattaacaacacccgtcatttatgaaa
attaacaacataaatcaaatatgctatatgttaaaattagtactagcgttaaggtaattcgcataagtga
actaagagtcaaagttattttgaacatagttaataaaacaacgatctaatataaattaattaacgttagc
aaggagttttgttccgttcaggtttactgctaacttcaaatcaagaatcaggccattcattaaaaggagc
aatacagaattctaggaaagttacggtttaccagcatattttgaggtacatgaaattttcatcaaacata
ttgtttatctggataggcactaccactaattttaacatgacaatgcgctctaatccatcaatttatcgat
aaatgattgctttcaccactgatttttttattttatttcttttttttactatgatagtgccgactaggataa
```

acgtgtaaattatatcgatgcatttttagaacaagtattactaaatatgcatatcattcaagtaaaaagt
gtttgaaaattttaattgcgttagcatattctatttgtcctagtaattgcatctattgtactctctcatc
acgtaaaaaattatttgactacagatagattgctgctgagtaacttattcacacgacgtgcatatccatc
cttcgttgaagaatgcaaaaactcaaattagttaaaattttaccgaatgtcccccattaaattgaattt
acttaattcagctttctatttccaacgaaaaaaaatattatttccaaaatccaaattaatttattaaata
acatcaatcaaagacgaaaaaagccttattcttcgagaaacgaaacaataaattacctcataattcagtt
ttctattcctaagaccataattcgaataatctttttattaaaatttttaaatttcttcttagtgatttt
acaacacaaaccgactgttagagtagtaggagaagggattgctgtagaaactgctaggtgtgcaaaattt
aaaagataaacttactttatagactatatgtttataactaaatactgactagactttaagttctaccta
ttactggaaaagttatttcctttaacgtaccttttgacagatcctattactggaaaagttatttccttta
acgtaccttttgacctttaagaaacactaaataaaattgaacgtaaataataagtaacgaattctctgaa
cctttttcgataattcatgtgcttgaataaactaatatgtgcaaaaaatcaataccttctttaactcgac
tttataaaatttcccttaagataataattattagcaatgtttaatttgaaagcaaaaattctggtattat
acttgttaagcgctaactcgtttaagtgaaatacggacaaaaaaaaagtaatgaatgcgcagaaaatatc
aaaatacgggtaaattgatttgtttgttaattgccattctttggcgtattgagataattagcaattgcct
ttttttaatgcgttcatacggatgactttccacgccatgtcatcaattcttttttttttatgtagatacac
ataactcgcatattaactacattagaaattttaaaattaacatatcttgtttgtaatttacaaaccataa
agttttatgataattgttgtgattatcaactttactaatttgactcctaatcagttagaattattttaat
aaatttactaaaacattaaacaaacaacggcacactgtttttttggtcacagcttctaagcatgcaaatga
aattactccaaggaatttgctggcttaataattttatttaataaataaagattatagtagaaaagaatga
aaaagtatttagttgacgatagtatttgttacaaattaaaaactcaaaaccattcttgcagtaagtcaat
cgtgattgacatttctaaaatatcacttactgaacccatgcaagatttagcgcagagggaaacttttata
tctggtagctgagaaacctagtaatgatggtaataaataaatatcaacattcagtttaacatagctataa
acccctaactgtaaccgtagtaaaagctcaaatggctgtatagaagaagttgaaaatcgtttaaagaaac
aatgttgtagtttgaaggatcagttggtaaaacaaatctattatagaaattatattaatttcagagagcc
tcatattttacgcaaattatgaaggtaaatattgcaatcagaccatttgcgaaacagtaaatttcttttt
tctgtttggttttgcttgtgatttggttcatggtttttattaatattttcaagatagtttctaaagatca
tccagaacaatttctattccttcattatttcgttgaaatgaccagatcaaaataaaattctcgaaataat
atttttgcaatttttttatgttttattttatatgcaaataaaatgtttacatggaaatcccatatatttaa
aactgactaaagcgtatttaagacgtaatctaggacccctacgtttttttgtttcttgcagttcgaaaaat
cgtgcacatttgtgaaaaggtttagctctacctatcgcaaataaatgctgcatcaaacgcaattgctgct
atgtgggtctgtattgcctctcccttgccagtaatgtgtatttcatcttgtttatttgttttcttatcga
aattaacttaagctttgtccaaatgctaattattaaccactttgggtgtatacgaaacacatattttcaa
aacaccgcaacgaaaaacaaaaaattgtcccttaatatttgttgattaaagtatcgtgaaaggctttttc
ggaaataatctaaaagaatctttaacacctgttaatacttttacaaactaatcgcgagaaaatcattaag
agtttagaataaagtttcctagcaatattttgctatataaatgattttctggttaatggttatagcagaa
aacacagacattttttgtacgtatgtacattaactatgtgaataaacatcttagtacgggttcatcagttc
aataaatattcaactgtattaacaacatgtgcgtccgtgaacatataatgaaagtcaataattatcatga
agctatattttcagttaaaagtttacccagtagatagctctgaagaaatttatttgatgaacagtctaaa
attcaatgcgaaatccttttaattaatagtgactaatttagtccaagctttcgcgtgtttgcttactttt
attttccctactaaaataaaatctaaaaatttttattcctggaaatcagtaaaactcatgatttcaaaga
aaacagccaaaggtctcagctgctgctagtttgttcgtacgaaaaactgttttcaatattttgactataa
ctagaccactcagattgaatttttttgaaaattttgtacnttcaaattgcgtcaactgctttaattgtctg
aaatacttgatttgctttcttgagtattcatctagttttatcattaaagctaggtatttccaagggctcc
taaaactaatgattagcgatgtaatatcttaaatataccatttaagttgatgaatgtatagtatgtttta
ctaggatacacggtagaacacatatacttggatgttatgcatacacatcgtagtaattggtccattgttt
ggattgaatgtttagtatgagaactgtatggtggtataccaaaaaatttttacgtttttgaaagtaaaga
tatgattaatatctgcaatgttttcttttagattatggaatctgttacaacagcttaaatggtagtttaa
ttaacaaggtatttactttttattgtttttttgcagctcctccaaaaaattaactagcctagatcacaat
tatcagtcaatctgaattaatagacgtaagaataacttcttaaaaatacagaaaacaaaaaaatgacttc
ataacgtttgtataaaccagatttaattaacggccaataagtttagttacctgttagcaaaaaataattt
taatactgccactcgtcacctttagtaaccctgtaaccgtaattcagcctgtccatcgcaaaaggtaacc
cccatcagtctgtaaatagtaatcttagtagtgcttcaaatattaaaaacggattcgcattctctcaaga
tgtcgaatatgcagaaaaaactttcattattaattattaaacgtaaatctatttacaaatctcaacattt
aaaagttttactaaatataatttaaccataaaataaggttgatgttgaattattctcatgtacaacccaa
aaaaaaggcaatcctacatcattggaatttttaaataaattctttggattcgaaggtctttactcgttt
ttaaaaaaattgatctgaattggcttattagatgacaatatcaaatatgtaccgactcagttgacgttac
ctttatcaaatttatcgcactttaaaacttactaaagcagaagaaaatatgctgaactatttgtggtgga
cattgataatgtaaattacacacttaacgaaattatctagttacaaatatataagctactagtcaataaa

```
aagctgaaggctcaaatcattacgtaaaatattttgatcgggaatacaaatactaacttcttgagaaag
ctgcataaaaactagtgtcaatataaaaagcactagtttttcctaatattgactgttgtttacaaatttg
tttactgagcattcagcaaattgcaaaagcctggttaaaaaggaagacgactattctaaatggtatttgt
aaaatgcttgctttacttaattaactaataacatagaaaaaactacttttaattttgatcatataactac
agatagaaaaggcagtcaggcaaccagttaacaaccttttagaagtaatgcatacttaagttatgaaaaa
aaaaaattcaagtccacttccaatcccataaaaatgaaataaagcaaacagcagtaaccttgtaaagcac
taaactcattactagaaacagtgaggtgcagggaataaatgtacataatacaaattaagccgcatataag
gacgcagcgagcaaaatagccttcctaatcaaaataactaaaaataaagataaaagaagaaaattcgaat
agtgtaaaattacacacagaaatacttttatgcgagactttcttaggatatgaattatattaactaaata
gcaactgactaatatgtattttgttttgttaagttaaaaactttggcatttgtttgaagtgatacaagta
gaatgatttcgaactgatgatttttgataaaatttaatatgaacgctaatgtttccatgagaagaagtat
attggtcaattagaaccaaatcaaaattaaaagaatgttttgtatttgtaaatctaccgaaaatgttcaa
atgcatcatatcaaagctcttaaatcagaatacaataaaaaagtgcctggattaacaaaagttatgatag
ccgtttatacccttttataatcaaatcaaacccctgtttaccatcttactcataaattattgaaggcgcta
atgcttaaattaatcacataaatttcaagttacaatttccttttttttttttttttttttatttatata
gtaaaaggcaaagtacgaaaacaggaatgtgataaaactaattttgtttcgaatcattccatttttacat
taacccaagtattcatcgtaactaaataagtgaaattttaacgtaaactagttaaaaataaaaacaagca
aaaaataaatacctcgttagctttacaaaattgatgagatgagtaaattaaatggaaattttaaaacaca
tttttgtcgttttatgcacctttttttgaccgaaaattacattactaatacttatgatagtaagataagaa
ccagtaaacaaagaaagtttaagttcattacttaaaaaataacaaaacgttgttcactgaaacctcatgt
ggttttaaagtacctatctaaaagcagtttcataaacgatctggtactacctaattgtattattgtttcg
ccctaaaagtaaacggtaagcactttgcaacgattaccggtttataaaaattattccatggcatgattaa
ttggatcactatttcttataacttttcaacatttaaaattgattaaacttcctacatttgcggaactggt
tataaaaaacgctttatttattatacctatacttatatcacgtcgccgaagttgagtgtttttaaattgg
aaagacaggaaaagcttttcaatacttgaatgtacattgaattgtagcctatgtattatatgcaagccat
attgctttgcaaaataaagaacaagctcattatatcataataaaattttataagtatttacaatacttag
tttaaacagtagacataggaataaaaaatgtaaatattattcacatgcatacatcgtagtagtggtcgtt
cgtagctgataaaattgcttaaataagtattattttccattagaaaggacagagttctgaacatattatg
cttacaagcttg
```

Thus it was possible to discriminate between ChIP-seq reads mapping to the native centromeres of chromosomes 2 and 4 of the CBS2777 derived strain and those mapping to the candidate sequences.

Subsections of the mutant centromere were amplified with the primers indicated in table 1 below and sub-cloned into the pFA6a-natMX6 REV *attP*$^{\phi C31}$ *attP* $^{\phi C31}$ *attB*^{Bxb1} vector (Supplementary data figure 2). The 0.89kb sequence that was assayed as concatamer was amplified with primers (1240 and 1241) that included BamHI and BglII sites and was concatamerized by sequential BamHI and BglII digestion and ligation. The results of assaying the activities of these sequences are given in Figure 2B of the main text.

Sequence; numbering with respect to lab strain chromosome II	Working name	Defined by primers
9.46kb: intact central core	Cen 8	247
6.8kb: 1,620,855-1,627,609	Cen 5	784/785
4.17 kb (left): 1,620,855-1,625,025	Cen 1	784/787
3.59 kb (right): 1,624,025-1,627,609	Cen 2	785/786
2.0 kb: 1,624,025-1,626,025	Cen 3	786/789
1.0 kb: 1,624,025-1,625,025	Cen 4	786/787
5.5 kb: 1,622,123-1,627,609	Cen 11	785/1412
4.4 kb: 1,623,232-1,627,609	Cen 12	785/1413
4.19 kb (right): 1,624,025-1,628,216	Cen 22	786/1489
3.56 kb (left): 1,620,855-1,624,410	Cen 23	784/1490
0.89 kb: 1,624,556-1,625,443	concatamerized	1240/1241

Supplementary data table 1. Sequences used in centromere manipulation and assay illustrated in Figures 2 and 3 of the main text.

2.2 Candidate sequences

We assayed five different candidate sequences, two of which were derivatives of the 0.89kb sequence that was concatamerized as described above. The first of these sequences is the GC rich sequence which was a concatamer of the following sequence synthesized as a gBlock by IDT. The original sequence from IDT included primer binding sites and was amplified and concatamerized by sequential ligation and BamHI, BglII digestion. This sequence preserves the integrity of A or T tracts greater than three residues in length.

```
> GC Rich
GATCCTTTCGGCGCGCCGCGTATTTTACGCAAATTATGAAGGCAAATATCGTAATCGGCCCATTTGCGAA
ACGGCAAATTTCTTTTTTCCGTTTGGTTTTGCCGGTGATTTGGTTCGTGGTTTTTATTAATATTTTCAAG
GCGGTTTCTAAAGGTCACCCAGAACAATTTCTATTCCTCCATTATTTCGTTGAAATGGCCGGCTCAAAAT
AAAATTCTCGAAATAATATTTTTGCAATTTTTTATGTTTTATTTTATACGCAAATAAAATGTTTACGCGG
AAATCCCATATATTTAAAACGGGCTAAAGCGTATTTAAGCCGTAATCTAGGGCCCCTACGTTTTTTGTTT
CTTGCAGAAAGTCCGAAAAATCGTGCGCATTTGTGAAAAGGTTTGGCGCGGCCTATCGCAAATAAATGCC
GCGTCAAACGCAATTGCTGCTATGTGGGTCTGTATTGCCGCTCCCTTGCCGGTAATGCGTATTTCGTCTT
GTTTATTTGTTTTCTTGTCGAAATTAACTTAAGCTTTGCCCAAATGCTAATTATTAACCGCTTTGGGCGT
ATACGAAACGCATATTTTCAAAACCCCGCAACGAAAAACAAAAAATTGCCCCTTAATATTTGTTGATTAA
AGTATCGTGAAAGGCTTTTTCGGAAACAATCTAAAAGAATCTTTAACGCCGGTTAATGCTTTGCAAACTA
ATCGCGGGAAAATCATTAAGAGTTTAGAATAAAGTTTCCCGGCAATATTTTGCTATATAAACGATTTTCT
GGTTAATGGTTATGGCAGAAAACACAGACATTTTTGGCCGTGTGCGCATTAACTATGCGAATAAACATCT
TAGTACGGGTTCGCCGGTTCAATAAATATTCGGCTGTATTAACAACATGCGCGA
```

The second sequence that we assayed was a concatamer of the following

```
>Long tracts of A or T mutated by substituting alternate As with C and
alternate Ts with G

GATCCTGTCAGAGAGCCTCATATGTTACGCACATTATGAAGGTACATATTGTAATCAGACCATGTGCGACACAGTACATGTCT
GTTGTCTGTGTGGTGTTGCTTGTGATGTGGTTCATGGTGTGTATTAATATGTTCAAGATAGTGTCTACAGATCATCCAGAACA
ATGTCTATTCCTTCATTATGTCGTTGACATGACCAGATCACAATACAATTCTCGACATAATATGTGTGCAATGTTGTATGTGT
TATGTTATATGCACATACAATGTGTACATGGACATCACATATATGTACAACTGACTACAGCGTATGTAAGACGTAATCTAGGA
CACCTACGTGTTGTGTGTCTTGCAGACAGTTCGACACATCGTGCACATGTGTGACAAGGTGTAGCTCTACCTATCGCACATAC
ATGCTGCATCACACGCAATTGCTGCTATGTGTGTCTGTATTGCCTCTCACTTGCCAGTAATGTGTATGTCATCTTGTGTATGT
GTGTTCTTATCGACATTAACTTAAGCTGTGTCCACATGCTAATTATTAACCACTGTGTGTGTATACGACAGATGTATGTTCAC
AGCACCGCAACGACAGATACAGCATTGTCACTTAATATGTGTTGATTACAGTATCGTGACAGGCTGTGTCAGACATAATCTAC
AAGAATCTGTAACACCTGTTAATACTGTACACACTAATCGCGAGACAATCATTAAGAGTGTAGAATACAGTGTCCTAGCAATA
TGTTGCTATATACATGATGTTCTGATTAATAGTTATAGCAGACAACACAGACATGTGTGTACGTATGTACATTAACTATGTGA
ATACACATCTTAGTACGTGTTCATCAGTTCAATACATATTCAACTGTATTAACAACATGTGCGA
```

In addition, we assayed a concatamer of two sequences in which the CDEII sequences of various *Saccharomyces cerevisiae* centromeres were embedded in segments of the *wee1* gene. The final 5.9kb concatamer is termed “artificial centromere” below and in the main text. The artificial centromere was sequenced by Minion in order to generate the sequences illustrated in figure 8 below. The sequences of the units used to generate the concatamer are shown below. The regions of CDEII have been underlined and separated from the *wee1* sequences by forward slashes.

```
>wee1-CDEII_1

GATCCCGTTCTCTAGAGA/TAAATAATTTTAAAAATA/CACGGTTTAGAAATGTTACT/TATTTTTAATAGTTTTTAAA
TATTTTA/GGAAGTGGAGAGTTTAGT/GAATATTATTAAAAAGTTTATTA/GCTTACGGCGGTCCCAACGC/AATAATA
ATTTAAATTACTATTTTT/CCATGAATCTAAATCGTGCT/TTTTAATAACACTATTGTATTTG/GCTCCTCCAACTCCA
TC/AAAAATTTAAAATACTTTTTTATT/GAGCCTGTATGATGCTAACAA/ATTTAATTATTATTAAGTAAAAAA/TTCT
ACTTCATCTACCTCTTC/TATTAATTAATTTTTTTTCTTAAA/GCAAAAGCCAAATACCTCTT/TTTGGCAGATTA

>wee1-CDEII_2

GATCCCGTTGGCTCTAG/TTTATTTTTAATTTTTTTTAA/TCACTAGCTTATTCGGTCCTCG/TTTTTTCTTTAAATTA
AACAAAAA/CAAACAGACCACTTCCTCCC/TATATTTTTAAAAAAAGTAAAAAA/TTCCTTCTCTCATGCCGCCCT/TT
TAAAAAATAAAATTTAAAATAT/CTTCACCCTCTTTCTCCTCC/AAAATTTTAAAAAAATTAATTTTCTC/TTTTACAC
ATTCACAACCAGA/ATTTATTATATTTTTTTAATTAC/ATACAGGCACAACCTGTACCT/TTTATATTTTTAATTAAAT
```

TTTTA/TCATCATCTACTTCACATTTG/TATAAATTATTATAATATTGATATTT/TTTTTGATAGACCCAATCTG/AAT
TAAAAACAAATTATTAATGGTT/GTATCACGCTCTTCCTCTC/GGATTGGCGA

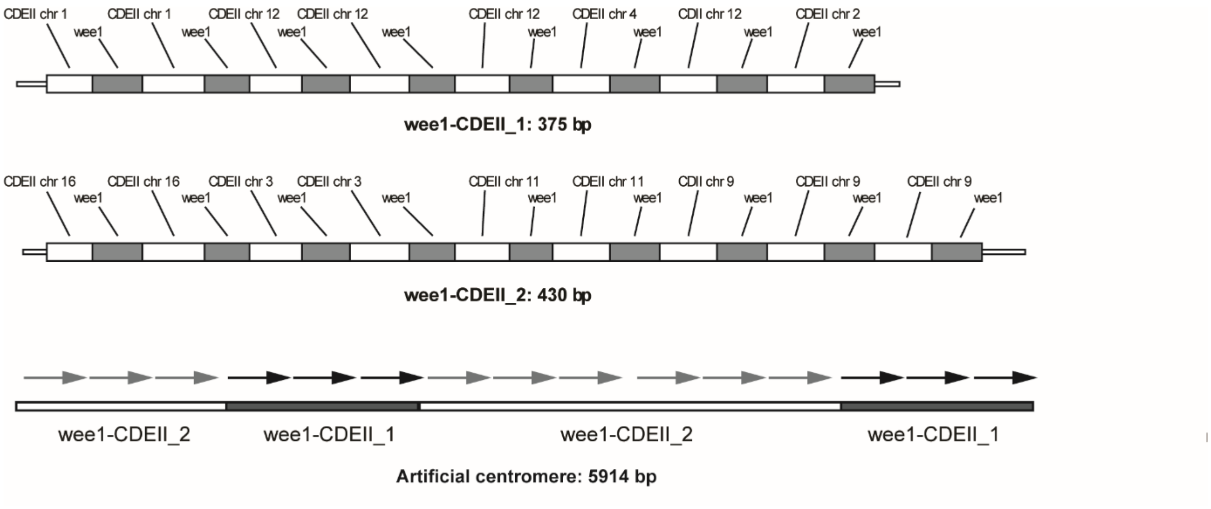

Supplementary data figure 8. Organization of the concatamers of wee1 sequences and CDEII elements established by Minion sequencing.

In addition, we assayed the functionality of the human Y alphoid sequence (GenBank: AF522078.1) and 5.1kb of *Clostridium acetobutylicum* strain LJ4 chromosome (GenBank residues: CP030018.1) residues 3687849-3692959 and 9.0kb of neo-centromeric DNA from CBS2777 chromosome 2 from residues 3862676 to 3871659

>neo-centromere sequence assayed in experiments illustrated in figure 5 of the main text

ctgacgacggactttgtgaaggaatcgaatcaaaagatggtttaatcattggtgtacaatggcatcctgaagcaatcat
cgataaacaaccgcatagcttaaaattatttcaatattttataaaccgctcaaagtggcatatgaaacaatccgatata
ttttctaatgttctaccccacgagtcaagttttaccgaaattcaataatctctgttccaattgctccctaagtacttg
cttgctctttttttttgctgaagtgaattttcggattgttgagaatttcagcgttgtcattatgttgtatttaattcaa
atggaaagtcctcataattttgcttttatttatttattgcatagtactttctaagtttatatttaattgtgtttatg
aaatagagatttcgtcacactatctatatcagcgaaggattgcaaccgaagagattttagtgtaaaaatttcgtaacc
accatggcaattctaatctatttcgttaatcaaattatccaatttattgccaaggttggattatggacatgaaaaaact
gtaactcctcaatatcaacactgacaagatactttgtattttaaactatatatttatttatagggtgcttaataaaat
taccgagaaaatggttattcaagaagcgtgcatgtaatttgtaaagaaaataaattgtggacagaattttaaattaaat
aagcgataagccatcttagacttctcataatcaggcatcttaaatgtatttgggaagcaaaaacttaaattcataattt
tctaaatacattgatctatttaacttaaggagtgaataatgatgatacgtataactcaaagtgccagtttcctttgcaa
tgtcattactgtcaaaggaaaaaatgagcaaactttgcttaagtcataaagtatatataatgcttaaaattatctggcc
attcaaattttgacttgttctcctttgaagatagatttatgtcgataatctttgatacatacttgaatatctgatcatg
gacataaacagatggcaacgaaagagctcttaattatttttttatttttgggagaatagaatatatttgctacggtta
tccaattggttcatatattggtataaaactcaacatatttaaagtgctataaagataaaaaaattgtatatagcgagca
atatataaatttcatacgatctaaaatcacgttttaatttctaataccaaaaaaacgataacctttctatatcctaaag
ttaatagtcaatgttttagcaaatagatgttcaattttatatacttgtgaaaatggaaaatcaaatttttttttattta
agtaagaagtgcttataatgtctttgatatcttatcacgatcgaagcttgttttgcatatattcagtccgatttataaa
aaaaattttagttagtagtgcgaattgaagttcttttaataagaactctgctacaaacacacttcagagaagtttgcat
gattcttactatgaccaatctcacacttcagtattttaaagaaacataatccggcggcatcttcttaccaaatatattt
taaatctgttaatcttggaactaatatgtagctaatttggggtaaatatagctattatatgataacttgcaataaatta
tgaacaaaaggaaattgatattttcattaatgtagaaaaaataattttcaaaatgattttaatgtctccaataaaaact
cgtttaactatataattttctaaatgttgacgatatattcatttttaaaaatataacatgtaaccaacagttaatttat
acattctaactgcaaaagctataatattcaacctatcaagtttaaacactaaaaaatctaagatatcttcgaaacataa
atataaagctagaagttaatcacaattatcactccattcttgttgaacaaaataggcaaatgaataaggagaactgtcg
taaccatcatcacgaacccagtattgtgtaacgtttgaagaagtatataaagtgtcatttgccatattgttcaaagcct

ttagcacgatactttgaaggaaattgaggaagacaacatcaattgtttctggcgaggaggtagcattaaacccagcttg
ggcttgaagaatgttaggagtagcatacgaaggaacagaagtatgtactgtatcatctccatcttcaccaaaatcatca
cttgtaacataaccgggattaagggcagccaaattttcatttatattgcgtaaattggatcccccgtttaaggtcgagt
ataacttcttagcgtatttataaggaacatctgccaaatacatatagtaattagtaagaaaagggtttaattggaacat
ttcgtcgggtccaaattttccttgatacatggaacctcgtataccacctccattgctaatgataattcggggcacagtt
gaacggtttcgattggtaattatttctggcaaaacacgatcagtcataagcttaaaaatactgtcatcagattgaggag
agacttcagtcatataatagttttaggaacgcatccaaagacatatgagaggttaagatcgtcgcgatactgtttaat
gatttagataattcaatgccttctggagtatcgaaggaatcttctgattgttgagtatggaaacggaagttctggcga
ttgaaatcaatgtaacggcgcgtgtaatacagaggtgtatttggaatagggagattgggatacgattggcgagtttcat
tggtgacgggacgaccaacatattgacgggttgcattggatgctgctaggccatcaattgaaagccagcctacagtttc
acaatatctaccaccctcaagagatacagaagactcgtcgtaaacagcaaagtcacgaatatgagagtgaccgccaaag
atttgaatgggagtatcaggatgcacttttctaattgaggcatgtagtgacttccattcgtcccaatcgcgaaccggaa
tatgtccaagtaataagaaaaggtcaacatctgttctatttatttgttgttgataccatctggagttgacggctgtctc
aacaggtgtaacaacagtgttgttggcgttaccggtgaaatcgtagaggaagcctacagcaagaactcgaacgccatgt
ttcgtagtaaagtaggcagattcggctgcaaattgctccaactcatttgatgagttgaagatttgaacgttagaagcga
gataagttccattccagtgaggtacaaaatattcgtgggtattattagaaacggatgcttggtacaattcgtgattacc
aatcgtaagaatatcatagggtaggtacgtaaaaatgttgtttgtatatattccttctggatcagaagcatcagaaagt
ccgtttccgtcatgtaaatcaccggtatcaactaataataaatctacgtctttaaaatcagctaattccttcatccgca
aaacaaaagacttaaattcgccaaagtcggctttataacgagaatctctaaggtgaccaccaagccaaccgtgagtgtc
cgtagtatgaatgaaattcatttgaccccattctaaaggtttaatgatttggttccaatcatacattgtagttaaatcg
gtagacgagtaagcaattacagaaccaatacaactgaacaatagcacaagggttgaccaaaaatgtatcgaggctgtct
tcatgttgatggagtcgggataaagagcttaacaatctatttcgctgtttaccgagggtaagattctttaatttataca
aaaaaaagatcggcatttcgaaattcaatgttttcaaatttccctcagtcaattcattaacatcgtacaacctttgaaa
agtgtcagtgtcaaaaaaaggtttacaaaactttgtcggagatttcagtcccatttgctaatgacctaatgtcaaaat
ttagtaatgaattgaagttgcgaagcaactttgagtttgctgcaaatgtatcttcttacattatattaatttaaacaat
caaaattcataataattatagacagtgttatttcttcgttcccttactcaaacatttatttaagtaaaaaaactcttta
gcaatgggaatgactcggagttcgatgggaaaaccgtaacatcagtctcaaaatactgttaatggaacatttgtgtttt
gcattatgtcatttacgtccttcggtatataataacgctatgttcggttgacaaaaagttgtcctaaaagtttatttt
aagacatgcactttctgcttacgattaaaaaaaaaaaaaaacattgtcttgctaagctataattgaatatttgtatg
agctaagaattattgttagagctgtgagaaccagttctagtacttcaatgagcttctactatgattgttgtccatatta
aacattgtatcggattcaatctctccgggaaacaccaagccgagcggaccatgaatgagagcatacagactttcgattc
tgttcattacgaaaaatattaggtagtacatagccgacaaatcacttaccggtaggatatgccaatatcgacaagcgta
tgggatggtgcaaaaacggtaatatttcaaagttggcttcaataatagtcgacaaaagctctaagtttaaaagacgaag
tttaataacatttgtgatggtttgcctttttacatcaagagaaatttcattatgcatataaaacaaaagcatatttga
attaatcaaaataaccaattattgcttagaaccaaagaaaccccgatttacgcaattaacttaaataaaattattttgc
aaggaatattcgactttcgaatggaagtattttaacttcgacgaacattgaaatgacattgcaagcatttccgttcaaa
ttgttcaatattttataaaagtcagaagttaagattagaactaatatattgactactttttttgactacttcaactta
ccggagctagactactccgacattgcaaaacgtgagattggtttatcaaatatttaccactttaatatcagttattttc
aaaatatctttactttatttgcaattgctaaaagctagttttcgaaatgaaattcatcattggaagttagagcttaat
atctagtccgtcactgagttcttcatgaagtctgtcgtacattttgagagataaagaatatgaaatttgtcatttatgt
tgcttgtttgcccaaattagcattttattgacggtaaaggataatgacataataaaaaagagggatgggatagaagcgg
aagtagtattgctcggcatattaatatgattttcaagagttaagacggtttatggtatataacttcagcgagctaagc
acattaaattcaccaggaattaagcagaactacaagaaggaattctttaagaaattttggataaggtgacttttaccaa
tatattttctgaaatcacatattcaaatttgtctaaaaaaagaataacagcaaaaaaagcatacagtgtactgtccacc
aatattactaaagaaagtatgagcagaaacaatcatgaattgatccaacatcgtctagacattgcgtaaaagcaataaa
atataaaattgaacttaaagaacctttcatattgacaataaagtatcattaatgtagacggaaaagatacaatactaa
aactttttgaaagggttcaaaaaaattgagtaaatacttctatttaaccattaacccattcaattcgattttaagctgg
agtcacagataaaataagtaagtgattacatattctgtttgaacacactcatgtcgcatgttctgtaaaaaaggttgaa
atttaaaatgtagtattttgagtgatcattggacattaatgctaacttactctttaacttaattcctaagagggagtat
actttttctttaagatatccgtaaaatatagctttctagttctctattttctcgacattattaaggttcacattcttaa
aagttacaaacatctttaaaattgtaaaactttttaagatactatagagttcattaactttattaaataagtatatag
taaataaccgaactctgaaaactgcagctttcgttttccggaaaatagcaacaaaagaatttgattacaaaaatgagaa
ttcataaaaaaaacaaatgaagctatgaagggtgaataaattctacagccttgtcgcaatgacatgttggtctactcta
tctacgatcgtactcgtagacacagtagtaatatttcaaacaagtaaaaattaaacgatcagcaacttaccaacatgta
cctttcatttttgactgtgtttaatagtgtatttttacattactattaagaatcgagtatatatcgatgagttcaaaat

tctgtaatatcaactcaaaagacttggaccaaagatagcaacaaaattacaaataaaccaacgctattaacaagtctcc
aaattccagaataagctctgtttatatccggctggaaaaatctccttaactgcaaatttttcctttgtgcaaataaaat
cacagaaaattaaaaaaattagtaacgattattagacctttctttttcacgagggtaatgctgataataaagttcagt
tattaagagatcagtttcctttgaataatcctatctttcttttagtgaacaacaaactaagatattttctgatatattt
taataataatgaattcagactatatacataaatgggcaatgaatccgaatagagatggttaaatgcaaaacaatgatga
aagcgagaacagaaagacatattttccataaaatcaataacatactttgtaatttttgttagctttaagattgattgtt
gtctctaattgaaagggtttccccgttgcgagtaggtagagtatgaaaggcaatctcatctttaaatggttcaatcttt
aagtccaaagtcaatattactactttatagagagcccaatggctgaaatacaaaagaccggttatagttgcacccaagg
ctgctataatcattcccaaggctgcacccaggactgctatcatcataccggtctcccgtaaagcaagcaccacaaactc
tgcagagactattttgaattattagcaaaaaaaaggcatcactgtaaaggaaacatacaaattaaactaacaaacaata
aaacaaacaaacaaatgtaaatagcaataggcccccaaatttcactgtaaaaccgattttcatttataaaactttcatt
tatccaaattaaaagaaaatctttgacttcaaagttcttgtctattgcgaaaataattaaaatacaccaaataataagc
aagggccaacgaagtttactgcgggtagaaatcctaccatcaggataacaactatcatgaaatttagaaatttcagtaa
aaaggttatttaaaaagacctttattttatctgtcttaatgtttataatatcttcttgaggcttgatggtgttggtaaa
gagattttgtttgtttaccttttcaggcttacacaattcaagtccccgctccaggtcaacattacgagtatctcttacc
ataaataactcattgtaaccaggaggttcaacttgtttttttgggctttctggatttgacattggaataaaaccacagc
actgcaatttgacagctttagcaccactagtaatgtttgacaatataaaattaaacagtgtggtttatggaagataata
agaaaatataataaaatattttaagccataaacgaaagaattaaaaaaagatcaaaattggtgaatgttttacgtatcc
ttaaatcagataccaaactgcgtagctttctttattataccgaaaacattcacactgtctacaaaaattactaagaata
ctataggctgcgatgagttttcttagtgatttttgtttttttttaaggtttacacattgaatgaaaaaaaaaaaaaacag
tatatgtagcatcaataggacttaagcatttcgacagatttaatatttattacgaaaatgttcatcgaaagtagtagaa
gatcttccaaatatcttcagattcctttgattcgtacaaggactaataagaccatcttcaaagaaaaaataagaattat
ttttaaagatgtcaaacatccgcatacaattgttttgtatcgggatggtattgtttatttattagtttcagaactttat
tatctaaagttgagtaacaatactataaatcgtttttgcaattgaatatgccgtgaccatttcaaatgctgttagcgtg
aaaagcatgggaacaaacagaccgcagacaattagatataaattcaagatcagagaaaaaggaagaagtgattgatgat
tgtttacccaattggttcaatgactttattcaaattaggaaattaggccattttaatattaacttctgacgaaaagcgg
gagaatcaaccagtgttgtaaatttgtcttaagtcgatgatatttgcaatgttcacgcaatttagatgaatttttcgct
tcgctataagaatgatacatgggcaatcaaccctaacccatagtgcgtagccaatcattttagttcatgctgtcgcaaa
acagaaagggaattgcaaaccctttagcagtagtaagtaaacccttgttataaataatgcttgtacatgaatccggtta
tgttgaaaggactatttttattcatttgatttgggataatgattgtacaacataaaacagcaaaaatagaagaagatc
atggcttattccaaccaatcttgcgtccctcagatatcagcaaaacaactgatacgaaatttattcagtcttccccgta
tattgaaaaagagcattggctggacttgggaaccttaagtgtcggccattacttttatcactagctcttcagactttt
gttcccaaagattctgtccgttatgcccatcttccttatgctcaagcctttgatatt

**Sequence**	**Length (kb)**	**% A+T**	**Function**
Central core : II : 1624556-1625443 x 7	6.1	69.9	yes
Central core : II : 1624556-1625443 x 5	5.5	69.9	yes
6.1kb;*Clostridium sporogenes*;1,351,000-1,357,099	6.1	75.3	yes
6.1kb;*Clostridium sporogenes*;1,432,000-1,438,100	6.1	74.7	yes
6.1kb;*Clostridium sporogenes*;1,231,199-1,234,780	6.1	77.8	yes
4.2kb;*Clostridium sporogenes*; 1,231,199-1,235,400	4.2	79	yes
3.58kb;*Clostridium sporogene*s;1,231,199-1,234,780	3.58	79	yes
5.1kb;*Clostridium acetobutylicum* LJ4;3687849 -3692936	5.1	70.7	weak
wee1_CDEII concatamer_I	5.9	74.4	yes
wee1_CDEII concatamer_II	5.9	74.4	yes
Central core : II : 1624556-1625443 x 8; GC rich	7	61.9	no
Central core : II : 1624556-1625443 x 7; AA/TT > AC/TGn	6	61.9	no
alphoid	5.7	63.1	no
neo-centromere	9.1	67.8	no
Full-length central core	9.46	71.6	yes

Supplementary data table 2. Base sequence composition of sequences assayed and analysed in Figures 2 to 4 of the main text.

Supplementary data table 3. Assaying centromeric activity of centromeric and candidate DNA in the centromere replacement assay

Strain (Nott)	Plasmid	Average colony size	Average recovered	ura-	Proportional Recovery	Proportional Recovery ura-	Site-specific recombination check	PFG integrity check of swapped clones (number intact/number checked)
**Central core sequences and vector control**								
**9.46 kb central core: 1619482-1628933: (Cen 8)**								
593	pREP81	2185000	0.255					
593	pREP81Bxb	3040000	0.304	1	1.66	1.66		
593	pREP81	3157000	0.336					
593	pREP81Bxb	4246000	0.328	0.94	1.73	1.626		
593	pREP81	2344000	0.3					
593	pREP81Bxb	3640000	0.28	1	1.45	1.45		
			sample SD/mean		**0.113**	**1.579**	10/10 (blot), 39/40(long range PCR)	10/10
**6.8 kb central core: 1620855-1627609: (Cen 5)**								
588	pREP81	3300000	0.329					
588	pREP81Bxb	5200000	0.332	0.98	1.59	1.558		
588	pREP81	3940000	0.312					
588	pREP81Bxb	4060000	0.251	0.99	0.83	0.822		
588	pREP81	5160000	0.283					
588	pREP81Bxb	5340000	0.319	0.97	1.17	1.135		
588	pREP81	4720000	0.366					
588	pREP81Bxb	4060000	0.414	0.99	0.97	0.96		
588	pREP81	3480000	0.332					
588	pREP81Bxb	2780000	0.403	1	0.97	0.97		
588	pREP81	2960000	0.29					
588	pREP81Bxb	3920000	0.4	0.97	1.83	1.775		
			sample SD/mean		**0.379**	**1.089**	10/10 (blot), 39/40 (long range PCR)	32/32
**5.48 kb central core: 1622123-1627609: (Cen11)**								
clone 5	pREP81	3560000	0.165					
	pREP81Bxb	2450000	0.236	0.96	0.96	0.92		
clone 5	pREP81	4250000	0.17					
	pREP81Bxb	5010000	0.146	1	1	1		
897	pREP81	3850000	0.25					
	pREP81Bxb	3240000	0.24	0.98	0.98	0.96		
			sample SD/mean		**0.04**	**0.96**	3/24 (long range PCR)	not done

**4.38 kb central core: 1623232-1627609: (Cen12)**								
899	pREP81	2560000	0.2					
	pREP81Bxb	1890000	0.16	0.98	0.59	0.58		
899	pREP81	1030000	0.238					
	pREP81Bxb	1720000	0.195	0.95	1.37	1.3		
899	pREP81	3420000	0.2					
	pREP81Bxb	1350000	0.145	0.97	0.29	0.28		
899	pREP81	5350000	0.21					
	pREP81Bxb	3800000	0.17	0.92	0.57	0.57		
			sample SD/mean		**0.435**	**0.72**	23/23 (long range PCR)	not done
**4.17 kb central core: 1620855-1625025: (Cen1)**								
584	pREP81	923000	0.32					
584	pREP81Bxb	403846	0.304	0.52	0.42	0.218		
584	pREP81	1018182	0.334					
584	pREP81Bxb	360000	0.292	0.82	0.31	0.254		
584	pREP81	1000000	0.392					
584	pREP81Bxb	363636	0.188	0.73	0.17	0.124		
584	pREP81	2530000	0.27					
584	pREP81Bxb	1580000	0.11	0.85	0.25	0.2		
584	pREP81	4700000	0.225					
584	pREP81Bxb	1700000	0.11	0.81	0.18	0.153		
			sample SD/mean		**0.052**	**0.19**	10/10 (blot),<br>40/40 (long range PCR)	26/26
**3.59 kb central core: 1620855-1625025: (Cen2)**								
585	pREP81	4300000	0.23					
585	pREP81Bxb	940000	0.14	0.61	0.13	0.079		
585	pREP81	3470000	0.29					
585	pREP81Bxb	800000	0.13	0.44	0.1	0.044		
585	pREP81	3150000	0.23					
585	pREP81Bxb	2300000	0.081	0.31	0.26	0.081		
585	pREP81	2390000	0.22					
585	pREP81Bxb	1600000	0.075	0.404	0.23	0.093		
585	pREP81	2140000	0.188					
585	pREP81Bxb	730000	0.086	0.41	0.16	0.066		
			sample SD/mean		**0.019**	**0.07**	7/10 (blot),<br>36/36 (long range PCR)	30/30
**3.56 kb central core : 1,620,855-1,624,410 (Cen 23)**								
969	pREP81	3300000	0.39					
	pREP81Bxb	885000	0.081	0.005	**0.06**	**0.0003**		
969	pREP81	3270000	0.367					
	pREP81Bxb	1030000	0.107	0.005	**0.09**	**0.00045**		
969	pREP81	3000000	0.296					
	pREP81Bxb	1320000	0.164	0.005	**0.24**	**0.0012**		
sample SD/mean					0	**0.001**	7/9 long range PCR	

**4.19 kb central core: 1,624,025-1,628,216 (Cen 22)**								
967	pREP81	3570000	0.32					
	pREP81Bxb	1670000	0.18	0.55	0.26	0.143		
967	pREP81	2960000	0.32					
	pREP81Bxb	1760000	0.164	0.56	0.3	0.168		
967	pREP81	3260000	0.32					
	pREP81Bxb	1720000	0.26	0.8	0.43	0.344		
967	pREP81	3040000	0.43					
	pREP81Bxb	1310000	0.31	0.8	0.31	0.248		
967	pREP81	2320000	0.41					
	pREP81Bxb	1350000	0.295	0.8	0.42	0.336		
sample SD/mean					**0.093**	**0.248**		
**2.00 kb central core: 1624025-1626025: (Cen3)**								
591	pREP81	3679000	0.345					
591	pREP81Bxb	510000	0.182	0 (<0.029)	0.07	<0.002		
591	pREP81	2627000	0.461					
591	pREP81Bxb	326000	0.072	0 (<0.029)	0.02	<0.0006		
591	pREP81	4040000	0.335					
591	pREP81Bxb	476000	0.062	0 (<0.029)	0.02	<0.0006		
			sample SD/mean		**0.001**	**0.0009**	5/10 (blot),<br>29/40 (long range PCR)	10/10
**1.00 kb central core: 1624025-1625025: (Cen4)**								
586	pREP81	2800000	0.259					
586	pREP81Bxb	324000	0.083	0.05	0.04	0.002		
586	pREP81	3240000	0.36					
586	pREP81Bxb	288000	0.192	0.02	0.05	0.001		
586	pREP81	2839000	0.288					
586	pREP81Bxb	325000	0.11	0.03	0.04	0.001		
			sample SD/mean		**0.001**	**0.001**	9/10 (blot),<br>39/40 (long range PCR)	10/10
**Vector control**								
597	pREP81	5229000	0.553					
597	pREP81Bxb	134000	0.143	0 (<0.029)	3	<0.0003		
597	pREP81	7380000	0.28					
597	pREP81Bxb	136000	0.095	0 (<0.029)	0.01	<0.0003		
597	pREP81	4160000	0.359					
597	pREP81Bxb	235000	0.148	0.1	0.02	0.02		
597	pREP81	4860000	0.31					
597	pREP81Bxb	1000000	0.105	0.015	0.07	0.001		
597	pREP81	5330000	0.265					
597	pREP81Bxb	960000	0.18	0.01	0.12	0.001		
597	pREP81	4330000	0.29					
597	pREP81Bxb	690000	0.074	0	0.04	0		
597	pREP81	3630000	0.186					
597	pREP81Bxb	460000	0.104	0.07	0.07	0.005		
			sample SD/mean		**0.007**	**0.005**	9/10 (blot), 28/36 (PCR)	38/38

**(1624556-1625443) x 7: 6.2kb**								
821	pREP81	4145000	0.1449					
	pREP81Bxb	5132000	0.232	1	1.98	1.98		
821	pREP81	3448000	0.1882					
	pREP81Bxb	4692000	0.195	1	1.41	1.41		
822	pREP81	3286000	0.25					
	pREP81Bxb	3437000	0.352	1	1.47	1.47		
822	pREP81	3852000	0.259					
	pREP81Bxb	4347000	0.286	1	1.25	1.25		
				sample SD/mean	**0.316**	**1.528**	34/34 (PCR)	16/16
**(1624556-16254430) x 5: 4.4kb**								
892	pREP81	2960000	0.34					
	pREP81Bxb	2210000	0.282	0.98	0.62	0.61		
893	pREP81	2270000	0.25					
	pREP81Bxb	1330000	0.243	0.98	0.57	0.56		
893	pREP81	1540000	0.268					
	pREP81Bxb	1040000	0.286	0.98	0.72	0.71		
				sample SD/mean	**0.076**	**0.627**	22/22 (PCR)	not done
**Experimental sequences**								
**(1624556-16254430) x 7: (A/T)n > (AG/CT)n : (6.3 kb unbent)**								
823/1	pREP81	7800000	0.205					
	pREP81Bxb	1270000	0.07	0.05	0.06	0.003		
823/2	pREP81	5000000	0.195					
	pREP81Bxb	390000	0.056	0 (<0.029)	0.02	<0.002		
824/2	pREP81	3450000	0.143					
	pREP81Bxb	1380000	0.071	0 (<0.029)	0.2	<0.002		
823/1	pREP81	5430000	0.247					
	pREP81Bxb	1940000	0.119	0.02	0.17	0.0034		
823/2	pREP81	3190000	0.201					
	pREP81Bxb	956000	0.068	0 (<0.029)	0.1	<0.002		
823/4	pREP81	4855000	0.253					
	pREP81Bxb	1711000	0.081	0 (<0.029)	0.11	<0.002		
				sample SD/mean	**0.001**	**0.003**	0/5 (PCR)	not done
**(1624556-16254430) x 8: GC rich (7.0kb)**								
860	pREP81	4680000	0.48					
	pREP81Bxb	1090000	0.096	0.15	0.05	0.0075		
861	pREP81	5340000	0.46					
	pREP81Bxb	1130000	0.109	0	0.05	0.001		
860	pREP81	5680000	0.47					
	pREP81Bxb	1190000	0.115	not done but extrapolate 0.07	0.05	0.0035		
860	pREP81	5380000	0.45					
	pREP81Bxb	966000	0.121	not done but extrapolate 0.07	0.05	0.0035		
861	pREP81	5820000	0.48					

	pREP81Bxb	1160000	0.16	not done but extrapolate 0.07	0.07	0.0049		
861	pREP81	5600000	0.49					
	pREP81Bxb	1200000	0.137	not done but extrapolate 0.07	0.06	0.0042		
		sample SD/mean			**0.002**	**0.004**	3/20 (PCR)	not done
**wee1-CDEII concatamer (6.0 kb)**								
819	pREP81	6520000	0.29					
	pREP81Bxb	8860000	0.316	0.98	1.48	1.45		
819	pREP81	5180000	0.1825					
	pREP81Bxb	6020000	0.222	1	1.41	1.41		
820	pREP81	4935000	0.166					
	pREP81Bxb	6568000	0.29	1	2.33	2.33		
820	pREP81	5090000	0.182					
	pREP81Bxb	8110000	0.196	1	1.72	1.72		
		sample SD/mean			**0.425**	**1.728**	28/29 (PCR)	28/28
**Human Y alphoid DNA (5.7kb)**								
496	pREP81	5860000	0.426					
496	pREP81Bxb	1557692	0.127	0 (<0.029)	0.08	< 0.002		
496	pREP81	4540000	0.386					
496	pREP81Bxb	1500000	0.102	0 (<0.029)	0.09	< 0.003		
496	pREP81	6290909	0.401					
496	pREP81Bxb	1269231	0.119	0.01	0.06	0.0006		
					sample mean	**<0.0037**	2/10 blot, 10/40 PCR	10/10
***Clostridium acetobutylycum* (5.1kb)**								
817	pREP81	4680000	0.198					
	pREP81Bxb	1450000	0.09	0.59	0.14	0.083		
817	pREP81	4170000	0.275					
	pREP81Bxb	704000	0.0835	0.28	0.05	0.014		
818	pREP81	3890000	0.271					
	pREP81Bxb	1078000	0.072	0.49	0.07	0.034		
817	pREP81	7360000	0.227					
	pREP81Bxb	1422000	0.075	0.05	0.06	0.003		
818	pREP81	4140000	0.165					
	pREP81Bxb	641000	0.0345	0.42	0.03	0.013		
				sample SD/mean	**0.032**	**0.029**	46/48 (PCR), 24/25 (long range PCR)	17/17
**Neo-centromere (38355754-3864737; 9.0kb)**								
772	pREP81	2610000	0.318					
	pREP81Bxb	510000	0.109	0.023	0.07	0.002		
772	pREP81	870000	0.4					
	pREP81Bxb	490300	0.121	0.0733	0.17	0.012		
772	pREP81	4157000	0.5					
	pREP81Bxb	188000	0.169	0.06	0.02	0.001		

**sample SD/mean**					**0.006**	**0.005**	30/32 (PCR),<br>9/11 (long range PCR)	not done
**Cen 25-6.1kb 78% A+T**								
972	pREP81	2570000	0.225					
	pREP81Bxb	2279000	0.37	1	1.46	1.46		
972	pREP81	1648000	0.263					
	pREP81Bxb	2630000	0.32	1	1.94	1.94		
972	pREP81	850000	0.277					
	pREP81Bxb	2920000	0.314	1	3.89	3.89		
971	pREP81	3030000	0.26					
	pREP81Bxb	2750000	0.32	1	1.12	1.12		
971	pREP81	1270000	0.26					
	pREP81Bxb	1814000	0.272	1	1.49	1.49		
971	pREP81	1800000	0.233					
	pREP81Bxb	1940000	0.226	1	1.05	1.05		
971	pREP81	1016000	0.178					
	pREP81Bxb	2270000	0.26	1	3.26	3.26		
**sample SD/mean**					**1.109**	**2.03**	23/24 (long range PCR)	not done
**Cen 28-6.1kb 75% A+T**								
976	pREP81	2970000	0.222					
	pREP81Bxb	4010000	0.26	1	1.58	1.58		
976	pREP81	3340000	0.24					
	pREP81Bxb	3050000	0.33	1	1.26	1.26		
976	pREP81	1960000	0.295					
	pREP81Bxb	2880000	0.262	1	1.31	1.31		
976	pREP81	1580000	0.22					
	pREP81Bxb	1080000	0.217	1	0.67	0.67		
976	pREP81	2020000	0.278					
	pREP81Bxb	2500000	0.2878	1	1.28	1.28		
976	pREP81	2200000	0.35					
	pREP81Bxb	2840000	0.299	1	1.1	1.1		
**sample SD/mean**					**0.302**	**1.2**	24/24 (long range PCR)	not done
**Cen 29-6.1kb 75% A+T**								
978	pREP81	1640000	0.225					
	pREP81Bxb	1410000	0.234	0.985	0.89	0.87665		
978	pREP81	1470000	0.232					
	pREP81Bxb	1550000	0.234	0.985	1.06	1.0441		
978	pREP81	2050000	0.22					
	pREP81Bxb	980000	0.227	0.985	0.49	0.48265		
978	pREP81	2700000	0.434					
	pREP81Bxb	2480000	0.407	0.99	0.86	0.8514		
978	pREP81	2240000	0.388					
	pREP81Bxb	2300000	0.436	0.99	1.15	1.1385		
978	pREP81	3480000	0.332					
	pREP81Bxb	2080000	0.258	0.99	0.46	0.4554		
**sample SD/mean**					**0.283**	**0.808**		

**Cen 24-0.79 A+T 4.2kb**								
970	pREP81	2730000	0.32					
	pREP81Bxb	2250000	0.3	0.99	0.77	0.7623		
970	pREP81	2180000	0.336					
	pREP81Bxb	2200000	0.198	0.98	0.59	0.5782		
970	pREP81	2100000	0.37					
	pREP81Bxb	1760000	0.162	0.98	0.37	0.3626		
970	pREP81	1320000	0.243					
	pREP81Bxb	1600000	0.154	0.98	0.77	0.7546		
970	pREP81	2360000	0.325					
	pREP81Bxb	2280000	0.182	0.98	0.54	0.5292	24/24 (long range PCR)	not done
				**sample SD/mean**	**0.167**	**0.597**		
**Cen 26-0.79 A+T 3.58kb**								
974, 975	pREP81	3190000	0.45					
	pREP81Bxb	2160000	0.099	0.706	0.15	0.1059		
974, 975	pREP81	3200000	0.446					
	pREP81Bxb	1400000	0.106	0.866	0.1	0.0866		
974, 975	pREP81	3840000	0.445					
	pREP81Bxb	1470000	0.136	0.906	0.12	0.10872	24/24 (long range PCR)	not done
				**sample SD/mean**	**0.012**	**0.1**		
**Controls for centromere adjacency of candidate sequence**								
**803 (cen 8 at BP1)**								
803	pREP81	2600000	0.351					
	pREP81Bxb	330000	0.137	0 (<0.029)	0.05	0 (<0.029)		
803	pREP81	2160000	0.324					
	pREP81Bxb	200000	0.136	0 (<0.029)	0.04	0 (<0.029)		
803	pREP81	3000000	0.324					
	pREP81Bxb	294000	0.17	0 (<0.029)	0.05	0 (<0.029)		
					**sample mean**	**0 (<0.029)**	not done	
**801 (cen8 at neocen)**								
801	pREP81	1200000	0.227					
	pREP81Bxb	125000	0.136	0 (<0.029)	0.06	0 (<0.029)		
801	pREP81	980000	0.195					
	pREP81Bxb	131000	0.1265	0 (<0.029)	0.09	0 (<0.029)		
801	pREP81	950000	0.214					
	pREP81Bxb	144000	0.133	0 (<0.029)	0.09	0 (<0.029)		
					**sample mean**	**0 (<0.029)**	not done	

3 Chromatin immunoprecipitation (ChIP)

3.1 ChIP

ChIP was carried out as previously described (3) using Abcam AB290 anti-GFP antibody and Dynabeads Protein-A (Invitrogen / Thermo Fisher 10001D) for the precipitation of the GFP tagged Cenp-A. We carried out three rounds of CHIP-seq over four years. All the sequencing was done at the Wellcome Trust Centre for Human Genetics in Oxford. Small differences in coverage reflect the different data sets and presumably arise from differences in reagents and platforms over this long period. The causes of these differences have not been studied as the differences themselves do not affect the conclusions.

3.2 Computational analysis of ChIP-seq data

The genomes of the engineered strains were built with Vector NTI® software using the previously sequenced genome of the start strain CBS2777 (3) and the sequence of the plasmids used for engineering. The files used for alignments were in fasta format. The ChIP~seq reads were aligned to the corresponding *S. pombe* genome (as the fasta file), using BWA-mem with standard parameters. Reads mapping in the correct orientations were selected using samtools "flags" to yield a bam file. Coverage vectors were created from the bam files using the R Bioconductor package, the coverage vectors were then normalized on the basis of the number of reads mapping to the centromeres of chromosomes 1 and 3 of the respective strains determined using the samtools view command. . This allowed the results of different experiments to be compared with one another. The plots were then smoothed using the 101or 1001 bp running medians, for the centromeric region or the whole chromosome respectively. The custom fasta files, the coverage vector files (corresponding to each of the figures in the main manuscript and the supplementary data) and the raw data files are present in GEO. The correspondence is listed in table 8 of the supplementary data (attached)

3.3 Results of ChIP-seq analysis discussed but not illustrated in main text

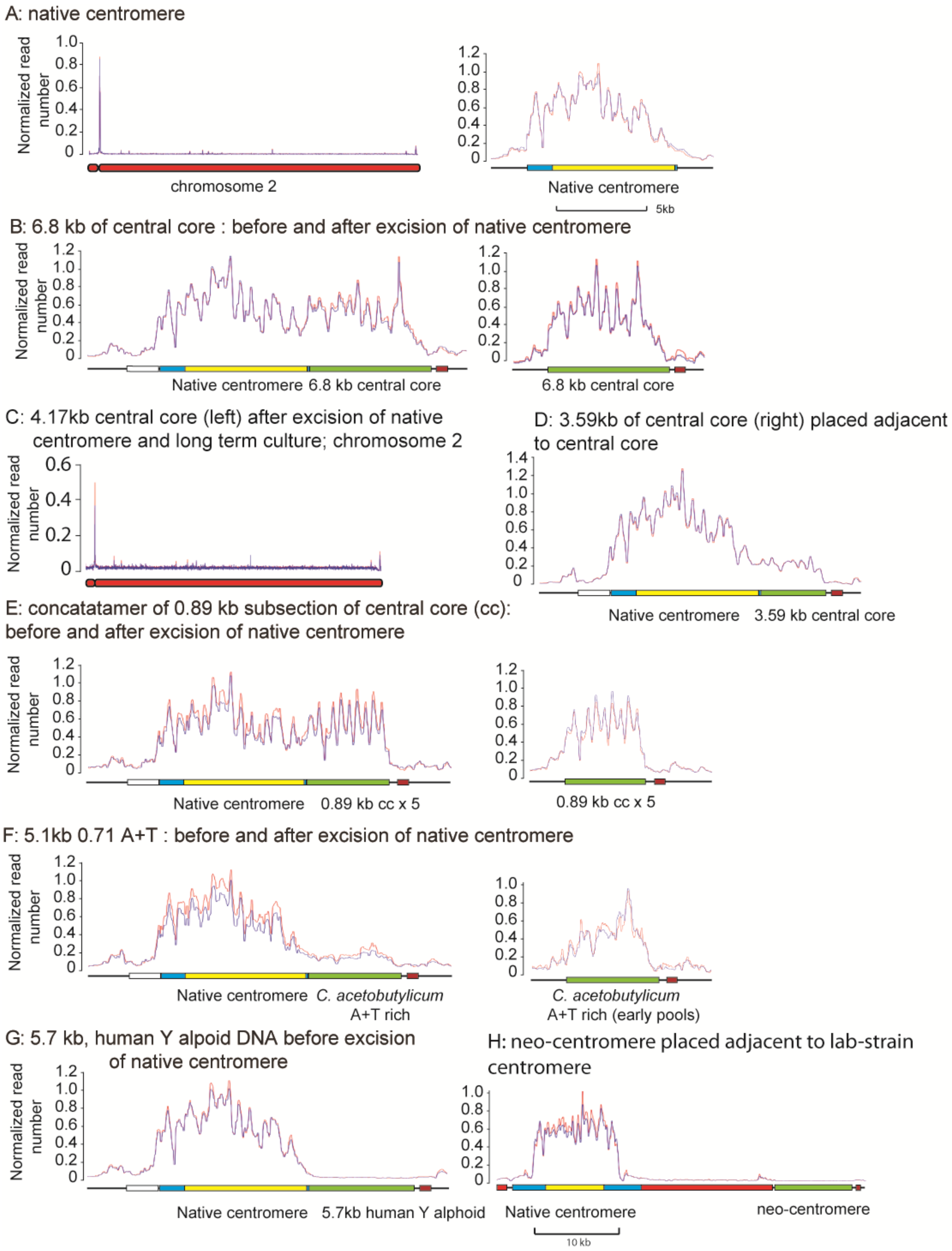

Supplementary data figure 9. CENP-A^{Cnp1} binding to centromere and neo-centromere sequences before and after deletion of the native centromere of chromosome 2 of S. pombe CBS 2777.
A, Binding of CENP-A^{Cnp1} to CBS 2777 chromosome 2 and to its centromere region.
B, Binding of CENP-A^{Cnp1} to 6.8kb of centromere central core sequence placed adjacent to the native centromere of CBS 2777 chromosome 2 before (left panel) and after (right panel) deletion of the native centromere.
C, Binding of CENP-A^{Cnp1} to CBS 2777 chromosome 2 in which the native centromere has been replaced by a 4.17kb centromere central core sequence derived from laboratory strain chromosome II
D, Binding of CENP-A^{Cnp1} to 3.59kb of centromere central core sequence placed adjacent to the native centromere of CBS 2777 chromosome 2 before deletion of the native centromere.
E, Binding of CENP-A^{Cnp1} to a concatamer of five copies of a 889 bp section of centromere central core sequence placed adjacent to the native centromere of CBS 2777 chromosome 2; before (left panel) and after (right panel) deletion of the native centromere. A single clone prior to deletion was analysed in two independent ChIP experiments and two pools of twenty independent clones from a single deletion experiment were analysed in the experiment shown in the right panel.
F, Binding of CENP-A^{Cnp1} to a 5.1kb 0.71 A+T sequence derived from *C. butylicum* placed adjacent to the native centromere of CBS 2777 chromosome 2 before and after replacement.
G, Binding of CENP-A^{Cnp1} to a 5.7kb unit repeat of human Y alphoid DNA placed adjacent to the native centromere 2 of CBS 2777.
H, Binding of CENP-A^{Cnp1} to the centromere of laboratory strain chromosome II containing a copy of the CBS 2777 chromosome 2 neo-centromere region integrated on the right hand side.

4 Centromere replacement in the laboratory strain of *S. pombe*

4.1 Chromosome engineering

The sequence of chromosome manipulations that enabled centromere replacement in the laboratory strain background is set out in Supplementary data figure 10. The details of the primers used in the PCR reactions that confirmed these manipulations are shown in Supplementary data figure 11. The results of these PCR reactions, of a pulsed-field gel demonstrating the integrity of the manipulated chromosomes and of a ChIP-seq analysis demonstrating deletion of the central core and IMR sequences, are in Supplementary data figure 12. Examples for accurate segregation after centromere replacement are shown in Figure 6D of the main text.

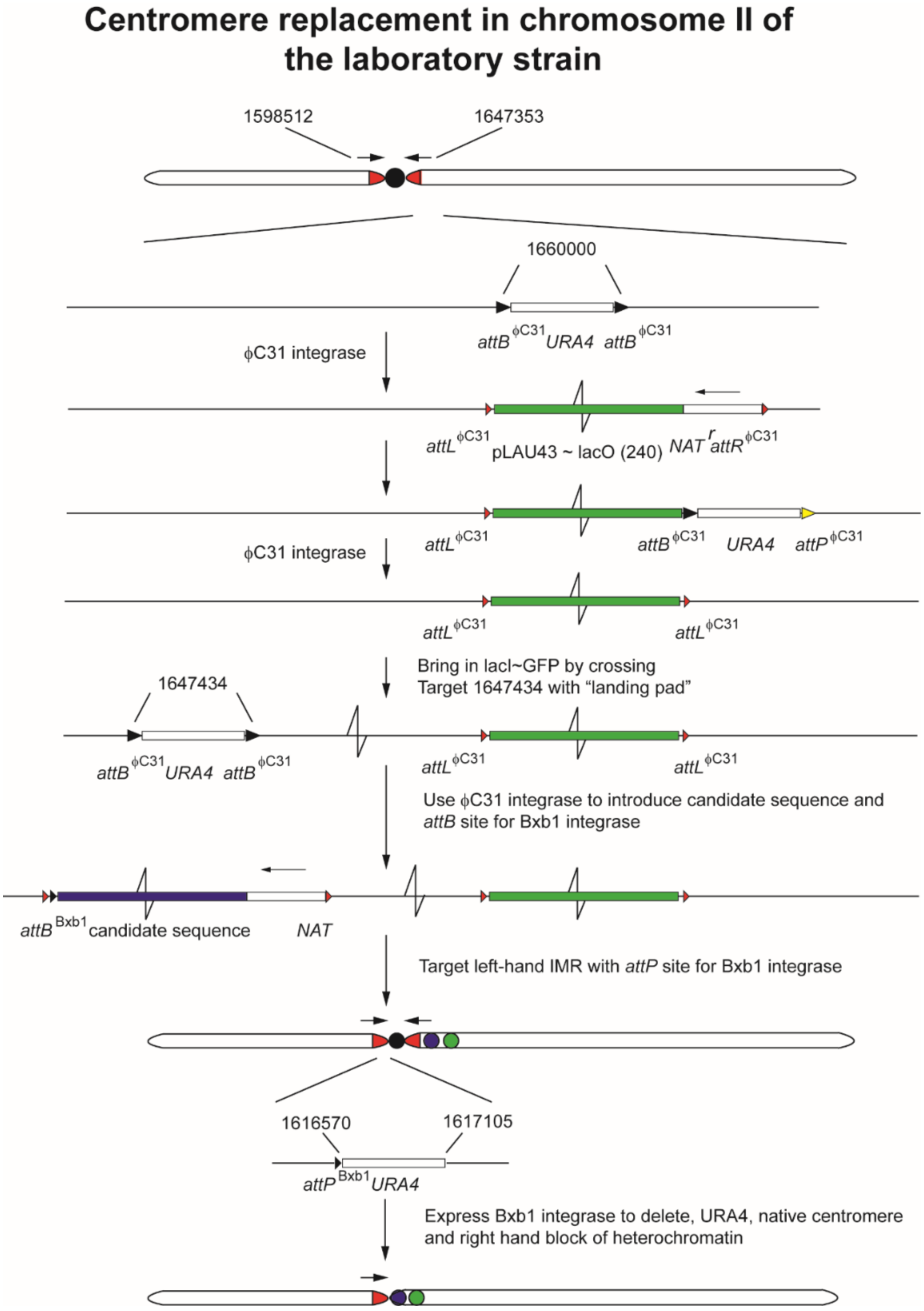

Supplementary data figure 10. Details of centromere replacement in the laboratory strain of *S. pombe*. We started the sequence of chromosome engineering by targeting a *ura4* gene flanked by two *attB* sites for the ϕC31 integrase to residue 1,660,000 on the right hand side of the centromere of chromosome II of a $his7^{-}$ derivative of the laboratory strain of fission yeast; (PN4576 h+ leu1 ura4 his7 alp16Δ::kanR). We used this as a "landing pad" to introduce an array of 240 lac operator sequences derived from plasmid pLAU43 (4) between the *attB* sites using the plasmid pFA6a-natMX6 REV $attP^{\phi C31}$ $attP^{\phi C31}$ as the vector for the incoming sequence. The integration reaction simultaneously deleted the *ura4* gene. We crossed this strain with WRAB_LS8 (h- *ade6*m210 *ura*4Δ18 *leu*1-32) and derived a strain that was (h- his7 chr II:1660000: lacO~240 Nat^{r} ura4 leu1 ade6 kanS). We crossed this strain with SI460 (h+ leu1 ade6 m216 cen2 << lacO<< *ura4+* << kan^{r} *his7+* <<GFP-LacI~nls) and derived a strain Nott979 with the genotype; h90 *his+ ura4- leu1-* chr II:1660000: lacO~240 Nat^{r} $G418^{s}$ This strain was checked by microscopy for segregating GFP fluorescence and was used as the basis of all further manipulations as it contained both the pericentromeric lacO array and the transgene expressing the lacI~GFP ligand necessary for imaging of the centromere of chromosome II. We then targeted the sequences flanking the gene conferring resistance to Nourseothricin with a *ura4* gene flanked by *attP* and *attB* sites for the ϕC31 integrase thus deleting the gene conferring resistance to Nourseothricin. We then deleted the *ura4* gene using expression of the ϕC31 integrase. We introduced a *ura4* gene flanked by two *attB* sites for the ϕC31 integrase into the right hand side of chromosome II at position 1647434 ,12566 residues 5' of the lacO array, to generate strain Nott 986 and used this as a landing pad for 6.1kb of 0.778 A+T DNA cloned in the donor plasmid; pFA6a-natMX6 REV $attP^{\phi C31}$ $attP^{\phi C31}$ $attB^{Bxb1}$ used previously in the experiments with the CBS2777 strain which simultaneously deleted the *ura4* gene and led to the creation of strain Nott 1000. We then targeted a *ura4* gene flanked on its left hand side by an *attB* site for the Bxb1 recombinase between residues 1616570 and 1617105 at the left hand end of the left most IMR in the centromere of chromosome II to generate two independent strain; Nott 1030 and 1031. Expression of the Bxb1 integrase in these strains deleted the *ura4* gene, the centromere of chromosome II and the right hand dgdh array and generated strains Nott 1037 and 1038 respectively (see supplementary data figure 11 for the positions of the primers used these strains) which we used in the imaging experiments shown in figure 6 of the main text. We crossed each of Nott 1037 and 1038 with Nott 1014 ($Ura4^{-}leu1^{-}his7^{-}$ Cnp1~GFP $G418^{r}$ h^{+}) to derive strains Nott 1041 and 1042; which were Nat^{+} $Ura4^{-}leu1^{-}his7^{-}Ade^{-}$ Cnp1~GFP $G418^{r}$ that we used in the ChIP~seq analysis (see table 7 for a more complete genotype).

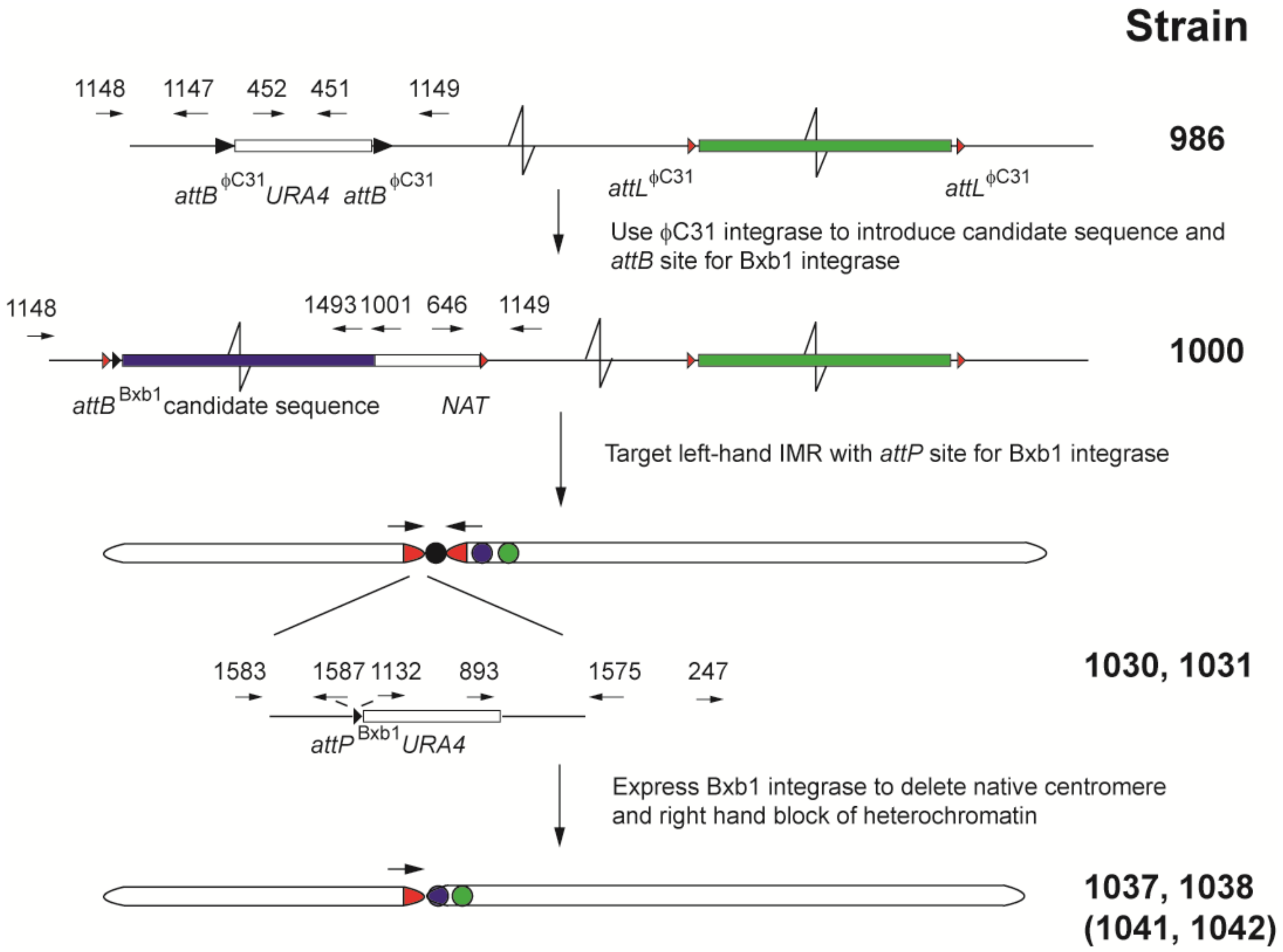

Supplementary data figure 11. Details of the strains and locations of primers used in centromere replacement in the laboratory strain of *S. pombe*

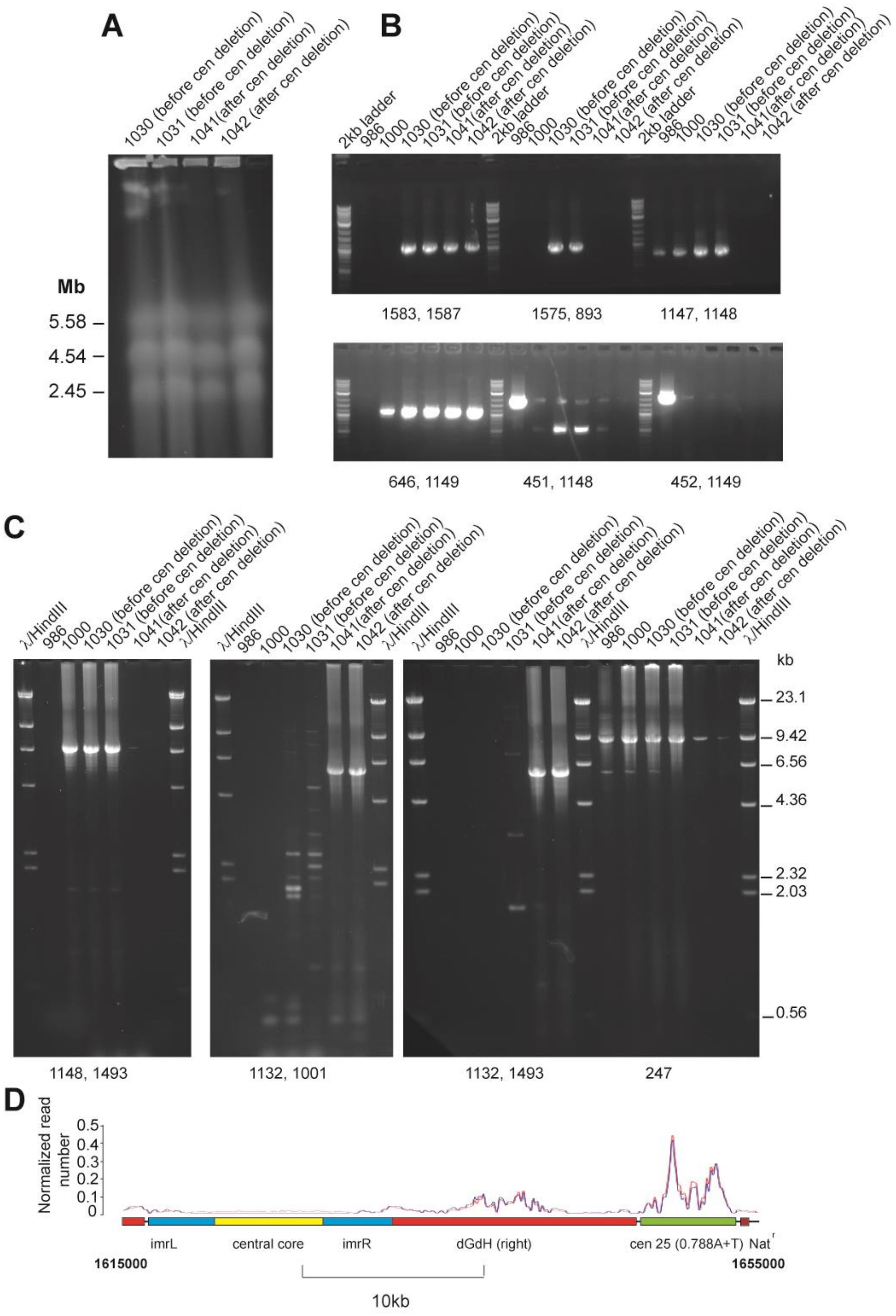

Supplementary data figure 12. Characterization of the laboratory strain centromere replacement.
A, Pulsed field gel characterization of the strains before and after deletion of the native centromere.
B, Characterization of the strains before and after deletion of the native centromere using short range PCR
C, Characterization of the strains before and after deletion of the native centromere using long range PCR. The sequence of primer 1132 is located in the *attP*^{Bxb} site before the recombination breakpoint.
D, Mapping of reads from CENP-A^{Cnp1} ChIP~seq of the strain after deletion onto the sequence of the modified centromeric region of chromosome II of the strain before deletion. Demonstrates no residual binding of CENP-A^{Cnp1} to IMR or central core sequence that might have been present had the re-arrangement been more complex than predicted on the basis of site-specific recombination acting alone.

5 **Linear regression of AT content of 180bp sub-sections of centromere sequences and CENP-A[Cnp1] binding for six sequences**

**Sequence**	**Regresssion coefficient**	**p value**	**R^2**	**Variance of AT content**
Wee1-CDEII concatamer	0.2165	0.534	0.01305	0.00103132
5.1 kb 0.71 A+T *C. acetobutylicum*	0.198	0.851	0.001374	0.00140004
6.1 kb 0.778 A+T *C. sporogenes*	-4.996	2.17 x 10E-5	0.2036	0.00273237
6.1 kb 0.753 A+T *C. sporogenes*	-3.2928	3.15 x 10E-7	0.1485	0.00266918
6.1 kb 0.747 A+T *C. sporogenes*	-0.2414	0.7	0.004839	0.0021785
0.89 kb central core x 7 concatamer	-1.914	5.7 x 10E-9	0.6589	0.00167066
IMRs and central core of lab strain II	0.3459	0.646	0.00261	0.00167066

Supplementary data table 4. Regression of CENP-A[Cnp1] binding on AT content across 180 bp tracts for six sequences described in the main text.

6 Evolutionary analysis of the AT content of centromeric DNA

Supplementary data table 5. Data used in Figure 7 of the main text.

Species	phylum	A+T content centromere	mean genome A+T content	genome size * 10E7 Bp	AT difference	log (genome size)	Comments
Drosophila melanogaster	arthropod	80	57.9	14	22.1	8.146128036	calculated from PMID: 29305387
Drosophila simulans	arthropod	71	57.2	10.5	13.8	8.021189299	calculated from PMID: 29305387
Hymoleptis microstoma	Platyhelminthes	55.9	63	16.3	-7.1	8.212187604	PMID: 33167983
Solenopsis invicta	arthropod	62.4	63.6	39.1	-1.2	8.592176757	PMID:2768313
Ciona intestinalis	ascidian	56.7	64	11.7	-7.3	8.068185862	sequences from Satoh and Satou (personal communication)
Danio rerio	chordata	64.2	63.4	141	0.8	9.149219113	M89944.1 Zebrafish satellite type I DNA: 190bp unit repeat
Takifugu rubripes	chordata	58.5	54.3	38.4	4.2	8.584331224	Sequence present and tandemly repeated
Xenopus laevis (assumed genome size of tropicalis)	chordata	64	59.3	145	4.7	9.161368002	Sequence from PMID: 15673610
Gallus gallus	chordata	50.7	58.1	121	-7.4	9.08278537	AB556722.1, AB556723, AB556724, AB556725, AB556726, AB556727, AB556728
Bos taurus	chordata	33.5	58.1	271.5	-24.6	9.433769834	M36668.1 Bovine 680 bp repeated unit of 1.723 satellite DNA
Equus caballus	chordata	48.9	58.5	247.4	-9.6	9.393399695	GenBank: AH010654.2
Homo sapiens	chordata	62.6	58.7	310	3.9	9.491361694	
Mus musculus	chordata	65.5	58.05	282	7.45	9.450249108	X14469.1 Mouse minor satellite DNA
Oreochromis niloticus	chordata	58.7	60	96	-1.3	8.982271233	X56051.1
Tribolium castaneum	arthropod	75.3	64.6	16.6	10.7	8.220108088	KR046222.1
Sparus aurata	chordata	64	58.1	83	5.9	8.919078092	Z21781.1 S.aurata centromeric satellite DNA
Salmo salar	chordata	66.3	56.3	296	-5.5	9.471291711	AY703447.1 Salmo salar HpaI centromeric satellite sequence
Tetraodon nigroviridis	chordata	59.9	55.4	34.2	4.5	8.534026106	AJ270048

Rattus norvegicus	chordata	61.1	57.6	274	3.5	9.437750563	J00785.1 rat satellite i' dna; 185bp repeat a' (isolated with ecori)
Acomys russatus (spiny mouse)	chordata	57	57.5	231	-0.5	9.36361198	AF079869.1 Acomys russatus clone pArus1 satellite sequence
Oryctolagus cuniculus	chordata	60.5	56	268	4.5	9.428134794	AF527547.1 Oryctolagus cuniculus clone B65 centromeric satellite I sequence
Mucor circinelloides	fungus	71	60.2	3.66	10.8	7.563481085	PMID: 31679929; lacks detectable CenpA or CenpC; includes a 41bp conserved motif
Magnaporthales (Pyricularia) oryzae	fungus	72.2	49	3.88	23.2	7.588831726	PMID:31363034
Candida albicans	fungus	65	66.4	1.47	-1.4	7.167317335	PMID:15272074
Candida tropicalis	fungus	64	67	1.48	-3	7.170261715	PMID:26845548; flanked by 2-5kb inverted repeats
Candida glabrata	fungus	86.4	61.4	1.25	25	7.096910013	PMID: 8917084, GenBank: U43926.1 315-380; flanked by CDEI and III elements
Candida lusitaniae	fungus	67.41	55.5	1.19	11.66	7.075546961	PMID: 26371315.
Candida dubliniensis CD36	fungus	64.1	66.9	3.31	-2.8	7.519827994	PMID: 19060206
Cryptococcus deuterogatti	fungus	56.85	52.1	1.75	4.75	7.243038049	PMID: 29507212
Cryptococcus neoformans	fungus	52	51.8	1.89	0.2	7.243038049	PMID: 29507212
Cryptococcus deneoformans	fungus	52.1	51.8	1.89	0.3	7.276461804	PMID: 29507212
Malassezia sympodialis ATCC42132	fungus	56.6	41.5	0.767	15.1	6.884795364	PMID: 31958060
Neurospora crassa	fungus	70	51.9	4.7	18.1	7.672097858	PMID: 21505064
Schizosaccharomyces pombe	fungus	71.2	64	1.28	7.2	7.10720997	
Saccharomyces cerevisiae	fungus	85	61.7	1.18	23.3	7.071882007	
Yarrowia lipolytica	fungus	71	51	2.2	20	7.342422681	PMID: 11124900
Komagataella phaffii	fungus	60.85	58.9	0.94	1.95	6.973127854	PMID 27497317
Malassezia furfur CBS 14141	fungus	79.76	35.1	1.38	44.66	7.139879086	PMID: 31958060
Malassezia globosa CBS7966	fungus	77.8	47.95	0.8939	29.85	6.951288937	PMID: 31958060
Malassezia slooffiae CBS7956	fungus	73.72	33.7	0.867	40.02	6.938019097	PMID: 31958060
Malassezia restricta CBS877	fungus	66.8	44.27	0.726	22.53	6.860936621	PMID: 31958060
Naumovozyma castelli	fungus	78.6	63.3	1.117	15.3	7.048053173	PMID: 26166782
Naumovozyma dairenensis	fungus	75.1	62.2	1.37567	12.9	7.138514266	PMID: 26166782

Primer	Name	Sequence
106	76:1.2L	gcctaaatcaaaatgaagctga
247	IMR21628928R	caagcttgtaagcataatatgttcaga
420	BP4BamRF	agctggatccgaagcggaaattacttcgtaatag
421	BP4SacXhoRR	agctgagctcgagccttgaattttgaagaaacgcc
422	BP4NotXhoLF1621700R	agctgcggccgcctcgaggtaaatttagaaaatcgtatcc
423	BP4BamLR1620686F	agctggatcctacttcttctcatggaaacattagc
451	URA4probeF	cgttgtcgaggatttcgaccag
452	URA4probeR	acatccaagccgataccaggggac
646	NL197replacement	aagactgtcaaggagggtattctg
784	II:centralcoreXhoF	agctctcgagttacgtactaactgttagtttctatg
785	II:centralcoreXhoR	agctctcgagtacatattagtcagttgctatttag
786	II:cc1624000FXho	agctctcgagaccataaagttttatgataattgttg
789	II:cc16256025RXho	agctctcgagacgatgtgtatgcataacatccaagt
880	1618801LAspBamHF	agctggtaccggatccgtttagctaaattcaaattgac
882	1619636RBglIIF	agctagatctatatttaaattgctaaagtcaatac
883	1620468RSacBamHIR	agctgagctcggatccttctatttttacattaacccaagt
884	1620494Rcheck	gataaaactaattttgtttcgaatc
885	1618772Fcheck	cttcgatactgttaaaagatgtg
892	pBSUra1722F (NL97)	tagagaaagaatgctgagtagat
893	pBSUra24R (NL96)	aaagtttcgtcaatatcacaag
982	1619593LBxBattP'Xho	agctctcgaggtcggggtttgtaccgtacaccactgagaccgcggtggttgacc agacaaaccacgacgtcatttgaattgccaaaaatggt
1001	NL219Long	gattcgatactaacgccgccatccag
1030	Ubp3RSpeXho	agctactagtctcgagcacttgaccctacttttggtact
1132	BxBattPF	gtcgtggtttgtctggtcaaccacc
1147	CenIIRLBamR	agctggatccacagtgattctactttgtgacat
1148	CenIIRLNotXhoF	agctgcggccgctcgagctttccgaattcactgtcattg
1149	CenIIRscreen	caaatttgcaattctcgtggtgtca
1240	BamFCen21624556	agctggatcctttcagagagcctcatattttacg
1241	BglRCen21625443'	agctagatctcgcacatgttgttaatacagttga
1412	2633_mutCenF	agctctcgagcaattgcctaatgtgataactatatc
1413	3734_mutCenF	agctctcgagccttattcttcgagaaacgaaaca
1489	mutCen_8744_R	agctctcgagttaaaatttccatttaatttactc
1490	mutCen_4953_R	agctctcgagtgaatgttgatatttatttattac
1493	C_spor_1237303R	agctctcgagaaattattttaaataacaaaatc
1575	IMRmonitor_R	gcttagtaacgaaaacgtattaagtat
1583	IMRmonitor_F	attgggatagttgaaaaaatg
1587	BxBattP_2	gtggttgaccagacaaaccacgac

Supplementary data table 6. Primers

Strain	Genotype
SW020	h- leu1-32 ura4-D18 kanMX6<<Pcnp1-mEGFP-cnp1+ cen2<<LEU2<<tetO Z<<natR<<Padh31-tetR-tdTomato
Nott 373	CBS2777 h90 chr2 BP4< attB ura4attB ura4Δ leu1Δ
Nott 402	CBS2777 h90 chr2 BP4< TetO Nat Z<<natR<<Padh31-tetR-tdTomato ura4Δ leu1Δ
Nott 986	h90 chrII:1647434: attB ura4attB chrII:1660000 lacO (240) pLau 43 ade6- leu1- ura4- his7:GFPlacI
Nott 1000	h90 chrII:1647434: 6.1kb 0.778 A+T *C sporogenes* Nat, chrII:1660000 lacO (240) pLau 43 ade6- leu1- ura4- his7:GFPlacI
Nott 1037	h90 chrII:Δ 1616570:1647434 : 6.1kb 0.778 A+T *C sporogenes* Nat, chrII:1660000 lacO (240) pLau 43 ade6- leu1- ura4- his7:GFPlacI
Nott 1038	h90 chrII:Δ 1616570:1647434 : 6.1kb 0.778 A+T *C sporogenes* Nat, chrII:1660000 lacO (240) pLau 43 ade6- leu1- ura4- his7:GFPlacI
Nott 1041	chrII:Δ 1616570:1647434 : 6.1kb 0.778 A+T *C sporogenes* Nat, chrII:1660000 lacO (240) pLau 43 ade6- leu1- ura4- Cnp1~GFP G418^{R}
Nott 1042	chrII:Δ 1616570:1647434 : 6.1kb 0.778 A+T *C sporogenes* Nat, chrII:1660000 lacO (240) pLau 43 ade6- leu1- ura4- Cnp1~GFP G418^{R}

Supplementary data, table 7. Genotypes of key strains used in construction or analyses

References

1. Farlow, A., Long, H., Arnoux, S., Sung, W., Doak, T.G., Nordborg, M. and Lynch, M. (2015) The Spontaneous Mutation Rate in the Fission Yeast Schizosaccharomyces pombe. *Genetics*, **201**, 737-744.
2. Long, H., Behringer, M.G., Williams, E., Te, R. and Lynch, M. (2016) Similar Mutation Rates but Highly Diverse Mutation Spectra in Ascomycete and Basidiomycete Yeasts. *Genome Biol Evol*, **8**, 3815-3821.
3. Brown, W.R.A., Thomas, G., Lee, N.C.O., Blythe, M., Liti, G., Warringer, J. and Loose, M.W. (2014) Kinetochore assembly and heterochromatin formation occur autonomously in Schizosaccharomyces pombe. *P Natl Acad Sci USA*, **111**, 1903-1908.
4. Lau, I.F., Filipe, S.R., Soballe, B., Okstad, O.A., Barre, F.X. and Sherratt, D.J. (2003) Spatial and temporal organization of replicating Escherichia coli chromosomes. *Mol Microbiol*, **49**, 731-743.
